# Effects of circadian clock genes and health-related behavior on metabolic syndrome in a Taiwanese population: Evidence from association and interaction analysis

**DOI:** 10.1371/journal.pone.0173861

**Published:** 2017-03-15

**Authors:** Eugene Lin, Po-Hsiu Kuo, Yu-Li Liu, Albert C. Yang, Chung-Feng Kao, Shih-Jen Tsai

**Affiliations:** 1 Institute of Biomedical Sciences, China Medical University, Taichung, Taiwan; 2 Vita Genomics, Inc., Taipei, Taiwan; 3 TickleFish Systems Corporation, Seattle, Western Australia, United States of America; 4 Department of Public Health, Institute of Epidemiology and Preventive Medicine, National Taiwan University, Taipei, Taiwan; 5 Center for Neuropsychiatric Research, National Health Research Institutes, Miaoli County, Taiwan; 6 Department of Psychiatry, Taipei Veterans General Hospital, Taipei, Taiwan; 7 Division of Psychiatry, National Yang-Ming University, Taipei, Taiwan; 8 Department of Agronomy, College of Agriculture & Natural Resources, National Chung Hsing University, Taichung, Taiwan; CNRS, University of Strasbourg, FRANCE

## Abstract

Increased risk of developing metabolic syndrome (MetS) has been associated with the circadian clock genes. In this study, we assessed whether 29 circadian clock-related genes (including *ADCYAP1*, *ARNTL*, *ARNTL2*, *BHLHE40*, *CLOCK*, *CRY1*, *CRY2*, *CSNK1D*, *CSNK1E*, *GSK3B*, *HCRTR2*, *KLF10*, *NFIL3*, *NPAS2*, *NR1D1*, *NR1D2*, *PER1*, *PER2*, *PER3*, *REV1*, *RORA*, *RORB*, *RORC*, *SENP3*, *SERPINE1*, *TIMELESS*, *TIPIN*, *VIP*, and *VIPR2*) are associated with MetS and its individual components independently and/or through complex interactions in a Taiwanese population. We also analyzed the interactions between environmental factors and these genes in influencing MetS and its individual components. A total of 3,000 Taiwanese subjects from the Taiwan Biobank were assessed in this study. Metabolic traits such as waist circumference, triglyceride, high-density lipoprotein cholesterol, systolic and diastolic blood pressure, and fasting glucose were measured. Our data showed a nominal association of MetS with several single nucleotide polymorphisms (SNPs) in five key circadian clock genes including *ARNTL*, *GSK3B*, *PER3*, *RORA*, and *RORB*; but none of these SNPs persisted significantly after performing Bonferroni correction. Moreover, we identified the effect of *GSK3B* rs2199503 on high fasting glucose (P = 0.0002). Additionally, we found interactions among the *ARNTL* rs10832020, *GSK3B* rs2199503, *PER3* rs10746473, *RORA* rs8034880, and *RORB* rs972902 SNPs influenced MetS (P < 0.001 ~ P = 0.002). Finally, we investigated the influence of interactions between *ARNTL* rs10832020, *GSK3B* rs2199503, *PER3* rs10746473, and *RORB* rs972902 with environmental factors such as alcohol consumption, smoking status, and physical activity on MetS and its individual components (P < 0.001 ~ P = 0.002). Our study indicates that circadian clock genes such as *ARNTL*, *GSK3B*, *PER3*, *RORA*, and *RORB* genes may contribute to the risk of MetS independently as well as through gene-gene and gene-environment interactions.

## Introduction

Circadian rhythms are naturally recurring cycles that regulate the timing of biological events such as the sleep-wake cycle and energy metabolism [1]. The intracellular molecular machinery underlying circadian rhythms implicates that circadian oscillations are controlled and maintained by a set of core circadian clock genes, including the aryl hydrocarbon receptor nuclear translocator like (*ARNTL*), clock circadian regulator (*CLOCK*), cryptochrome circadian clock 1 (*CRY1*), cryptochrome circadian clock 2 (*CRY2*), neuronal PAS domain protein 2 (*NPAS2*), period circadian clock 1 (*PER1*), period circadian clock 2 (*PER2*), and period circadian clock 3 (*PER3*) genes [2]. The circadian clock genes are prominently involved in the regulation of metabolism and energy homeostasis because circadian rhythms in turn impact on metabolic activity and metabolism [3]. Circadian disruptions have been well documented in adverse effects on human health, influencing lipid and glucose homeostasis, inflammation, and cardiovascular functions [4].

Accumulating evidence indicates that the circadian clock genes may contribute to the development of metabolic syndrome (MetS). Animal studies showed that a loss-of-function mutation of the circadian clock genes, such as *Clock* and *Arntl*, may result in metabolic abnormalities of lipid and glucose homeostasis, hallmarks of MetS [5, 6]. In humans, evidence has been reported for a potential association of MetS with the *CLOCK* [7] and *CRY2* [8] genes. Previously, Scott et al. showed that a three-marker haplotype (rs4864548, rs3736544, and rs1801260) of the *CLOCK* gene was associated with MetS, although there were no significant associations between any of these three SNPs and MetS individually [7]. Additionally, Kovanen et al. indicated that *CRY2* rs75065406 had a nominally significant association with MetS, but this association did not remain significant after correcting for multiple testing [8]. Moreover, circadian clock genes have been shown to link with the individual components of MetS. Woon et al. found that a three-marker haplotype (rs6486121, rs3789327, and rs969485) of the *ARNTL* gene was associated with hypertension, an individual component of MetS [9]. Further, Englund et al. reported that *NPAS2* rs11541353 was associated with hypertension and *PER2* #10870 was linked to blood glucose levels [10]. Kovanen et al. also indicated a nominally significant association of elevated blood pressure with the *CRY1* haplotype (rs4964513 and rs12821586); however, the significance did not persist after adjusting for multiple testing [8]. In addition, it has been suggested that circadian clock genes such as *ARNTL*, *CRY1*, and *PER2* are expressed in human adipose tissue, and their gene expressions were related to the individual components of MetS such as waist circumference [11]. Furthermore, several animal studies indicated that metabolic complications such as MetS, dyslipidemia, glucose intolerance, hypoinsulinaemia, and diabetes can result from deletion of the *Arntl*, *Clock*, or *Per3* genes, suggesting miscommunication between the circadian clock and metabolic pathways may lead to metabolic disorders [12–14].

While there have been several studies examining the relationship between single nucleotide polymorphisms (SNPs) in the circadian clock genes and MetS, to our knowledge, none have been evaluated in terms of gene-gene interactions. Furthermore, the interplay between the circadian clock genes and health-related behaviors, such as alcohol consumption, smoking status, and physical activity, has not been fully assessed in previous association studies. Given that gene-gene and gene-environment interactions may play a pivotal role in the development of MetS, we hypothesized that the circadian clock genes may contribute to the etiology of MetS and its individual components independently and/or through complex interactions. The gene panel consisted of 29 circadian clock-relevant genes (S1 Table) selected from the literature [7–11,15–21], including the adenylate cyclase activating polypeptide 1 (*ADCYAP1*), *ARNTL*, *ARNTL2*, basic helix-loop-helix family member e40 (*BHLHE40*), *CLOCK*, *CRY1*, *CRY2*, casein kinase 1 delta (*CSNK1D*), casein kinase 1 epsilon (*CSNK1E*), glycogen synthase kinase 3 beta (*GSK3B*), hypocretin receptor 2 (*HCRTR2*), Kruppel like factor 10 (*KLF10*), nuclear factor interleukin 3 regulated (*NFIL3*), *NPAS2*, nuclear receptor subfamily 1 group D member 1 (*NR1D1*), nuclear receptor subfamily 1 group D member 2 (*NR1D2*), *PER1*, *PER2*, *PER3*, REV1 DNA directed polymerase (*REV1*), RAR related orphan receptor A (*RORA*), RAR related orphan receptor B (*RORB*), RAR related orphan receptor C (*RORC*), SMT3 specific peptidase 3 (*SENP3*), serpin family E member 1 (*SERPINE1*), timeless circadian clock (*TIMELESS*), TIMELESS interacting protein (*TIPIN*), vasoactive intestinal peptide (*VIP*), and vasoactive intestinal peptide receptor 2 (*VIPR2*) genes.

## Materials and methods

### Study population

This study incorporated Taiwanese subjects from the Taiwan Biobank, which gathered information and specimens from participants in recruitment centers across Taiwan in 2013–2015 [22–27]. The Taiwan Biobank was mainly funded by the Taiwan government and aimed to facilitate collaboration for public health-related research concerning local common chronic diseases [22]. The study cohort consisted of 3,000 participants. Inclusion criteria were individuals who were aged 30 years or over and were self-reported as being of Taiwanese Han Chinese ancestry [24]. Participants with a history of cancer were excluded [24]. Ethical approval for the study was granted by the Institutional Review Board of the Taiwan Biobank before conducting the study (approval number: 201506095RINC). Each subject signed the approved informed consent form. All experiments were performed in accordance with relevant guidelines and regulations.

Current alcohol drinkers were defined as persons who reported drinking more than 150 ml of alcohol per week during the last six months [23]. Current smokers were defined as persons who reported smoking every day or some days during the last six months [23]. Physical activity was defined by the amount of excise activity for more than three times and more than 30 minutes each time in each week.

### Metabolic syndrome

Measurements of metabolic traits (including as waist circumference, triglyceride, high-density lipoprotein cholesterol, systolic and diastolic blood pressure, and fasting glucose) were obtained when participants underwent general health examinations [22–24]. To assess MetS, we used the International Diabetes Federation (IDF) definition, which requires that the participant represented by central obesity (defined as waist circumference ≥ 90 cm in male subjects and ≥ 80 cm in female subjects) plus the presence of two or more of the following four components: (1) triglycerides ≥ 150 mg/dl; (2) HDL cholesterol < 40 mg/dl in male subjects and < 50 mg/dl in female subjects; (3) systolic blood pressure ≥ 130 mmHg or diastolic blood pressure ≥ 85 mmHg; and (4) fasting plasma glucose ≥ 100 mg/dl [28]. Blood pressure was based on the average of two measurements.

### Genotyping

DNA was isolated from blood samples using a QIAamp DNA blood kit following the manufacturer’s instructions (Qiagen, Valencia, CA, USA). The quality of the isolated genomic DNA was evaluated using agarose gel electrophoresis, and the quantity was determined by spectrophotometry [29]. SNP genotyping was carried out using the custom Taiwan BioBank chips and run on the Axiom Genome-Wide Array Plate System (Affymetrix, Santa Clara, CA, USA). To efficiently obtain maximal genetic information from Taiwanese Han Chinese samples, the custom Taiwan BioBank chips were designed by using SNPs on the Axiom Genome-Wide CHB 1 Array (Affymetrix, Inc., Santa Clara, CA, USA) with minor allele frequencies (MAFs) ≥ 5%, by using SNPs in exons with MAFs > 10% on the Human Exome BeadChip (Illumina, Inc., San Diego, CA, USA), and by using SNPs previously reported in ancestry information panels, cancer studies, and pharmacogenetic studies. [24]. In this study, the SNP panel consisted of variants from the following 29 circadian clock genes: *ADCYAP1*, *ARNTL*, *ARNTL2*, *BHLHE40*, *CLOCK*, *CRY1*, *CRY2*, *CSNK1D*, *CSNK1E*, *GSK3B*, *HCRTR2*, *KLF10*, *NFIL3*, *NPAS2*, *NR1D1*, *NR1D2*, *PER1*, *PER2*, *PER3*, *REV1*, *RORA*, *RORB*, *RORC*, *SENP3*, *SERPINE1*, *TIMELESS*, *TIPIN*, *VIP*, and *VIPR2*.

### Statistical analysis

Categorical data were evaluated using the chi-square test. We conducted the Student’s t test to compare the difference in the means from two continuous variables. To estimate the association of the investigated SNP with MetS, we conducted a logistic regression analysis to evaluate the odds ratios (ORs) and their 95% confidence intervals (CIs), adjusting for covariates including age and gender [30]. Furthermore, we estimated the association of the investigated SNP with individual components of MetS by using logistic regression analysis, adjusting for age and gender [31]. The genotype frequencies were assessed for Hardy-Weinberg equilibrium using a χ^2^ goodness-of-fit test with 1 degree of freedom (i.e. the number of genotypes minus the number of alleles). Multiple testing was adjusted by the Bonferroni correction. To address the issue of multiple testing, we also calculated Q values and false discovery rates (FDRs) using the qvalue function in an R package [32]. The criterion for significance was set at P < 0.05 for all tests. Data are presented as the mean ± standard deviation.

To investigate gene-gene and gene-environment interactions, we employed the generalized multifactor dimensionality reduction (GMDR) method [33]. We tested two-way up to four-way interactions using 10-fold cross-validation. The GMDR software provides some output parameters, including the testing accuracy and empirical P values, to assess each selected interaction. Moreover, we provided age and gender as covariates for gene-gene and gene-environment interaction models in our interaction analyses. Permutation testing obtains empirical P values of prediction accuracy as a benchmark based on 1,000 shuffles. In order to correct for multiple testing, we applied a conservative Bonferroni correction factor for the number of tests employed in the GMDR analysis.

Based on the effect sizes in this study, the power to detect significant associations was evaluated by QUANTO software (http://biostats.usc.edu/Quanto.html).

## Results

Table 1 describes the demographic and clinical characteristics of the study population, including 533 MetS subjects and 2,467 non-MetS subjects. The MetS prevalence in our cohort was 17.8%. As shown in Table 1, the distribution was well matched for gender, but not for age. Moreover, there was a significant difference in waist circumference, triglyceride, HDL, systolic blood pressure, diastolic blood pressure, and fasting glucose between the MetS and non-MetS subjects (Table 1; all P < 0.0001, respectively). Furthermore, there was a significant difference in current alcohol consumption (P = 0.029) and smoking status (P = 0.001) between the MetS and non-MetS subjects. However, no significant difference was found between participants with and without the MetS in level of physical activity.

**Table 1:** Demographic and clinical characteristics of study subjects.

Characteristic	MetS	No MetS	P value
No. of subjects (n)	533	2467	
Age (years)	53.3±10.1	48.3±10.9	< 0.0001
Gender (Male %)	48.2%	46.1%	0.371
Waist circumference (cm)	94.0±8.8	82.3±8.8	< 0.0001
Triglyceride (mg/dl)	196.7±142.3	99.6±57.2	< 0.0001
HDL (mg/dl)	43.8±9.2	55.9±12.9	< 0.0001
Systolic blood pressure (mmHg)	126.9±17.8	112.8±15.6	< 0.0001
Diastolic blood pressure (mmHg)	77.3±11.0	70.2±10.3	< 0.0001
Fasting glucose (mg/dl)	114.3±35.4	93.8±15.4	< 0.0001
Physical activity (%)	57.9%	58.8%	0.733
Current smoker (%)	14.6%	9.8%	0.001
Current alcohol drinker (%)	9.8%	7.0%	0.029

HDL = high-density lipoprotein cholesterol, MetS = metabolic syndrome, SD = standard deviation. Data are presented as mean ± standard deviation.

Among the 881 SNPs assessed in this study (S1 Table), there were 88 SNPs in the 13 circadian clock genes showing an evidence of association (P < 0.05) with MetS as shown in S2 Table. However, none of the SNPs were significantly associated with MetS after Bonferroni correction (P < 0.05/881 = 6 x 10^−5^). The related Q values and FDRs were listed in S3 Table. Then, we identified a nominal association (P < 0.01) of MetS with 16 SNPs, including *ARNTL* rs10832020, *GSK3B* rs2199503, *PER3* (rs10746473, rs2797685, rs1689904, rs1773138), *RORA* (rs17237367, rs58469372, rs12591650, rs12594188, rs17270446, rs11630062, rs8029848, rs8034880, rs72752802), and *RORB* rs972902 (Table 2). For further investigating in the subsequent analyses, the five key SNPs in the five circadian clock genes were selected by using the best SNP from each gene, including *ARNTL* rs10832020 (P = 0.0065), *GSK3B* rs2199503 (P = 0.007), *PER3* rs10746473 (P = 0.001), *RORA* rs8034880 (P = 0.0002), and *RORB* rs972902 (P = 0.0087). In addition, the genotype frequency distributions for the *ARNTL* rs10832020, *GSK3B* rs2199503, *PER3* rs10746473, *RORA* rs8034880, and *RORB* rs972902 SNPs were in accordance with the Hardy–Weinberg equilibrium among the subjects (P = 0.261, 0.35, 0.253, 0.76, and 0.594, respectively).

**Table 2:** Odds ratio analysis with odds ratios after adjustment for covariates (including age and gender) between the MetS and 16 SNPs in five selective circadian clock genes, which have nominal evidence of association (P < 0.01).

				Additive model			Dominant model			Recessive model		
Gene	SNP	Alleles		OR	95% CI	P	OR	95% CI	P	OR	95% CI	P
*ARNTL*	rs10832020	C	T	1.23	1.05–1.45	0.0114	1.06	0.87–1.28	0.5586	1.54	1.13–2.09	**0.0065**
*GSK3B*	rs2199503	T	C	0.83	0.70–0.99	0.0331	1.04	0.85–1.26	0.7245	0.64	0.47–0.89	**0.0070**
*PER3*	rs10746473	G	A	1.20	1.04–1.38	0.0116	1.46	1.17–1.83	**0.0010**	1.11	0.88–1.40	0.3691
	rs2797685	C	T	1.17	1.01–1.35	0.0314	1.41	1.11–1.78	**0.0047**	1.07	0.85–1.33	0.5731
	rs1689904	C	T	1.16	1.01–1.34	0.0352	1.39	1.10–1.76	**0.0061**	1.07	0.86–1.33	0.5618
	rs1773138	T	C	0.85	0.74–0.99	0.0304	0.94	0.76–1.17	0.5902	0.71	0.56–0.90	**0.0043**
*RORA*	rs17237367	A	G	0.82	0.68–0.98	0.0263	0.75	0.62–0.91	**0.0036**	0.74	0.53–1.05	0.0926
	rs58469372	A	G	0.82	0.71–0.95	**0.0092**	0.85	0.70–1.04	0.1113	0.71	0.54–0.93	0.0129
	rs12591650	A	G	1.20	1.04–1.38	0.0109	1.39	1.11–1.75	**0.0049**	1.16	0.93–1.44	0.1948
	rs12594188	C	T	1.21	1.01–1.45	0.0389	1.33	1.10–1.61	**0.0035**	1.32	0.92–1.88	0.1272
	rs17270446	G	C	0.97	0.70–1.35	0.8455	1.37	1.12–1.68	**0.0021**	0.84	0.44–1.63	0.6109
	rs11630062	C	T	0.92	0.80–1.05	0.2267	0.76	0.62–0.93	**0.0082**	1.02	0.80–1.30	0.8834
	rs8029848	G	A	1.28	1.05–1.56	0.0127	1.41	1.17–1.71	**0.0004**	1.43	0.98–2.09	0.0629
	rs8034880	G	A	1.29	1.05–1.58	0.0150	1.44	1.19–1.74	**0.0002**	1.44	0.97–2.14	0.0711
	rs72752802	C	A	0.81	0.69–0.94	**0.0053**	0.87	0.72–1.06	0.1716	0.67	0.50–0.88	**0.0045**
*RORB*	rs972902	A	G	0.72	0.55–0.96	0.0227	0.76	0.62–0.93	**0.0087**	0.57	0.33–0.98	0.0426

CI = confidence interval, MetS = metabolic syndrome, OR = odds ratio. Analysis was obtained after adjustment for covariates including age and gender. P values of < 0.01 are shown in bold.

Moreover, the OR analysis showed risk genotypes of variants of *ARNTL* rs10832020, *GSK3B* rs2199503, *PER3* rs10746473, *RORA* rs8034880, and *RORB* rs972902 after adjusting for covariates, indicating an increased MetS risk among the subjects (Table 2). As demonstrated in Table 2 for the *RORA* rs8034880 SNP, there was an indication of an increased MetS risk among the MetS and non-MetS subjects after adjustment of covariates such as age and gender for genetic models, including the dominant model (OR = 1.44; 95% CI = 1.19–1.74; P = 0.0002). Similarly, there was an indication of an increased risk of MetS among the subjects after adjustment of covariates for genetic models in the *ARNTL* rs10832020, *GSK3B* rs2199503, *PER3* rs10746473, and *RORB* rs972902 SNPs (Table 2).

Next, Table 3 and S4 Table show the OR analysis of the *ARNTL* rs10832020, *GSK3B* rs2199503, *PER3* rs10746473, *RORA* rs8034880, and *RORB* rs972902 SNPs with the individual components of MetS including (a) high waist circumference vs. normal waist circumference subjects; (b) high triglyceride vs. normal triglyceride subjects; (c) low HDL vs. normal HDL subjects; (d) high blood pressure vs. normal blood pressure subjects; and (e) high fasting glucose vs. normal fasting glucose subjects. As shown in Table 3 for the *GSK3B* rs2199503 (P = 0.0002), *RORA* rs8034880 (P = 0.02), and *RORB* rs972902 (P = 0.015) SNPs, there was an indication of an increased risk of high fasting glucose among the subjects after adjustment of covariates for genetic models. The effect of *GSK3B* rs2199503 on high fasting glucose remained significant after Bonferroni correction (P < 0.05/75 = 0.0007). Additionally, there was a significantly increased risk of high waist circumference (P = 0.0013) among the subjects for *PER3* rs10746473; however, this association did not persist after Bonferroni correction (P < 0.05/75 = 0.0007).

**Table 3:** Odds ratio analysis with odds ratios after adjustment for covariates between individual components of the MetS and five key SNPs in the five circadian clock genes (including *ARNTL* rs10832020, *GSK3B* rs2199503, *PER3* rs10746473, *RORA* rs8034880, and *RORB* rs972902).

Individual components of the MetS	Additive model			Recessive model			Dominant model		
	OR	95% CI	P	OR	95% CI	P	OR	95% CI	P
(1) *ARNTL* rs10832020									
High waist circumference^a^	1.10	0.96–1.26	0.1710	1.27	0.97–1.65	0.0781	0.95	0.82–1.10	0.5051
High triglyceride^b^	1.04	0.88–1.22	0.6536	1.11	0.82–1.52	0.4951	0.95	0.80–1.14	0.6016
Low HDL^c^	1.13	0.97–1.32	0.1060	1.29	0.97–1.73	0.0827	1.03	0.87–1.22	0.7361
High blood pressure^d^	1.04	0.88–1.22	0.6578	1.06	0.77–1.44	0.7394	1.05	0.88–1.26	0.5973
High fasting glucose^e^	1.09	0.93–1.28	0.2637	1.15	0.85–1.55	0.3694	1.11	0.93–1.32	0.2518
(2) *GSK3B* rs2199503									
High waist circumference^a^	1.08	0.96–1.22	0.1957	1.13	0.91–1.41	0.2726	1.08	0.93–1.26	0.2908
High triglyceride^b^	1.00	0.86–1.15	0.9832	0.98	0.75–1.28	0.8713	1.03	0.86–1.24	0.7717
Low HDL^c^	0.99	0.86–1.14	0.9189	0.89	0.69–1.15	0.3697	1.16	0.97–1.38	0.0966
High blood pressure^d^	0.97	0.83–1.12	0.6378	0.94	0.71–1.23	0.6489	0.98	0.81–1.17	0.7933
High fasting glucose^e^	0.75	0.64–0.87	**0.0002**	0.58	0.43–0.78	**0.0003**	0.84	0.70–1.00	0.0514
(3) *PER3* rs10746473									
High waist circumference^a^	1.19	1.07–1.32	0.0018	1.20	1.00–1.44	0.0466	1.31	1.11–1.55	0.0013
High triglyceride^b^	0.99	0.87–1.13	0.8394	0.92	0.74–1.15	0.4812	1.05	0.86–1.29	0.6252
Low HDL^c^	1.14	1.01–1.29	0.0354	1.14	0.93–1.40	0.2046	1.24	1.03–1.51	0.0266
High blood pressure^d^	1.11	0.97–1.26	0.1317	1.11	0.90–1.38	0.3315	1.17	0.96–1.44	0.1283
High fasting glucose^e^	0.95	0.84–1.09	0.4602	0.80	0.64–1.00	0.0453	1.12	0.92–1.36	0.2613
(4) *RORA* rs8034880									
High waist circumference^a^	1.04	0.88–1.23	0.6372	1.02	0.73–1.42	0.9029	1.16	1.00–1.35	0.0492
High triglyceride^b^	1.09	0.89–1.32	0.4231	1.14	0.77–1.69	0.5084	1.10	0.91–1.31	0.3284
Low HDL^c^	1.14	0.94–1.37	0.1781	1.27	0.88–1.82	0.2014	1.08	0.91–1.28	0.4034
High blood pressure^d^	1.19	0.97–1.45	0.0877	1.33	0.90–1.96	0.1515	1.20	1.00–1.44	0.0525
High fasting glucose^e^	1.02	0.83–1.25	0.8581	0.95	0.63–1.42	0.7908	1.23	1.03–1.47	0.0200
(5) *RORB* rs972902									
High waist circumference^a^	1.03	0.86–1.23	0.7766	1.08	0.76–1.53	0.6667	0.94	0.81–1.10	0.4478
High triglyceride^b^	0.88	0.70–1.11	0.2955	0.83	0.53–1.31	0.4277	0.83	0.68–1.00	0.0463
Low HDL^c^	0.88	0.71–1.09	0.2494	0.80	0.52–1.22	0.3014	0.90	0.76–1.08	0.2506
High blood pressure^d^	0.81	0.63–1.04	0.0958	0.63	0.39–1.03	0.0648	1.06	0.88–1.28	0.5278
High fasting glucose^e^	0.74	0.57–0.94	0.0150	0.55	0.34–0.90	0.0174	0.89	0.74–1.07	0.2239

CI = confidence interval, HDL = high-density lipoprotein cholesterol, MetS = metabolic syndrome, OR = odds ratio. Analysis was obtained after adjustment for covariates including age and gender. P values of < 0.0007 (Bonferroni correction: 0.05/75) are shown in bold.

^a^ Waist circumference ≥ 90 cm in male subjects, waist circumference ≥ 80 cm in female subjects.

^b^ Triglyceride ≥ 150 mg/dl.

^c^ HDL< 40 mg/dl in male subjects, HDL < 50 mg/dl in female subjects.

^d^ Systolic blood pressure ≥ 130 mmHg or diastolic blood pressure ≥ 85 mmHg.

^e^ Fasting glucose ≥ 100 mg/dl.

In addition, the GMDR analysis was used to assess the impacts of combinations between the five SNPs in MetS and its individual components including age and gender as covariates. Table 4 and S5 Table summarize the results obtained from GMDR analysis for two-way gene-gene interaction models in influencing MetS with covariate adjustment. As shown in Table 4, there was a significant two-way model involving *ARNTL* rs10832020 and *RORA* rs8034880 (P < 0.001), *GSK3B* rs2199503 and *RORA* rs8034880 (P < 0.001), *GSK3B* rs2199503 and *RORB* rs972902 (P = 0.002), *PER3* rs10746473 and *RORA* rs8034880 (P < 0.001), and *PER3* rs10746473 and *RORB* rs972902 (P < 0.001). The effects of these two-way models remained significant after Bonferroni correction (P < 0.05/10 = 0.005), indicating a potential gene-gene interaction between *RORA* and *ARNTL*, between *RORA* and *GSK3B*, between *RORA* and *PER3*, between *RORB* and *GSK3B*, and between *RORB* and *PER3* in influencing MetS. Similarly, there were two-way gene-gene interaction models in influencing individual components such as high waist circumference (P = 0.003) or high fasting glucose (P = 0.007) as shown in S6 Table. There were also a three-way model (P = 0.003) in influencing MetS and a four-way model (P = 0.004) in influencing low HDL (S6 Table). However, the effects for these models did not persist after Bonferroni correction (P < 0.05/18 = 0.0028).

**Table 4:** Two-way gene-gene interaction models by using the GMDR method with adjustment for age and gender.

Phenotype	Two-way interaction model	Testing accuracy (%)	P value
MetS	*ARNTL* rs10832020, *GSK3B* rs2199503	52.92	0.039
	*ARNTL* rs10832020, *PER3* rs10746473	53.33	0.021
	*ARNTL* rs10832020, *RORA* rs8034880	55.01	**< 0.001**
	*ARNTL* rs10832020, *RORB* rs972902	52.92	0.032
	*GSK3B* rs2199503, *PER3* rs10746473	53.15	0.027
	*GSK3B* rs2199503, *RORA* rs8034880	55.09	**< 0.001**
	*GSK3B* rs2199503, *RORB* rs972902	54.04	**0.002**
	*PER3* rs10746473, *RORA* rs8034880	54.78	**< 0.001**
	*PER3* rs10746473, *RORB* rs972902	55.19	**< 0.001**
	*RORA* rs8034880, *RORB* rs972902	53.96	0.008

GMDR = generalized multifactor dimensionality reduction, MetS = metabolic syndrome. P value was based on 1,000 permutations. Analysis was obtained after adjustment for covariates including age and gender. P values of < 0.005 (Bonferroni correction: 0.05/10) are shown in bold.

Furthermore, Table 5 and S7 Table show the GMDR analysis of gene-environment interaction models in MetS and its individual components using age and gender as covariates. As shown in Table 5 for MetS, there were a significant two-way model involving *RORB* rs972902 and smoking (P < 0.001). The effect of the two-way model remained significant after Bonferroni correction (P < 0.05/18 = 0.0028), indicating a potential gene-environment interaction between *RORB* and smoking in influencing MetS. Additionally, there was a significant three-way model (P = 0.001) after Bonferroni correction (P < 0.0028), indicating a potential gene-environment interaction among *PER3*, *RORB*, and smoking in influencing MetS. Similarly, there were significant two-way up to four-way gene-environment interaction models (all P < 0.001) in influencing individual components such as low HDL after Bonferroni correction (P < 0.0028). In addition, there were significant three-way and four-way models (P = 0.001 and 0.002, respectively) in influencing high fasting glucose after Bonferroni correction (P < 0.0028).

**Table 5:** Gene-environment interaction models identified by the GMDR method with adjustment for age and gender.

Phenotype	Best interaction model	Testing accuracy (%)	P value
(1) Two-way interaction models			
MetS	*RORB* rs972902, smoking	54.33	**< 0.001**
High waist circumference^a^	*ARNTL* rs10832020, physical activity	51.93	0.079
High triglyceride^b^	*RORB* rs972902, smoking	52.17	0.068
Low HDL^c^	*RORB* rs972902, physical activity	55.45	**< 0.001**
High blood pressure^d^	*RORA* rs8034880, physical activity	52.75	0.038
High fasting glucose^e^	*PER3* rs10746473, alcohol consumption	53.57	0.003
(2) Three-way interaction models			
MetS	*PER3* rs10746473, *RORB* rs972902, smoking	55.73	**0.001**
High waist circumference^a^	*ARNTL* rs10832020, *PER3* rs10746473, physical activity	52.08	0.063
High triglyceride^b^	*ARNTL* rs10832020, *PER3* rs10746473, physical activity	53.42	0.016
Low HDL^c^	*PER3* rs10746473, smoking, physical activity	56.11	**< 0.001**
High blood pressure^d^	*PER3* rs10746473, *RORA* rs8034880, physical activity	51.02	0.280
High fasting glucose^e^	*ARNTL* rs10832020, *GSK3B* rs2199503, physical activity	54.45	**0.001**
(3) Four-way interaction models			
MetS	*PER3* rs10746473, *RORA* rs8034880, *RORB* rs972902, smoking	54.23	0.008
High waist circumference^a^	*ARNTL* rs10832020, *GSK3B* rs2199503, *PER3* rs10746473, physical activity	52.30	0.051
High triglyceride^b^	*ARNTL* rs10832020, *PER3* rs10746473, *RORA* rs8034880, physical activity	51.67	0.168
Low HDL^c^	*GSK3B* rs2199503, *PER3* rs10746473, *RORB* rs972902, physical activity	55.89	**< 0.001**
High blood pressure^d^	*ARNTL* rs10832020, *PER3* rs10746473, *RORA* rs8034880, physical activity	51.28	0.221
High fasting glucose^e^	*ARNTL* rs10832020, *GSK3B* rs2199503, *PER3* rs10746473, physical activity	54.50	**0.002**

GMDR = generalized multifactor dimensionality reduction, HDL = high-density lipoprotein cholesterol, MetS = metabolic syndrome. P value was based on 1,000 permutations. Analysis was obtained after adjustment for covariates including age and gender. P values of < 0.0028 (Bonferroni correction: 0.05/18) are shown in bold.

^a^ Waist circumference ≥ 90 cm in male subjects, waist circumference ≥ 80 cm in female subjects.

^b^ Triglyceride ≥ 150 mg/dl.

^c^ HDL< 40 mg/dl in male subjects, HDL < 50 mg/dl in female subjects.

^d^ Systolic blood pressure ≥ 130 mmHg or diastolic blood pressure ≥ 85 mmHg.

^e^ Fasting glucose ≥ 100 mg/dl.

Finally, statistical power analysis revealed that the present study had a 99.9% power to detect associations of *ARNTL* rs10832020, *GSK3B* rs2199503, *PER3* rs10746473, *RORA* rs8034880, or *RORB* rs972902 with MetS among the MetS and non-MetS subjects.

## Discussion

Our association study is the first to date to examine whether the main effects of 881 SNPs in 29 circadian clock-related genes are significantly associated with the risk of MetS and its individual components independently and/or through gene-gene interactions among Taiwanese individuals. We also investigated the relationship between these genes and health-related behaviors to examine whether these genes confer a risk of MetS according to its effect on gene-environment interactions. A growing body of evidence indicates that genetic association studies using individual genes may overlook the associations that can only be identified if gene-gene and gene-environment interactions are explored [34]. Therefore, we further identified five key SNPs in five circadian clock genes such as *ARNTL*, *GSK3B*, *PER3*, *RORA*, and *RORB* to assess gene-gene and gene-environment interactions although the association with MetS did not remain significant after performing Bonferroni correction (P < 6 x 10^−5^). Here, we report for the first time that gene-gene interactions of the *ARNTL* rs10832020, *GSK3B* rs2199503, *PER3* rs10746473, *RORA* rs8034880, and *RORB* rs972902 SNPs may contribute to the etiology of MetS. Moreover, there were gene-environment interactions between *ARNTL* rs10832020, *GSK3B* rs2199503, *PER3* rs10746473, and *RORB* rs972902 with health-related behaviors, such as alcohol consumption, smoking status, and physical activity. Finally, our data revealed that the *GSK3B* rs2199503 SNP was associated with the individual component of MetS such as high fasting glucose.

Regarding the *ARNTL* gene, we found a positive association of MetS with 4 SNPs in the *ARNTL* gene, especially the rs10832020 SNP. The *ARNTL* gene is located on chromosome 11p15 and encodes a basic helix-loop-helix protein to build the ARNTL/CLOCK heterodimeric protein with CLOCK, another basic helix-loop-helix protein [9]. The *ARNTL* gene has been associated with metabolic regulation [9, 13]. Woon et al. identified a three-marker haplotype (rs6486121, rs3789327, and rs969485) of the *ARNTL* gene as an indicator of hypertension [9]. Moreover, Shimba et al. demonstrated that deletion of the *Arntl* gene induced MetS and dyslipidemia in an animal model [13]. In the present study, we detected an association of high blood pressure with *ARNTL* rs3789327 (P = 0.0346), but not with *ARNTL* rs6486121 and rs969485 (data not shown). In addition, we did not detect an association of MetS with *ARNTL* rs6486121, rs3789327, and rs969485 in this study.

Further, five potential candidate SNPs for MetS were identified at the *GSK3B* gene, particularly the rs2199503 SNP. We also suggested an association of *GSK3B* rs2199503 with high fasting glucose. The *GSK3B* gene is located on chromosome 3q13.3 and encodes a subfamily of glycogen synthase kinase (GSK), which comprises two isoforms GSK3A and GSK3B. It has been implicated that GSK3 regulates numerous cellular functions such as metabolism, circadian rhythms, neurological degeneration, and immune responses [35]. Chen et al. reported that the expression of phosphatidylinositol-3 kinase (PI3K)-dependent GSK3 stimulates the production of adiponectin and protects against diet-induced MetS in an animal study [36].

On another note, there was an association of MetS with 6 SNPs within the *PER3* gene, notably the rs10746473 SNP. Our analysis also indicated that there was an association of *PER3* rs10746473 with high waist circumference. The *PER3* gene, located on chromosome 1p36.23, encodes components of the circadian rhythms of locomotor activity, metabolism, and feeding behavior [37]. Dallmann and Weaver reported that the *Per3* mutant mice led to higher body mass on a high fat diet, suggesting *Per3* may play a role in body mass regulation [38]. In addition, Costa et al. demonstrated that *Per3* knock-out mice displayed glucose intolerance as well as altered body composition (such as increased adipose and decreased muscle), indicating *Per3* as potent mediator of cell fate [14]. In an animal study, Pendergast et al. also showed that the function of *Per3* was tissue specific such that the loss of functional PER3 had an effect on circadian period in about half of the analyzed tissues, demonstrating *Per3* as an important player in the mammalian circadian system [39].

Moreover, our results are the first to raise the possibility that 43 SNPs in the *RORA* gene and 6 SNPs in the *RORB* gene may contribute to the susceptibility for MetS, especially *RORA* (rs17237367, rs58469372, rs12591650, rs12594188, rs17270446, rs11630062, rs8029848, rs8034880, rs72752802) and *RORB* rs972902. Our results also suggested an association of high fasting glucose with the *RORA* rs8029848, *RORA* rs8034880, and *RORB* rs972902 SNPs; however, the significance did not persist after adjusting for Bonferroni correction. The proteins encoded by *RORA* (located on chromosome 15q22.2) and *RORB* (located on chromosome 9q2) constitute a subfamily of nuclear hormone receptors [40]. The *RORA* and *RORB* genes have been implicated in the regulation of a wide variety of physiological processes, including metabolism, circadian rhythm, and inflammatory responses [40, 41]. Kang et al. demonstrated that *Rora*-deficient mice showed a markedly reduced susceptibility to age- and diet-induced MetS, implicating *Rora* plays a critical role in the regulation of several aspects of MetS [41].

Previously, circadian clock genes have been reported to associate with MetS, such as a three-marker haplotype (rs4864548, rs3736544, and rs1801260) of the *CLOCK* gene [7] and *CRY2* rs75065406 [8]. In addition, circadian clock genes have been shown to link with the individual components of MetS, including an association of *NPAS2* rs11541353 with hypertension [10], an association of *PER2* #10870 with high fasting glucose [10], and an association of *CRY1* haplotype (rs4964513 and rs12821586) with elevated blood pressure [8]. In this study, we did not weigh *CLOCK* (rs4864548, rs3736544, and rs1801260), *CRY1* (rs4964513 and rs12821586), *CRY2* rs75065406, *NPAS2* rs11541353, and *PER2* #10870 due to lack of these SNPs in the custom chip. It is worth mentioning that the minor allele frequencies of *CRY2* rs75065406 and *NPAS2* rs11541353 are all 0% in Asian populations (such as Japanese and Han Chinese) as shown in the public data from the 1000 Genomes Project (http://www.1000genomes.org). Moreover, it should be noted that *CRY1* (rs4964513 and rs12821586) are annotated within the mitochondrial transcription termination factor 2 (*MTERF2*) gene, which is in the vicinity of the *CRY1* gene, as shown in the dbSNP database (https://www.ncbi.nlm.nih.gov/). On the other hand, we identified other potential candidate SNPs for MetS, including 3 SNPs in the *CRY1* gene and 6 SNPs in the *NPAS2* gene.

Intriguingly, another finding was that we further inferred the epistatic effects between *ARNTL*, *GSK3B*, *PER3*, *RORA*, and *RORB* in influencing MetS by using the GMDR approach. To our knowledge, no other study has been conducted to evaluate gene-gene interactions between these genes. Besides the statistical significance, the potential biological mechanism under the interaction models was our concern. The functional relevance of the interactive effects of *ARNTL*, *GSK3B*, *PER3*, *RORA*, and *RORB* on MetS remains to be elucidated. In mammals, the molecular clock mechanism is currently viewed as a complex interplay of transcriptional feedback regulatory loops involving various circadian clock genes to generate and maintain circadian rhythms [40]. The core circadian clock gene *ARNTL* encodes the ARNTL protein, which is a basic helix-loop-helix protein that forms heterodimers with either CLOCK or NPAS2, two other basic helix-loop-helix proteins [2]. The ARNTL/CLOCK heterodimer initiates the transcription of various target genes such as *PER1*, *PER2*, *PER3*, *CRY1*, and *CRY2* via E-box elements in the promoters of target genes [2]. In turn, the resulting PER and CRY proteins inhibit further ARNTL/CLOCK transcriptional activity, thereby allowing a new cycle to start from a level of low transcriptional activity. In order to improve the robustness of the previous loop, an additional feedback loop involves the nuclear receptors NR1D1 (or Rev-erb alpha)and RORA, which regulate the circadian transcription of the *ARNTL* gene and provide a good timing of the core clock machinery [40].

Remarkably, the GMDR analysis of gene-environment interactions reflected the interplay among *PER3*, *RORB*, and smoking in influencing MetS. Similarly, we tracked down several significant gene-environment interaction models in influencing individual components such as low HDL and high fasting glucose. It has been highlighted that common diseases are known to have a considerable genetic contribution, although only a proportion of complex diseases overall is explained by the established candidate genes, pointing out that environmental effects and gene-environment interactions will be our key concern in the outlook for genomic studies [42].

In terms of the average ages in the MetS and non-MetS groups, subjects with MetS were significantly older than those without MetS in this study (Table 1). Consistent with our results, Hwang et al. reported that the prevalence of MetS in Taiwan increased with age from 5.2% in individuals aged 20–29 to 36.5% in individuals aged 70–79, reaching peak levels in the 7th decade of life for both men and women [43]. Similarly, the prevalence of the MetS increased with age (ranging from about 7% in persons aged 20–29, about 14% in persons aged 30–39, about 20% in persons aged 40–49, to about 44% in persons aged 60–69) in National Health and Nutrition Examination Survey (1988–1994) in the United States [44].

This study has both strengths and limitations. The main weakness was that our observations require much further research to validate whether the findings are replicated in diverse ethnic groups [45–47]. This study may also fail to assess other relevant circadian clock genes such as the basic helix-loop-helix family member e41 (*BHLHE41*) gene due to lack of those genes in the custom chip. In future work, prospective clinical trials with other ethnic populations are needed to examine a thorough evaluation of the association and interactions of the investigated genes with MetS and its individual components [48, 49]. On the other hand, a major strength of our study was that we employed health-related behavior data, which served an exclusionary opportunity to facilitate the interplay between the investigated genes and environmental factors.

## Conclusions

In conclusion, we conducted an extensive analysis of the association as well as gene-gene and gene-environment interactions of the circadian clock genes with MetS and its individual components in Taiwanese subjects. Our findings indicate that the *ARNTL*, *GSK3B*, *PER3*, *RORA*, and *RORB* genes may affect the prevalence of MetS independently and/or through complex gene-gene and gene-environment interactions. Furthermore, the circadian clock genes may be a determinant of MetS component factors. Independent replication studies with larger sample sizes will likely demonstrate further insights into the role of the circadian clock genes found in this study.

## Supporting information

S1 Table Odds ratio analysis after adjustment for covariates between the MetS and 881 SNPs in 29 circadian clock genes. (DOC)

S2 Table Odds ratio analysis with odds ratios after adjustment for covariates (including age and gender) between the MetS and 13 selective circadian clock genes, which have an evidence of association (P < 0.05). (DOC)

S3 Table Q values and FDRs for odds ratio analysis between the MetS and 881 SNPs in 29 circadian clock genes. (DOC)

S4 Table Q values and FDRs for odds ratio analysis between individual components of the MetS and five key SNPs in the five circadian clock genes (including ARNTL rs10832020, GSK3B rs2199503, PER3 rs10746473, RORA rs8034880, and RORB rs972902). (DOC)

S5 Table Q values and FDRs for two-way gene-gene interaction models by using the GMDR method. (DOC)

S6 Table Gene-gene interaction models identified by the GMDR method with adjustment for age and gender. (DOC)

S7 Table Q values and FDRs for gene-environment interaction models identified by the GMDR method. (DOC)
