# Supplementary material for: Effects of circadian clock genes and health-related behavior on metabolic syndrome in a Taiwanese population: Evidence from association and interaction analysis
Source: PLoS One. 2017 Mar 15;12(3):e0173861. doi: 10.1371/journal.pone.0173861 (PMC5352001; doi:10.1371/journal.pone.0173861)
Supplement: S1 Table — (DOC) [file pone.0173861.s001.doc]

**S1 Table.** Odds ratio analysis after adjustment for covariates between the MetS and 881 SNPs in 29 circadian clock genes.

| Gene | CHR | SNP | Alleles | P (Additive) | P (Dominant) | P (Recessive) |
| --- | --- | --- | --- | --- | --- | --- |
| *ADCYAP1* | 18 | rs1893154 | A/G | 0.5041 | 0.9410 | 0.5018 |
|  |  | rs8086678 | T/C | 0.4128 | 0.8820 | 0.3884 |
|  |  | rs928980 | T/C | 0.3854 | 0.2186 | 0.6832 |
|  |  | rs2231187 | G/A | 0.6971 | 0.8740 | 0.6985 |
|  |  | rs1610037 | G/A | 0.6395 | 0.5795 | 0.6953 |
| *ARNTL* | 11 | rs1481892 | G/C | 0.3611 | 0.0356 | 0.7525 |
|  |  | rs7950226 | G/A | 0.7620 | 0.6107 | 0.9172 |
|  |  | rs7951393 | T/C | 0.5352 | 0.0988 | 0.5871 |
|  |  | rs72867496 | G/A | 0.4335 | 0.3571 | 0.4917 |
|  |  | rs10832020 | C/T | 0.0114 | 0.5586 | 0.0065 |
|  |  | rs6486120 | T/G | 0.4937 | 0.6181 | 0.0990 |
|  |  | rs11022761 | T/C | 0.2785 | 0.3859 | 0.3024 |
|  |  | rs4757143 | C/T | 0.1736 | 0.3076 | 0.1945 |
|  |  | rs10741616 | G/A | 0.2244 | 0.1296 | 0.5086 |
|  |  | rs11022762 | C/T | 0.4805 | 0.4417 | 0.6270 |
|  |  | rs10766076 | A/T | 0.9044 | 0.5346 | 0.4898 |
|  |  | rs12805304 | A/T | 0.1211 | 0.7781 | 0.0574 |
|  |  | rs9633835 | G/A | 0.1139 | 0.0338 | 0.4712 |
|  |  | rs34188368 | T/C | 0.3718 | 0.7850 | 0.3377 |
|  |  | rs28711392 | T/C | 0.9096 | 0.1771 | 0.5469 |
|  |  | rs10766077 | G/A | 0.9400 | 0.2273 | 0.5618 |
|  |  | rs7924734 | G/A | 0.3534 | 0.8648 | 0.1400 |
|  |  | rs11022769 | C/A | 0.4391 | 0.8161 | 0.1938 |
|  |  | rs16912743 | G/A | 0.1915 | 0.4877 | 0.2048 |
|  |  | rs6486121 | T/C | 0.8970 | 0.4009 | 0.9511 |
|  |  | rs7947951 | G/A | 0.8861 | 0.9683 | 0.8729 |
|  |  | rs16912751 | C/T | 0.4592 | 0.4142 | 0.5275 |
|  |  | rs1026071 | G/A | 0.6450 | 0.2946 | 0.8335 |
|  |  | rs34834014 | C/T | 0.7731 | 0.5200 | 0.7955 |
|  |  | rs11022775 | T/C | 0.5781 | 0.5039 | 0.5917 |
|  |  | rs34991502 | G/T | 0.2741 | 0.8830 | 0.2593 |
|  |  | rs1868049 | C/T | 0.1965 | 0.4878 | 0.1886 |
|  |  | rs3789327 | G/A | 0.3861 | 0.8293 | 0.2957 |
|  |  | rs11022778 | G/T | 0.9750 | 0.7029 | 0.9984 |
|  |  | rs4757151 | G/A | 0.8953 | 0.7756 | 0.9739 |
|  |  | rs75854041 | C/T | 0.0252 | 0.9724 | 0.0234 |
|  |  | rs74762146 | A/G | 0.5809 | 0.4362 | 0.6314 |
|  |  | rs72869173 | A/G | 0.2132 | 0.8352 | 0.2126 |
|  |  | rs11022779 | A/G | 0.7601 | 0.5074 | 0.7118 |
|  |  | rs969485 | A/G | 0.3168 | 0.6975 | 0.2828 |
| *ARNTL2* | 12 | rs7137588 | C/G | 0.3356 | 0.1749 | 0.3476 |
|  |  | rs4964052 | T/G | 0.2202 | 0.5202 | 0.2041 |
|  |  | rs16931885 | A/G | 0.8835 | 0.2520 | 0.7467 |
|  |  | rs17497606 | T/C | 0.4541 | 0.2754 | 0.4651 |
|  |  | rs16931888 | A/G | 0.9239 | 0.2402 | 0.6896 |
|  |  | rs61915952 | T/C | 0.8640 | 0.2722 | 0.7698 |
|  |  | rs11048977 | G/A | 0.9263 | 0.6074 | 0.7691 |
|  |  | rs1037924 | G/A | 0.5710 | 0.9565 | 0.5392 |
|  |  | rs17497857 | C/T | 0.9984 | 0.2235 | 0.9984 |
|  |  | rs2968756 | A/G | 0.8741 | 0.4025 | 0.8634 |
|  |  | rs10771339 | C/G | 0.7685 | 0.5122 | 0.4768 |
|  |  | rs2927030 | T/C | 0.7472 | 0.2095 | 0.9719 |
|  |  | rs3751220 | G/A | 0.6994 | 0.1024 | 0.5936 |
|  |  | rs4964059 | C/A | 0.4545 | 0.1805 | 0.9203 |
|  |  | rs11048994 | A/G | 0.4595 | 0.3686 | 0.5677 |
|  |  | rs11048995 | A/G | 0.1737 | 0.2672 | 0.2440 |
|  |  | rs4964060 | G/A | 0.2294 | 0.4263 | 0.2681 |
|  |  | rs12300289 | G/A | 0.2541 | 0.1601 | 0.3007 |
|  |  | rs3751221 | C/A | 0.1628 | 0.1537 | 0.1959 |
|  |  | rs16931937 | C/T | 0.3602 | 0.1590 | 0.4022 |
|  |  | rs16931939 | T/C | 0.1999 | 0.1523 | 0.2265 |
|  |  | rs4931075 | A/G | 0.1493 | 0.0260 | 0.6396 |
|  |  | rs813535 | G/A | 0.6690 | 0.2137 | 0.5504 |
|  |  | rs1256955 | G/T | 0.1159 | 0.0366 | 0.4816 |
|  |  | rs10842913 | A/G | 0.2896 | 0.2119 | 0.3179 |
|  |  | rs2682706 | C/T | 0.1123 | 0.0261 | 0.5453 |
|  |  | rs73079936 | A/G | 0.3354 | 0.0934 | 0.3830 |
|  |  | rs11049010 | C/A | 0.1160 | 0.1555 | 0.2059 |
|  |  | rs1443859 | C/T | 0.3514 | 0.5601 | 0.3721 |
|  |  | rs683813 | C/T | 0.4207 | 0.2166 | 0.5642 |
| *BHLHE40* | 3 | rs58358486 | C/A | 0.7672 | 0.7831 | 0.5476 |
|  |  | rs6808127 | A/C | 0.2611 | 0.9766 | 0.0975 |
|  |  | rs6775761 | A/G | 0.6432 | 0.7066 | 0.3644 |
|  |  | rs9844696 | T/C | 0.0389 | 0.3454 | 0.0390 |
|  |  | rs9875607 | A/T | 0.5967 | 0.2656 | 0.8831 |
|  |  | rs728390 | T/C | 0.5235 | 0.8235 | 0.4218 |
|  |  | rs728389 | T/C | 0.4373 | 0.8591 | 0.3426 |
|  |  | rs17041730 | A/G | 0.5956 | 0.3252 | 0.6078 |
|  |  | rs17041733 | C/T | 0.8799 | 0.7073 | 0.9996 |
|  |  | rs1514451 | A/G | 0.9278 | 0.1756 | 0.3611 |
|  |  | rs59218436 | T/G | 0.9827 | 0.1753 | 0.6014 |
|  |  | rs13069852 | C/A | 0.4577 | 0.5825 | 0.4833 |
|  |  | rs79878432 | G/A | 0.7983 | 0.2805 | 0.8153 |
|  |  | rs4402927 | A/C | 0.8990 | 0.4796 | 0.7265 |
|  |  | rs7629118 | C/A | 0.6455 | 0.7374 | 0.6630 |
|  |  | rs9990146 | A/T | 0.0661 | 0.0209 | 0.3919 |
|  |  | rs9990172 | A/G | 0.3648 | 0.6905 | 0.3727 |
|  |  | rs6442920 | A/G | 0.5653 | 0.5850 | 0.5941 |
|  |  | rs6764243 | A/G | 0.3340 | 0.7000 | 0.3397 |
|  |  | rs75888088 | C/T | 0.1656 | 0.6912 | 0.1640 |
|  |  | rs9311438 | G/T | 0.4054 | 0.5862 | 0.4235 |
|  |  | rs79740996 | A/C | 0.7936 | 0.4840 | 0.8321 |
|  |  | rs9836120 | G/A | 0.2246 | 0.1170 | 0.4967 |
|  |  | rs72997163 | C/T | 0.5501 | 0.6433 | 0.6096 |
|  |  | rs13065754 | T/G | 0.4991 | 0.9820 | 0.3204 |
|  |  | rs7635516 | T/C | 0.1711 | 0.5675 | 0.1681 |
|  |  | rs7638383 | A/G | 0.9965 | 0.5601 | 0.9965 |
|  |  | rs63538861 | C/T | 0.7952 | 0.2552 | 0.5762 |
|  |  | rs3846155 | T/C | 0.8420 | 0.2624 | 0.6267 |
|  |  | rs7640124 | A/C | 0.8666 | 0.7331 | 0.8178 |
|  |  | rs6442925 | T/C | 0.4646 | 0.5070 | 0.4574 |
|  |  | rs1588794 | C/T | 0.7702 | 0.1084 | 0.9974 |
|  |  | rs6764396 | C/G | 0.9543 | 0.5857 | 0.8182 |
|  |  | rs1110261 | A/G | 0.8533 | 0.8242 | 0.8723 |
|  |  | rs1104976 | C/A | 0.8145 | 0.4119 | 0.7017 |
| *CLOCK* | 4 | rs3749473 | T/C | 0.9819 | 0.5963 | 0.9622 |
|  |  | rs6832769 | G/A | 0.9475 | 0.7404 | 0.9614 |
|  |  | rs11932595 | G/A | 0.9778 | 0.7650 | 0.9703 |
|  |  | rs12642716 | G/A | 0.7446 | 0.9261 | 0.7100 |
|  |  | rs62303728 | A/G | 0.7889 | 0.8905 | 0.7907 |
|  |  | rs11133391 | T/C | 0.8083 | 0.8239 | 0.6551 |
|  |  | rs7673908 | G/A | 0.9673 | 0.8905 | 0.9198 |
|  |  | rs117876670 | G/A | NA | 0.0947 | NA |
| *CRY1* | 12 | rs7303842 | A/G | 0.0357 | 0.0261 | 0.0701 |
|  |  | rs79487478 | A/G | 0.9048 | 0.0374 | 0.7603 |
|  |  | rs11113179 | T/C | 0.2827 | 0.8099 | 0.2697 |
|  |  | rs17038985 | A/G | 0.0328 | 0.0251 | 0.0652 |
| *CRY2* | 11 | rs10838524 | G/A | 0.1103 | 0.9708 | 0.0792 |
|  |  | rs11605924 | C/A | 0.0896 | 0.9878 | 0.0642 |
|  |  | rs4756034 | G/A | 0.0962 | 0.2336 | 0.1362 |
|  |  | rs4756035 | T/C | 0.0997 | 0.2296 | 0.1453 |
|  |  | rs2292912 | G/C | 0.0796 | 0.2094 | 0.1163 |
|  |  | rs11038699 | G/A | 0.2067 | 0.1977 | 0.2228 |
|  |  | rs2292910 | C/A | 0.2736 | 0.6274 | 0.2723 |
| *CSNK1D* | 17 | rs116950947 | A/G | 0.6531 | 0.5147 | 0.6435 |
|  |  | rs3829773 | T/C | 0.9598 | 0.7237 | 0.9315 |
|  |  | rs4789846 | T/C | 0.4126 | 0.3058 | 0.5511 |
| *CSNK1E* | 22 | rs5750581 | C/T | 0.2956 | 0.9707 | 0.2932 |
|  |  | rs135763 | A/C | 0.3643 | 0.9444 | 0.3599 |
|  |  | rs1997644 | A/G | 0.1527 | 0.6134 | 0.1246 |
|  |  | rs3747169 | A/G | 0.7335 | 0.1615 | 0.8627 |
|  |  | rs195314 | C/G | 0.1604 | 0.3586 | 0.0928 |
|  |  | rs17753544 | A/G | 0.6452 | 0.1557 | 0.7314 |
|  |  | rs55719829 | C/T | 0.8722 | 0.1532 | 0.9578 |
|  |  | rs196105 | G/A | 0.6827 | 0.0740 | 0.8388 |
|  |  | rs138366 | A/G | 0.2660 | 0.4189 | 0.1756 |
|  |  | rs138369 | G/A | 0.3304 | 0.4083 | 0.4290 |
| *GSK3B* | 3 | rs3732361 | G/A | 0.1723 | 0.4377 | 0.0228 |
|  |  | rs2873950 | A/C | 0.4285 | 0.8982 | 0.1839 |
|  |  | rs10934500 | C/T | 0.3401 | 0.1078 | 0.9348 |
|  |  | rs1719888 | C/T | 0.9380 | 0.5996 | 0.9191 |
|  |  | rs45567135 | G/A | 0.3171 | 0.0844 | 0.2948 |
|  |  | rs16830594 | G/A | 0.5974 | 0.2461 | 0.2566 |
|  |  | rs6795653 | T/C | 0.4155 | 0.4634 | 0.1287 |
|  |  | rs6782799 | C/T | 0.2347 | 0.4911 | 0.0469 |
|  |  | rs79646574 | G/A | 0.8800 | 0.5489 | 0.8548 |
|  |  | rs4340737 | T/C | 0.3589 | 0.1452 | 0.9681 |
|  |  | rs78268305 | A/G | 0.7792 | 0.5387 | 0.7572 |
|  |  | rs13314595 | T/C | 0.5010 | 0.4789 | 0.4762 |
|  |  | rs7431209 | A/G | 0.0268 | 0.7462 | 0.0055 |
|  |  | rs9878473 | T/C | 0.1950 | 0.4452 | 0.0320 |
|  |  | rs76950779 | C/T | 0.7811 | 0.0711 | 0.7987 |
|  |  | rs6807868 | T/C | 0.7771 | 0.6261 | 0.7591 |
|  |  | rs12054090 | C/T | 0.4676 | 0.1682 | 0.8309 |
|  |  | rs12638973 | T/C | 0.3264 | 0.1260 | 0.9540 |
|  |  | rs76330913 | C/T | 0.7768 | 0.5587 | 0.7557 |
|  |  | rs75008340 | T/C | 0.9987 | 0.3853 | 0.9987 |
|  |  | rs10934506 | C/T | 0.4455 | 0.2054 | 0.9746 |
|  |  | rs968824 | G/A | 0.7753 | 0.5614 | 0.7544 |
|  |  | rs75126237 | T/C | 0.3281 | 0.0254 | 0.2948 |
|  |  | rs2199503 | T/C | 0.0331 | 0.7245 | 0.0070 |
|  |  | rs334535 | T/C | 0.5722 | 0.4077 | 0.5424 |
|  |  | rs334559 | A/G | 0.6020 | 0.4779 | 0.5755 |
| [*HCRTR2*](mailto:HCRTR@) | 6 | rs10456181 | A/G | 0.4137 | 0.2776 | 0.4612 |
|  |  | rs4364482 | A/G | 0.0876 | 0.2999 | 0.0761 |
|  |  | rs6927478 | G/T | 0.8249 | 0.9944 | 0.8136 |
|  |  | rs3134689 | G/A | 0.6100 | 0.9984 | 0.5799 |
|  |  | rs3122152 | T/C | 0.8464 | 0.8475 | 0.8671 |
|  |  | rs4712099 | A/G | 0.6424 | 0.0485 | 0.9801 |
|  |  | rs9475195 | C/T | 0.1925 | 0.4622 | 0.1903 |
|  |  | rs12526414 | T/C | 0.6105 | 0.9081 | 0.5648 |
|  |  | rs3134704 | G/A | 0.1713 | 0.6597 | 0.1086 |
|  |  | rs3134705 | G/A | 0.1911 | 0.4508 | 0.1861 |
|  |  | rs9370402 | T/C | NA | 0.6123 | NA |
|  |  | rs3122155 | C/G | 0.1561 | 0.3464 | 0.1806 |
|  |  | rs3122156 | G/T | 0.3190 | 0.5278 | 0.2336 |
|  |  | rs3122160 | T/G | 0.4716 | 0.5678 | 0.4097 |
|  |  | rs3122162 | G/T | 0.2419 | 0.2665 | 0.1044 |
|  |  | rs60748030 | T/A | 0.6386 | 0.0828 | 0.3808 |
|  |  | rs3134711 | A/G | 0.0789 | 0.3191 | 0.0396 |
|  |  | rs7741664 | C/T | 0.0659 | 0.7928 | 0.0521 |
|  |  | rs12525016 | G/A | 0.3900 | 0.4904 | 0.3613 |
|  |  | rs3122167 | C/T | 0.1334 | 0.3477 | 0.0521 |
|  |  | rs9396073 | T/C | 0.1370 | 0.7930 | 0.1158 |
|  |  | rs2811239 | A/G | 0.2629 | 0.7059 | 0.0518 |
|  |  | rs74937200 | A/G | 0.6982 | 0.4269 | 0.7059 |
|  |  | rs2653342 | A/G | 0.9677 | 0.8975 | 0.9654 |
|  |  | rs2653344 | T/C | 0.5642 | 0.8909 | 0.5617 |
|  |  | rs7452651 | A/G | 0.6258 | 0.7977 | 0.6337 |
|  |  | rs2653346 | A/G | 0.6310 | 0.8137 | 0.5843 |
|  |  | rs2811241 | T/C | 0.6241 | 0.6621 | 0.4428 |
|  |  | rs2653350 | G/A | 0.8909 | 0.6021 | 0.8792 |
|  |  | rs74296544 | A/G | 0.4739 | 0.5403 | 0.4618 |
|  |  | rs3800543 | A/G | 0.9526 | 0.9559 | 0.9598 |
|  |  | rs76380807 | G/A | 0.8328 | 0.8043 | 0.7725 |
| *KLF10* | 8 | rs3191333 | A/G | 0.8504 | 0.9132 | 0.8550 |
|  |  | rs11552577 | G/T | 0.8029 | 0.6794 | 0.7611 |
| *NFIL3* | 9 | rs80064239 | C/T | 0.5118 | 0.1131 | 0.4831 |
|  |  | rs7021746 | A/G | 0.7971 | 0.7123 | 0.8587 |
|  |  | rs10991925 | C/T | 0.2626 | 0.2855 | 0.3137 |
|  |  | rs2440589 | T/C | 0.2015 | 0.2818 | 0.3028 |
|  |  | rs2482357 | G/A | 0.7823 | 0.8033 | 0.5665 |
|  |  | rs2482704 | T/G | 0.2090 | 0.2727 | 0.3256 |
|  |  | rs2482359 | G/A | 0.4753 | 0.1610 | 0.5354 |
|  |  | rs10820852 | A/C | 0.3815 | 0.2781 | 0.4579 |
|  |  | rs13297268 | A/G | 0.3329 | 0.1312 | 0.3457 |
|  |  | rs10116162 | C/T | 0.8232 | 0.6373 | 0.9138 |
| *NPAS2* | 2 | rs13390078 | A/G | 0.7734 | 0.3869 | 0.9160 |
|  |  | rs6542992 | T/G | 0.4676 | 0.5223 | 0.5824 |
|  |  | rs75656425 | C/T | 0.1302 | 0.5916 | 0.1109 |
|  |  | rs77568479 | C/T | 0.3608 | 0.8227 | 0.2977 |
|  |  | rs78801659 | A/G | 0.1555 | 0.8636 | 0.1527 |
|  |  | rs3896080 | T/C | 0.1746 | 0.6599 | 0.1314 |
|  |  | rs79218385 | C/T | 0.8472 | 0.2801 | 0.9101 |
|  |  | rs3860455 | T/G | 0.0550 | 0.2462 | 0.0679 |
|  |  | rs57365275 | A/G | 0.2119 | 0.3624 | 0.2613 |
|  |  | rs72627416 | A/G | 0.1631 | 0.3129 | 0.2087 |
|  |  | rs6542994 | A/G | 0.7926 | 0.7830 | 0.8577 |
|  |  | rs17699370 | A/C | 0.9655 | 0.2779 | 0.8506 |
|  |  | rs55982284 | G/A | 0.9555 | 0.6162 | 0.9639 |
|  |  | rs62156093 | G/A | 0.5875 | 0.6342 | 0.5777 |
|  |  | rs13012930 | A/G | 0.3836 | 0.6787 | 0.3749 |
|  |  | rs4611661 | C/T | 0.2841 | 0.7282 | 0.2832 |
|  |  | rs4377354 | G/A | 0.2326 | 0.1278 | 0.3352 |
|  |  | rs72816926 | C/T | 0.0928 | 0.9831 | 0.0903 |
|  |  | rs11691732 | C/G | 0.2575 | 0.8637 | 0.2442 |
|  |  | rs7582455 | T/C | 0.0765 | 0.6188 | 0.0749 |
|  |  | rs2309992 | A/G | 0.6102 | 0.3909 | 0.7061 |
|  |  | rs7598826 | A/G | 0.9497 | 0.5922 | 0.6947 |
|  |  | rs59005495 | T/C | 0.3854 | 0.7675 | 0.3615 |
|  |  | rs75803056 | T/C | 0.8958 | 0.0953 | 0.7674 |
|  |  | rs76376883 | A/G | 0.9970 | 0.9843 | 0.9970 |
|  |  | rs1811399 | C/A | 0.9451 | 0.6441 | 0.9342 |
|  |  | rs983287 | G/A | 0.2257 | 0.1865 | 0.2967 |
|  |  | rs2043534 | T/C | 0.5134 | 0.9534 | 0.4947 |
|  |  | rs6759386 | T/G | 0.2111 | 0.1090 | 0.2998 |
|  |  | rs930309 | T/A | 0.9605 | 0.6616 | 0.8049 |
|  |  | rs12472321 | T/C | 0.5404 | 0.5714 | 0.6335 |
|  |  | rs12476292 | A/G | 0.6170 | 0.2023 | 0.5195 |
|  |  | rs17024926 | T/C | 0.1166 | 0.6183 | 0.0768 |
|  |  | rs72627426 | G/A | 0.2331 | 0.9013 | 0.1623 |
|  |  | rs72627427 | T/C | 0.2243 | 0.9871 | 0.1357 |
|  |  | rs12712084 | T/C | 0.3687 | 0.0525 | 0.7223 |
|  |  | rs1369481 | T/C | 0.3271 | 0.0804 | 0.4901 |
|  |  | rs17654772 | A/G | 0.5209 | 0.6695 | 0.5112 |
|  |  | rs920086 | A/G | 0.3141 | 0.1105 | 0.7450 |
|  |  | rs11123853 | A/G | 0.6109 | 0.6173 | 0.7185 |
|  |  | rs34333438 | G/A | 0.8192 | 0.5714 | 0.8317 |
|  |  | rs72627430 | T/C | 0.9048 | 0.0120 | 0.9848 |
|  |  | rs7570190 | G/T | 0.9262 | 0.4598 | 0.9068 |
|  |  | rs12989454 | T/C | 0.8519 | 0.5838 | 0.8584 |
|  |  | rs13418893 | A/G | 0.3785 | 0.2637 | 0.6471 |
|  |  | rs11894322 | C/A | 0.8572 | 0.8471 | 0.8088 |
|  |  | rs11894370 | G/A | 0.8279 | 0.9665 | 0.8044 |
|  |  | rs356643 | G/A | 0.4780 | 0.8219 | 0.3621 |
|  |  | rs77985008 | T/C | 0.0482 | 0.4466 | 0.0361 |
|  |  | rs7602455 | T/C | 0.1576 | 0.8567 | 0.1341 |
|  |  | rs17655330 | A/C | 0.4223 | 0.2810 | 0.3981 |
|  |  | rs6725296 | A/G | 0.0712 | 0.9521 | 0.0654 |
|  |  | rs79320559 | A/G | 0.0808 | 0.4418 | 0.0617 |
|  |  | rs356652 | G/T | 0.4410 | 0.8553 | 0.3959 |
|  |  | rs3754674 | C/G | 0.9694 | 0.3638 | 0.8225 |
|  |  | rs3754675 | C/T | 0.1052 | 0.2420 | 0.1443 |
|  |  | rs3820786 | A/G | 0.4395 | 0.3840 | 0.4216 |
|  |  | rs13025524 | A/G | 0.8780 | 0.4348 | 0.9898 |
|  |  | rs3768984 | C/A | 0.3615 | 0.3047 | 0.5051 |
|  |  | rs17025005 | T/C | 0.2656 | 0.2757 | 0.3178 |
|  |  | rs7605434 | G/A | 0.1955 | 0.5014 | 0.2001 |
|  |  | rs13429998 | G/A | 0.0416 | 0.8541 | 0.0409 |
|  |  | rs4851384 | A/G | 0.8070 | 0.6002 | 1.0000 |
|  |  | rs3820787 | G/A | 0.4352 | 0.6546 | 0.4371 |
|  |  | rs4851386 | T/C | 0.0615 | 0.0727 | 0.1525 |
|  |  | rs73945847 | T/C | 0.1395 | 0.0805 | 0.5009 |
|  |  | rs4851390 | G/A | 0.2243 | 0.1639 | 0.5546 |
|  |  | rs882272 | A/G | 0.1125 | 0.6492 | 0.1135 |
|  |  | rs895520 | A/G | 0.1120 | 0.6527 | 0.1130 |
|  |  | rs6738097 | C/T | 0.0908 | 0.0432 | 0.1456 |
|  |  | rs12622050 | G/A | 0.0401 | 0.3814 | 0.0383 |
|  |  | rs17025078 | A/G | 0.5458 | 0.3531 | 0.6741 |
|  |  | rs4851391 | C/G | 0.1037 | 0.9569 | 0.0725 |
|  |  | rs3768985 | C/T | 0.3352 | 0.4607 | 0.4065 |
|  |  | rs4851392 | A/G | 0.8101 | 0.6306 | 0.7652 |
|  |  | rs2289950 | T/C | 0.3168 | 0.4501 | 0.3820 |
|  |  | rs4851393 | T/C | 0.0691 | 0.1161 | 0.1528 |
|  |  | rs1562313 | T/C | 0.1848 | 0.1026 | 0.2673 |
|  |  | rs2305160 | A/G | 0.8161 | 0.7249 | 0.7799 |
|  |  | rs2305159 | A/C | 0.4288 | 0.8771 | 0.3879 |
|  |  | rs1542179 | A/G | 0.3352 | 0.8821 | 0.3160 |
|  |  | rs1542178 | A/G | 0.6618 | 0.9390 | 0.6549 |
|  |  | rs3768988 | G/A | 0.7494 | 0.6488 | 0.5448 |
|  |  | rs62152925 | T/C | 0.1009 | 0.4636 | 0.1056 |
|  |  | rs75763901 | T/C | 0.5472 | 0.4196 | 0.5718 |
|  |  | rs75159075 | A/C | 0.2059 | 0.1866 | 0.2230 |
|  |  | rs2278728 | T/C | 0.7221 | 0.5424 | 0.7754 |
|  |  | rs2278727 | T/C | 0.0609 | 0.7368 | 0.0338 |
|  |  | rs6719533 | G/A | 0.3591 | 0.0800 | 0.8208 |
|  |  | rs3754677 | C/T | 0.7482 | 0.9625 | 0.7093 |
|  |  | rs3754678 | G/A | 0.8279 | 0.9235 | 0.7558 |
|  |  | rs3754680 | C/T | 0.9543 | 0.9697 | 0.9473 |
|  |  | rs3768990 | C/T | 0.9132 | 0.6688 | 0.9332 |
|  |  | rs9223 | T/C | 0.4812 | 0.3110 | 0.5713 |
|  |  | rs3739008 | T/C | 0.3689 | 0.2727 | 0.4454 |
| *NR1D1* | 17 | rs883871 | G/A | 0.0735 | 0.4686 | 0.0519 |
|  |  | rs2071427 | C/T | 0.2880 | 0.7398 | 0.2047 |
|  |  | rs2269457 | T/C | 0.9147 | 0.9678 | 0.9021 |
|  |  | rs12941497 | G/A | 0.7681 | 0.8483 | 0.7774 |
|  |  | rs939347 | G/A | 0.7217 | 0.8130 | 0.7365 |
|  |  | rs2071570 | C/A | 0.8057 | 0.8673 | 0.8181 |
| *NR1D2* | 3 | rs6778577 | T/G | 0.9974 | 0.6492 | 0.9974 |
|  |  | rs34593533 | A/G | 0.9975 | 0.6641 | 0.9975 |
|  |  | rs11922609 | G/A | 0.8475 | 0.0966 | 0.9234 |
|  |  | rs4858095 | T/G | 0.8810 | 0.2925 | 0.5023 |
|  |  | rs61556088 | A/G | 0.5113 | 0.9547 | 0.5083 |
|  |  | rs13095392 | A/T | 0.7618 | 0.2609 | 0.6074 |
|  |  | rs35962983 | T/C | 0.9799 | 0.2975 | 0.5025 |
|  |  | rs11717862 | G/A | 0.5621 | 0.0873 | 0.4443 |
|  |  | rs9882735 | T/C | 0.4889 | 0.6642 | 0.1646 |
|  |  | rs76873718 | G/A | 0.3589 | 0.9904 | 0.3152 |
| *PER1* | 17 | rs2304911 | G/A | 0.7030 | 0.0760 | 0.9301 |
| *PER2* | 2 | rs934945 | T/C | 0.9450 | 0.7313 | 0.8756 |
|  |  | rs6431590 | G/A | 0.9168 | 0.0997 | 0.6245 |
|  |  | rs2304669 | C/T | 0.7578 | 0.6004 | 0.7757 |
|  |  | rs3739064 | G/A | 0.8435 | 0.8100 | 0.8220 |
|  |  | rs1972874 | C/G | 0.7252 | 0.5307 | 0.5331 |
|  |  | rs2304677 | T/C | 0.7892 | 0.8562 | 0.7811 |
|  |  | rs2304674 | G/A | 0.9545 | 0.1544 | 0.6882 |
|  |  | rs71426512 | T/C | 0.2751 | 0.7582 | 0.2696 |
|  |  | rs67860414 | T/G | 0.8317 | 0.0770 | 0.9034 |
| *PER3* | 1 | rs228727 | T/C | 0.0655 | 0.8891 | 0.0134 |
|  |  | rs11121023 | A/G | 0.2700 | 0.7540 | 0.2634 |
|  |  | rs75307902 | T/C | 0.4068 | 0.5975 | 0.3807 |
|  |  | rs10864315 | T/C | 0.3079 | 0.6644 | 0.3111 |
|  |  | rs117562183 | A/G | 0.6031 | 0.3736 | 0.5734 |
|  |  | rs228682 | C/T | 0.3194 | 0.1530 | 0.3967 |
|  |  | rs77567305 | C/T | 0.3458 | 0.0519 | 0.3801 |
|  |  | rs10746473 | G/A | 0.0116 | 0.0010 | 0.3691 |
|  |  | rs2797685 | C/T | 0.0314 | 0.0047 | 0.5731 |
|  |  | rs118049345 | T/C | 0.8118 | 0.1137 | 0.7815 |
|  |  | rs1773135 | A/G | 0.9963 | 0.5100 | 0.9617 |
|  |  | rs1689904 | C/T | 0.0352 | 0.0061 | 0.5618 |
|  |  | rs1773138 | T/C | 0.0304 | 0.5902 | 0.0043 |
|  |  | rs12563789 | G/A | 0.6475 | 0.0299 | 0.8946 |
| *REV1* | 2 | rs2305354 | G/A | 0.5623 | 0.3260 | 0.2547 |
|  |  | rs28382964 | T/G | 0.7226 | 0.4358 | 0.7490 |
|  |  | rs2290261 | G/A | 0.7193 | 0.5658 | 0.5449 |
|  |  | rs4535093 | G/A | 0.9263 | 0.1947 | 0.5114 |
|  |  | rs12619546 | A/G | 0.5391 | 0.8412 | 0.5335 |
|  |  | rs4341989 | T/C | 0.2673 | 0.2632 | 0.2821 |
|  |  | rs3792146 | T/C | 0.7765 | 0.2664 | 0.8975 |
|  |  | rs7585019 | C/G | 0.4718 | 0.8015 | 0.3536 |
|  |  | rs28745277 | C/T | 0.0429 | 0.7108 | 0.0426 |
| *RORA* | 15 | rs75461420 | A/C | 0.4892 | 0.4102 | 0.5010 |
|  |  | rs3743266 | C/T | 0.7396 | 0.8400 | 0.7487 |
|  |  | rs17270188 | A/G | 0.2676 | 0.7008 | 0.1702 |
|  |  | rs78280589 | G/A | 0.3187 | 0.0979 | 0.3473 |
|  |  | rs6494204 | C/T | 0.2750 | 0.4874 | 0.2227 |
|  |  | rs10438338 | C/T | 0.5253 | 0.1727 | 0.6426 |
|  |  | rs1866007 | G/A | 0.2124 | 0.3625 | 0.1971 |
|  |  | rs10519051 | G/A | 0.4323 | 0.5248 | 0.4492 |
|  |  | rs17237283 | C/T | 0.0811 | 0.5101 | 0.0840 |
|  |  | rs4594196 | C/T | 0.4970 | 0.7110 | 0.5086 |
|  |  | rs4774367 | G/C | 0.2265 | 0.3459 | 0.2124 |
|  |  | rs11635975 | G/A | 0.9081 | 0.2947 | 0.9839 |
|  |  | rs2028122 | A/G | 0.8632 | 0.0945 | 0.7752 |
|  |  | rs8033552 | A/G | 0.9141 | 0.1828 | 0.7852 |
|  |  | rs4775281 | C/A | 0.2761 | 0.1865 | 0.3971 |
|  |  | rs116861339 | C/T | 0.7190 | 0.7294 | 0.7091 |
|  |  | rs79360097 | C/T | 0.7024 | 0.1113 | 0.6632 |
|  |  | rs8041381 | G/A | 0.4303 | 0.3073 | 0.3888 |
|  |  | rs16942772 | T/G | 0.1959 | 0.5348 | 0.2014 |
|  |  | rs340002 | A/G | 0.8114 | 0.7585 | 0.6938 |
|  |  | rs11632600 | T/G | 0.8223 | 0.4526 | 0.7109 |
|  |  | rs11634234 | T/C | 0.7156 | 0.6116 | 0.5800 |
|  |  | rs340005 | G/A | 0.7109 | 0.6360 | 0.6908 |
|  |  | rs2289162 | A/T | 0.3319 | 0.9869 | 0.3246 |
|  |  | rs2289163 | C/A | 0.3055 | 0.8905 | 0.3017 |
|  |  | rs339969 | C/A | 0.6718 | 0.6108 | 0.6504 |
|  |  | rs12443044 | A/T | 0.7893 | 0.6810 | 0.4567 |
|  |  | rs72748739 | C/T | 0.2820 | 0.9505 | 0.2758 |
|  |  | rs340009 | A/C | 0.4474 | 0.7652 | 0.1804 |
|  |  | rs12591786 | T/C | 0.5010 | 0.9795 | 0.4977 |
|  |  | rs58306294 | T/C | 0.4119 | 0.6971 | 0.4194 |
|  |  | rs340021 | G/C | 0.3745 | 0.8839 | 0.3713 |
|  |  | rs340023 | C/T | 0.3573 | 0.1909 | 0.6431 |
|  |  | rs340026 | G/A | 0.1208 | 0.4015 | 0.1289 |
|  |  | rs3784611 | G/C | 0.1356 | 0.4688 | 0.1424 |
|  |  | rs3784610 | A/C | 0.7128 | 0.8441 | 0.5077 |
|  |  | rs3784609 | T/C | 0.9975 | 0.0571 | 0.9975 |
|  |  | rs72748759 | A/G | 0.9975 | 0.0836 | 0.9975 |
|  |  | rs28408562 | C/G | 0.7160 | 0.1589 | 0.0906 |
|  |  | rs28724570 | C/T | 0.8674 | 0.1585 | 0.1620 |
|  |  | rs75981965 | C/T | 0.2151 | 0.9931 | 0.2133 |
|  |  | rs72748764 | T/C | 0.9976 | 0.2476 | 0.9976 |
|  |  | rs1657792 | T/C | 0.6218 | 0.7281 | 0.5245 |
|  |  | rs11630262 | A/G | 0.9976 | 0.4237 | 0.9976 |
|  |  | rs78746013 | C/T | 0.3296 | 0.8995 | 0.3276 |
|  |  | rs75995412 | A/G | 0.3383 | 0.8821 | 0.3365 |
|  |  | rs2241794 | T/C | 0.9153 | 0.5999 | 0.9282 |
|  |  | rs80313728 | C/T | 0.9268 | 0.7630 | 0.9340 |
|  |  | rs339995 | A/G | 0.6198 | 0.7450 | 0.6284 |
|  |  | rs339996 | C/T | 0.4194 | 0.1691 | 0.5070 |
|  |  | rs17237318 | C/T | 0.7435 | 0.3189 | 0.6005 |
|  |  | rs339998 | T/C | 0.4840 | 0.6140 | 0.5312 |
|  |  | rs9630427 | C/T | 0.0822 | 0.3913 | 0.0895 |
|  |  | rs16942900 | T/C | 0.8601 | 0.9906 | 0.8468 |
|  |  | rs79416181 | C/T | 0.7336 | 0.6521 | 0.7267 |
|  |  | rs2433026 | C/G | 0.6106 | 0.9113 | 0.5685 |
|  |  | rs41356552 | G/A | 0.2087 | 0.4969 | 0.2192 |
|  |  | rs7166370 | C/G | 0.3544 | 0.5390 | 0.3926 |
|  |  | rs12437754 | A/G | 0.1731 | 0.1933 | 0.2517 |
|  |  | rs1657800 | C/T | 0.3294 | 0.6801 | 0.2215 |
|  |  | rs11629660 | C/A | 0.3570 | 0.6071 | 0.2313 |
|  |  | rs11629864 | C/G | 0.9833 | 0.8846 | 0.9988 |
|  |  | rs7172874 | C/T | 0.3655 | 0.7440 | 0.2494 |
|  |  | rs74800820 | G/A | 0.7285 | 0.4441 | 0.7192 |
|  |  | rs2553234 | T/C | 0.6474 | 0.9729 | 0.6296 |
|  |  | rs9920661 | C/A | 0.9766 | 0.8529 | 0.9935 |
|  |  | rs9920767 | A/G | 0.6968 | 0.7894 | 0.6638 |
|  |  | rs76105700 | G/A | 0.9381 | 0.8315 | 0.9443 |
|  |  | rs880626 | A/G | 0.2322 | 0.6243 | 0.1590 |
|  |  | rs880625 | G/A | 0.1405 | 0.7868 | 0.0952 |
|  |  | rs2553236 | C/T | 0.5516 | 0.1544 | 0.8223 |
|  |  | rs8040332 | A/T | 0.9864 | 0.0899 | 0.9552 |
|  |  | rs8042370 | T/C | 0.6099 | 0.6650 | 0.6666 |
|  |  | rs919000 | G/A | 0.8158 | 0.7768 | 0.8587 |
|  |  | rs999449 | A/C | 0.7319 | 0.2415 | 0.9171 |
|  |  | rs11629812 | A/G | 0.4370 | 0.3089 | 0.5467 |
|  |  | rs59558657 | T/C | 0.4735 | 0.9411 | 0.4594 |
|  |  | rs6494217 | A/G | 0.5787 | 0.5065 | 0.3847 |
|  |  | rs118138621 | T/G | 0.4594 | 0.9765 | 0.4546 |
|  |  | rs1425287 | A/G | 0.3781 | 0.7613 | 0.3766 |
|  |  | rs78164583 | A/C | 0.5882 | 0.5842 | 0.6022 |
|  |  | rs117194204 | A/G | 0.3662 | 0.8283 | 0.3667 |
|  |  | rs13329238 | C/A | 0.1872 | 0.0858 | 0.2804 |
|  |  | rs4774371 | A/G | 0.5721 | 0.5272 | 0.6072 |
|  |  | rs17237346 | T/C | 0.3626 | 0.6923 | 0.3660 |
|  |  | rs8027032 | T/C | 0.2917 | 0.4462 | 0.3373 |
|  |  | rs8038077 | C/T | 0.7331 | 0.7373 | 0.7571 |
|  |  | rs2433025 | A/G | 0.3318 | 0.1484 | 0.4575 |
|  |  | rs17303111 | T/C | 0.3069 | 0.3889 | 0.3791 |
|  |  | rs7173461 | C/G | 0.4618 | 0.3606 | 0.5002 |
|  |  | rs2414680 | A/G | 0.2912 | 0.3164 | 0.3841 |
|  |  | rs6494219 | C/T | 0.4936 | 0.4074 | 0.4098 |
|  |  | rs6494221 | C/A | 0.8676 | 0.2254 | 0.9326 |
|  |  | rs12899193 | T/C | 0.1342 | 0.8303 | 0.0766 |
|  |  | rs16943000 | G/A | 0.1184 | 0.9594 | 0.1169 |
|  |  | rs11071551 | G/C | 0.8099 | 0.7463 | 0.7784 |
|  |  | rs16943012 | G/C | 0.3103 | 0.9518 | 0.3095 |
|  |  | rs1834335 | T/C | 0.6726 | 0.5923 | 0.6260 |
|  |  | rs1820357 | T/G | 0.0558 | 0.1639 | 0.0866 |
|  |  | rs17237353 | A/G | 0.1946 | 0.5837 | 0.1632 |
|  |  | rs12591749 | C/T | 0.2074 | 0.2756 | 0.3078 |
|  |  | rs341413 | C/T | 0.9961 | 0.3865 | 0.9262 |
|  |  | rs35277300 | C/T | 0.1039 | 0.0470 | 0.2805 |
|  |  | rs17237367 | A/G | 0.0263 | 0.0036 | 0.0926 |
|  |  | rs78554936 | G/T | 0.0838 | 0.1839 | 0.0912 |
|  |  | rs7168905 | G/T | 0.8878 | 0.0110 | 0.9694 |
|  |  | rs9920962 | G/A | 0.7955 | 0.6639 | 0.9519 |
|  |  | rs76194223 | T/C | 0.0385 | 0.2289 | 0.0414 |
|  |  | rs4775292 | T/C | 0.9978 | 0.1248 | 0.9978 |
|  |  | rs7172011 | T/C | 0.6791 | 0.3360 | 0.8506 |
|  |  | rs1993471 | A/C | 0.8008 | 0.9674 | 0.8002 |
|  |  | rs17204367 | A/G | 0.1332 | 0.1896 | 0.2527 |
|  |  | rs17303153 | G/A | 0.0140 | 0.1250 | 0.0181 |
|  |  | rs1020729 | C/T | 0.0894 | 0.1538 | 0.1677 |
|  |  | rs58469372 | A/G | 0.0092 | 0.1113 | 0.0129 |
|  |  | rs1020730 | T/C | 0.1089 | 0.5505 | 0.1077 |
|  |  | rs17204402 | C/G | 0.9893 | 0.3940 | 0.9390 |
|  |  | rs961299 | A/G | 0.0661 | 0.8254 | 0.0623 |
|  |  | rs12900122 | T/C | 0.3324 | 0.2194 | 0.3924 |
|  |  | rs2279297 | C/T | 0.6502 | 0.1062 | 0.7660 |
|  |  | rs8025689 | C/G | 0.0855 | 0.2337 | 0.0998 |
|  |  | rs17204426 | G/T | 0.7338 | 0.2342 | 0.8474 |
|  |  | rs62002747 | C/T | 0.6008 | 0.2109 | 0.7075 |
|  |  | rs9302215 | C/T | 0.1042 | 0.0330 | 0.2917 |
|  |  | rs12591650 | A/G | 0.0109 | 0.0049 | 0.1948 |
|  |  | rs1482057 | A/C | 0.6647 | 0.3757 | 0.6003 |
|  |  | rs17204440 | C/A | 0.3956 | 0.1337 | 0.4870 |
|  |  | rs11639084 | T/C | 0.1583 | 0.1502 | 0.1930 |
|  |  | rs12594188 | C/T | 0.0389 | 0.0035 | 0.1272 |
|  |  | rs10519067 | A/G | 0.2628 | 0.7616 | 0.2633 |
|  |  | rs12438866 | C/T | 0.7279 | 0.6770 | 0.6163 |
|  |  | rs10519070 | T/C | 0.0914 | 0.2107 | 0.1328 |
|  |  | rs62002749 | A/G | 0.6452 | 0.0323 | 0.8730 |
|  |  | rs11071557 | C/T | 0.9697 | 0.8086 | 0.9919 |
|  |  | rs11071558 | G/A | 0.8705 | 0.8119 | 0.8427 |
|  |  | rs11071559 | T/C | 0.9954 | 0.7599 | 0.9659 |
|  |  | rs922782 | G/T | 0.9572 | 0.1349 | 0.6941 |
|  |  | rs922781 | C/G | 0.9656 | 0.2316 | 0.7539 |
|  |  | rs4774372 | C/T | 0.0669 | 0.1964 | 0.0895 |
|  |  | rs1963497 | A/C | 0.6140 | 0.0515 | 0.6928 |
|  |  | rs17270446 | G/C | 0.8455 | 0.0021 | 0.6109 |
|  |  | rs2899662 | T/C | 0.8705 | 0.0213 | 0.9943 |
|  |  | rs1680446 | T/C | 0.9054 | 0.0390 | 0.8669 |
|  |  | rs877228 | G/A | 0.1977 | 0.5416 | 0.0456 |
|  |  | rs4775297 | T/C | 0.2628 | 0.0502 | 0.3749 |
|  |  | rs16943117 | T/C | 0.6930 | 0.4679 | 0.7553 |
|  |  | rs12915776 | A/G | 0.7370 | 0.6039 | 0.7776 |
|  |  | rs341459 | C/T | 0.4522 | 0.8862 | 0.4491 |
|  |  | rs12593925 | T/C | 0.1064 | 0.2174 | 0.1622 |
|  |  | rs78498480 | C/G | 0.2385 | 0.2635 | 0.3961 |
|  |  | rs10162630 | A/G | 0.1783 | 0.8687 | 0.1302 |
|  |  | rs12591848 | A/C | 0.2721 | 0.4393 | 0.3145 |
|  |  | rs12440185 | T/C | 0.0519 | 0.7470 | 0.0518 |
|  |  | rs12902540 | T/C | 0.3454 | 0.5233 | 0.3655 |
|  |  | rs7162615 | A/G | 0.0248 | 0.2373 | 0.0299 |
|  |  | rs17270459 | T/C | 0.7483 | 0.1079 | 0.6907 |
|  |  | rs875339 | T/C | 0.6629 | 0.0509 | 0.9239 |
|  |  | rs62004360 | T/C | 0.7004 | 0.1527 | 0.6491 |
|  |  | rs341366 | A/G | 0.6025 | 0.7840 | 0.5635 |
|  |  | rs16943131 | C/T | 0.0565 | 0.7550 | 0.0567 |
|  |  | rs75866172 | A/G | 0.0566 | 0.8415 | 0.0565 |
|  |  | rs6494225 | G/T | 0.0566 | 0.6934 | 0.0570 |
|  |  | rs6494227 | C/A | 0.0518 | 0.2328 | 0.0574 |
|  |  | rs79610262 | T/C | 0.0526 | 0.2502 | 0.0579 |
|  |  | rs10519076 | C/G | 0.7654 | 0.0293 | 0.4645 |
|  |  | rs341373 | T/C | 0.3929 | 0.2082 | 0.4438 |
|  |  | rs78507043 | A/C | 0.8404 | 0.9973 | 0.7495 |
|  |  | rs10152719 | T/C | 0.7487 | 0.0473 | 0.4727 |
|  |  | rs341381 | G/A | 0.6242 | 0.1657 | 0.5595 |
|  |  | rs16943172 | T/C | 0.8411 | 0.4626 | 0.6578 |
|  |  | rs12439995 | G/C | 0.9858 | 0.0296 | 0.6326 |
|  |  | rs341392 | C/A | 0.6500 | 0.4891 | 0.3270 |
|  |  | rs6494229 | A/G | 0.5021 | 0.7686 | 0.4816 |
|  |  | rs8041061 | G/T | 0.3789 | 0.5791 | 0.3960 |
|  |  | rs8042149 | T/G | 0.3745 | 0.6733 | 0.3309 |
|  |  | rs4775301 | C/T | 0.5194 | 0.1037 | 0.9682 |
|  |  | rs6494230 | C/T | 0.3120 | 0.1023 | 0.8738 |
|  |  | rs11634976 | C/G | 0.4061 | 0.1418 | 0.9628 |
|  |  | rs8023252 | T/G | 0.8433 | 0.5123 | 0.5187 |
|  |  | rs341398 | G/A | 0.8253 | 0.6017 | 0.9275 |
|  |  | rs1224251 | G/A | 0.6235 | 0.0506 | 0.6911 |
|  |  | rs11630062 | C/T | 0.2267 | 0.0082 | 0.8834 |
|  |  | rs341403 | C/T | 0.7374 | 0.9849 | 0.7244 |
|  |  | rs12595623 | C/G | 0.6098 | 0.9192 | 0.5929 |
|  |  | rs11630227 | C/T | 0.4830 | 0.9318 | 0.3743 |
|  |  | rs10519080 | C/T | 0.3741 | 0.5106 | 0.2825 |
|  |  | rs341408 | T/C | 0.5220 | 0.4515 | 0.3462 |
|  |  | rs17204545 | G/T | 0.9971 | 0.9812 | 0.9971 |
|  |  | rs79409065 | A/C | 0.5734 | 0.8733 | 0.5612 |
|  |  | rs341411 | T/C | 0.3797 | 0.5694 | 0.3683 |
|  |  | rs75084363 | G/A | 0.3093 | 0.5808 | 0.3213 |
|  |  | rs11858268 | A/G | 0.3162 | 0.9389 | 0.3123 |
|  |  | rs7497885 | A/G | 0.4180 | 0.4809 | 0.3466 |
|  |  | rs2306502 | C/A | 0.9976 | 0.8015 | 0.9976 |
|  |  | rs10519085 | C/T | 0.4865 | 0.2447 | 0.6205 |
|  |  | rs8041466 | T/C | 0.6370 | 0.3026 | 0.7652 |
|  |  | rs12913890 | G/C | 0.8286 | 0.6337 | 0.9804 |
|  |  | rs72750668 | T/C | 0.5945 | 0.7330 | 0.6184 |
|  |  | rs77282013 | C/T | 0.9372 | 0.9482 | 0.9408 |
|  |  | rs1902618 | G/A | 0.5221 | 0.4035 | 0.5598 |
|  |  | rs341365 | A/G | 0.7409 | 0.6939 | 0.3784 |
|  |  | rs7182392 | T/C | 0.6332 | 0.4834 | 0.7008 |
|  |  | rs4775309 | A/G | 0.7056 | 0.3696 | 0.8513 |
|  |  | rs341387 | C/T | 0.5676 | 0.1388 | 0.1778 |
|  |  | rs11631432 | C/T | 0.5924 | 0.8833 | 0.3919 |
|  |  | rs4775311 | C/T | 0.8232 | 0.3803 | 0.8201 |
|  |  | rs8039990 | T/C | 0.5966 | 0.7751 | 0.4702 |
|  |  | rs8040450 | C/G | 0.7467 | 0.4671 | 0.5025 |
|  |  | rs341389 | A/G | 0.5111 | 0.7987 | 0.3930 |
|  |  | rs12907550 | T/C | 0.7180 | 0.7460 | 0.7084 |
|  |  | rs8036723 | A/G | 0.6478 | 0.9491 | 0.5840 |
|  |  | rs16943284 | C/T | 0.9109 | 0.7356 | 0.8525 |
|  |  | rs12915127 | T/C | 0.5931 | 0.4654 | 0.6131 |
|  |  | rs28692829 | T/C | 0.1512 | 0.5805 | 0.1519 |
|  |  | rs2414682 | C/T | 0.6741 | 0.3257 | 0.7852 |
|  |  | rs79067694 | C/A | 0.9989 | 0.0946 | 0.9989 |
|  |  | rs729977 | T/C | 0.7775 | 0.4900 | 0.4835 |
|  |  | rs7172917 | T/C | 0.8518 | 0.9858 | 0.8125 |
|  |  | rs4775313 | G/C | 0.8435 | 0.6040 | 0.7824 |
|  |  | rs4774376 | G/C | 0.5654 | 0.3179 | 0.7845 |
|  |  | rs35598844 | C/G | 0.3362 | 0.6687 | 0.3429 |
|  |  | rs7168782 | A/C | 0.3188 | 0.9336 | 0.3017 |
|  |  | rs117779544 | C/T | 0.9979 | 0.2207 | 0.9979 |
|  |  | rs8041087 | T/C | 0.9131 | 0.5393 | 0.9738 |
|  |  | rs2414686 | G/A | 0.8948 | 0.4293 | 0.4024 |
|  |  | rs877862 | G/A | 0.9519 | 0.5673 | 0.9226 |
|  |  | rs12904857 | A/G | 0.4722 | 0.8135 | 0.4742 |
|  |  | rs12910281 | C/T | 0.7346 | 0.0328 | 0.2407 |
|  |  | rs12909379 | A/G | 0.9081 | 0.1658 | 0.3057 |
|  |  | rs16943299 | A/G | 0.7205 | 0.5132 | 0.7779 |
|  |  | rs117795767 | C/A | 0.9989 | 0.1682 | 0.9989 |
|  |  | rs2899664 | A/G | 0.9125 | 0.5659 | 0.8224 |
|  |  | rs1054789 | T/A | 0.9070 | 0.0933 | 0.4083 |
|  |  | rs2062091 | C/T | 0.8219 | 0.0632 | 0.2263 |
|  |  | rs1384121 | A/C | 0.1952 | 0.0927 | 0.2790 |
|  |  | rs11855147 | C/T | 0.5432 | 0.7233 | 0.5496 |
|  |  | rs8027424 | C/A | 0.1209 | 0.0552 | 0.3776 |
|  |  | rs17237486 | T/C | 0.3492 | 0.0568 | 0.8307 |
|  |  | rs7162937 | C/G | 0.3768 | 0.7902 | 0.3656 |
|  |  | rs12148149 | C/T | 0.3715 | 0.8793 | 0.3630 |
|  |  | rs12901574 | A/C | 0.1428 | 0.0810 | 0.3581 |
|  |  | rs5813053 | C/N | 0.0604 | 0.1177 | 0.1189 |
|  |  | rs6494232 | A/G | 0.2554 | 0.1109 | 0.6796 |
|  |  | rs16943318 | A/G | 0.2475 | 0.9170 | 0.2401 |
|  |  | rs8028796 | C/T | 0.9574 | 0.4738 | 0.8937 |
|  |  | rs4775318 | T/A | 0.3377 | 0.8398 | 0.3110 |
|  |  | rs2062094 | T/C | 0.2654 | 0.7917 | 0.1907 |
|  |  | rs2062092 | C/T | 0.5381 | 0.8748 | 0.5243 |
|  |  | rs1482052 | T/G | 0.4586 | 0.6461 | 0.4528 |
|  |  | rs10220727 | T/C | 0.2491 | 0.7701 | 0.2140 |
|  |  | rs17303258 | G/A | 0.3493 | 0.9059 | 0.3449 |
|  |  | rs35715615 | G/A | 0.4130 | 0.5599 | 0.2024 |
|  |  | rs2279291 | T/G | 0.1985 | 0.8706 | 0.1417 |
|  |  | rs1482049 | C/T | 0.1929 | 0.7400 | 0.1253 |
|  |  | rs79995443 | C/A | 0.3030 | 0.1929 | 0.3221 |
|  |  | rs28705880 | T/G | 0.0238 | 0.0885 | 0.0483 |
|  |  | rs1351545 | T/C | 0.4479 | 0.8335 | 0.3890 |
|  |  | rs8034886 | A/G | 0.1863 | 0.5151 | 0.1503 |
|  |  | rs4775328 | T/C | 0.1346 | 0.3320 | 0.1511 |
|  |  | rs79271390 | C/T | 0.9832 | 0.9269 | 0.9784 |
|  |  | rs58413143 | C/A | 0.1157 | 0.8219 | 0.0773 |
|  |  | rs7176774 | T/C | 0.6406 | 0.7289 | 0.6821 |
|  |  | rs72752780 | C/A | 0.0457 | 0.4306 | 0.0471 |
|  |  | rs4335725 | T/C | 0.2005 | 0.2729 | 0.1588 |
|  |  | rs12903220 | T/C | 0.4543 | 0.1635 | 0.3393 |
|  |  | rs12593927 | G/C | 0.1806 | 0.3543 | 0.2186 |
|  |  | rs8029848 | G/A | 0.0127 | 0.0004 | 0.0629 |
|  |  | rs8034880 | G/A | 0.0150 | 0.0002 | 0.0711 |
|  |  | rs8034950 | C/T | 0.0341 | 0.0466 | 0.1144 |
|  |  | rs28575275 | A/G | 0.1774 | 0.8754 | 0.1171 |
|  |  | rs12912233 | T/C | 0.3407 | 0.0580 | 0.5344 |
|  |  | rs4775339 | A/G | 0.7172 | 0.8591 | 0.7201 |
|  |  | rs4775340 | A/G | 0.7009 | 0.0271 | 0.8594 |
|  |  | rs17237521 | T/C | 0.0234 | 0.2443 | 0.0228 |
|  |  | rs6494237 | C/T | 0.0710 | 0.3570 | 0.0766 |
|  |  | rs72752802 | C/A | 0.0053 | 0.1716 | 0.0045 |
|  |  | rs2140442 | T/C | 0.5365 | 0.5124 | 0.5492 |
|  |  | rs7168987 | T/C | 0.4092 | 0.2539 | 0.4225 |
|  |  | rs11631656 | G/A | 0.0186 | 0.4531 | 0.0188 |
|  |  | rs7176329 | T/C | 0.7381 | 0.1715 | 0.9679 |
|  |  | rs16943444 | A/G | 0.3779 | 0.2576 | 0.5960 |
|  |  | rs1467304 | C/T | 0.0294 | 0.3874 | 0.0305 |
|  |  | rs7174217 | T/C | 0.2559 | 0.2146 | 0.4443 |
|  |  | rs7171713 | T/C | 0.8411 | 0.2942 | 0.7136 |
|  |  | rs34299559 | N/G | 0.2417 | 0.2157 | 0.4161 |
|  |  | rs16943453 | G/T | 0.6469 | 0.2335 | 0.8288 |
|  |  | rs7174288 | A/G | 0.7726 | 0.6618 | 0.5302 |
|  |  | rs11637844 | G/A | 0.9032 | 0.9553 | 0.8935 |
|  |  | rs10519097 | T/C | 0.8890 | 0.8788 | 0.8759 |
|  |  | rs17204770 | C/T | 0.0928 | 0.4272 | 0.0991 |
|  |  | rs2030619 | G/T | 0.1147 | 0.5808 | 0.0736 |
|  |  | rs11638929 | C/T | 0.5013 | 0.0924 | 0.9951 |
|  |  | rs17237563 | T/C | 0.9150 | 0.3000 | 0.8597 |
|  |  | rs1523530 | A/T | 0.5086 | 0.3650 | 0.6319 |
|  |  | rs62005615 | A/C | 0.4473 | 0.0459 | 0.4756 |
|  |  | rs72625740 | C/T | 0.0407 | 0.6828 | 0.0344 |
|  |  | rs60257905 | T/C | 0.1923 | 0.3183 | 0.2247 |
|  |  | rs17237570 | C/T | 0.1210 | 0.0930 | 0.3751 |
|  |  | rs8040930 | G/A | 0.0906 | 0.8345 | 0.0774 |
|  |  | rs12898479 | G/A | 0.0810 | 0.7742 | 0.0706 |
|  |  | rs12592612 | C/T | 0.1355 | 0.0838 | 0.1712 |
|  |  | rs17303341 | C/T | 0.5003 | 0.5448 | 0.5502 |
|  |  | rs75336871 | A/G | 0.4682 | 0.5049 | 0.5212 |
|  |  | rs17303355 | C/G | 0.0862 | 0.7554 | 0.0669 |
|  |  | rs4775349 | C/T | 0.7005 | 0.1195 | 0.0994 |
|  |  | rs72625742 | T/C | 0.0208 | 0.6951 | 0.0127 |
|  |  | rs1403739 | G/A | 0.0437 | 0.7018 | 0.0305 |
|  |  | rs17303369 | T/C | 0.4499 | 0.1870 | 0.3075 |
|  |  | rs6494243 | G/A | 0.0327 | 0.1430 | 0.0368 |
|  |  | rs12438879 | G/T | 0.4932 | 0.5136 | 0.2990 |
|  |  | rs74687025 | G/A | 0.5438 | 0.2168 | 0.6145 |
|  |  | rs10519107 | C/G | 0.4356 | 0.5181 | 0.2559 |
|  |  | rs116919391 | A/C | 0.6615 | 0.9986 | 0.6600 |
|  |  | rs809736 | G/A | 0.0735 | 0.1926 | 0.0829 |
|  |  | rs2280595 | A/T | 0.4385 | 0.7880 | 0.4416 |
|  |  | rs4775350 | C/T | 0.5344 | 0.2364 | 0.3830 |
|  |  | rs1437549 | G/A | 0.8294 | 0.4729 | 0.8072 |
|  |  | rs4775351 | T/C | 0.1710 | 0.4397 | 0.1791 |
|  |  | rs7172342 | C/G | 0.2511 | 0.2871 | 0.3913 |
|  |  | rs4774384 | T/C | 0.6233 | 0.6677 | 0.5102 |
|  |  | rs782944 | A/C | 0.7620 | 0.8338 | 0.7824 |
|  |  | rs10519108 | C/G | 0.7249 | 0.1095 | 0.6694 |
|  |  | rs782948 | A/G | 0.0432 | 0.3314 | 0.0453 |
|  |  | rs2247306 | G/A | 0.2193 | 0.1900 | 0.4463 |
|  |  | rs12324535 | A/G | 0.4077 | 0.3128 | 0.6154 |
|  |  | rs873962 | A/G | 0.3120 | 0.6581 | 0.2938 |
|  |  | rs873961 | T/C | 0.4732 | 0.3462 | 0.3265 |
|  |  | rs8037669 | A/G | 0.7494 | 0.3675 | 0.4343 |
|  |  | rs7173460 | A/G | 0.0339 | 0.4917 | 0.0273 |
|  |  | rs6494246 | T/C | 0.6569 | 0.5312 | 0.7279 |
|  |  | rs78100524 | A/G | 0.8349 | 0.5071 | 0.8014 |
|  |  | rs782956 | G/T | 0.1817 | 0.4221 | 0.2017 |
|  |  | rs11632352 | T/C | 0.9663 | 0.2783 | 0.6831 |
|  |  | rs782903 | G/T | 0.5257 | 0.9270 | 0.4440 |
|  |  | rs16943579 | A/G | 0.6379 | 0.3751 | 0.7328 |
|  |  | rs11854619 | C/T | 0.4777 | 0.4837 | 0.4858 |
|  |  | rs12915672 | C/G | 0.7533 | 0.9631 | 0.7297 |
|  |  | rs12915830 | A/G | 0.8963 | 0.9500 | 0.8920 |
|  |  | rs708680 | G/C | 0.4961 | 0.1146 | 0.7942 |
|  |  | rs12903172 | C/T | 0.7915 | 0.5796 | 0.9291 |
|  |  | rs2689352 | C/T | 0.0776 | 0.0596 | 0.2345 |
|  |  | rs940222 | G/T | 0.2698 | 0.4063 | 0.3275 |
|  |  | rs62005642 | T/G | 0.2031 | 0.0921 | 0.2362 |
|  |  | rs7171287 | G/C | 0.6671 | 0.1349 | 0.8378 |
|  |  | rs1437551 | G/A | 0.1864 | 0.1019 | 0.2162 |
|  |  | rs77786240 | A/G | 0.0435 | 0.1736 | 0.0475 |
|  |  | rs782907 | A/G | 0.9556 | 0.5828 | 0.7456 |
|  |  | rs782908 | A/G | 0.2967 | 0.5234 | 0.3117 |
|  |  | rs4774386 | C/T | 0.8248 | 0.8037 | 0.8503 |
|  |  | rs893286 | C/A | 0.9449 | 0.6390 | 0.8279 |
|  |  | rs718911 | G/A | 0.9946 | 0.5582 | 0.9048 |
|  |  | rs76853459 | T/G | 0.1568 | 0.5122 | 0.1614 |
|  |  | rs12902142 | C/T | 0.7896 | 0.9862 | 0.6937 |
|  |  | rs4775356 | C/T | 0.4497 | 0.8810 | 0.4322 |
|  |  | rs782910 | A/G | 0.4698 | 0.3197 | 0.5208 |
|  |  | rs8035885 | A/G | 0.3719 | 0.9026 | 0.3569 |
|  |  | rs76824799 | T/C | 0.3895 | 0.6505 | 0.3607 |
|  |  | rs60094610 | A/G | 0.8952 | 0.1610 | 0.8088 |
|  |  | rs1437535 | T/C | 0.8523 | 0.9235 | 0.8526 |
|  |  | rs1437537 | T/C | 0.8062 | 0.8933 | 0.8097 |
|  |  | rs8042259 | T/C | 0.7270 | 0.5934 | 0.5740 |
|  |  | rs76431303 | T/C | 0.9546 | 0.1199 | 0.9561 |
|  |  | rs3803479 | A/G | 0.7600 | 0.1416 | 0.4814 |
|  |  | rs893288 | C/T | 0.3404 | 0.7029 | 0.3323 |
|  |  | rs78573683 | A/G | 0.1854 | 0.6754 | 0.1860 |
|  |  | rs13329643 | T/C | 0.0356 | 0.5597 | 0.0359 |
|  |  | rs782915 | A/G | 0.2561 | 0.8956 | 0.2351 |
|  |  | rs8024672 | A/C | 0.7452 | 0.0455 | 0.6424 |
|  |  | rs782919 | T/C | 0.6521 | 0.3810 | 0.7918 |
|  |  | rs782931 | G/A | 0.7109 | 0.4247 | 0.9659 |
|  |  | rs782933 | A/C | 0.4414 | 0.9443 | 0.3713 |
|  |  | rs782935 | C/T | 0.3326 | 0.9692 | 0.1682 |
|  |  | rs17303474 | C/T | 0.8042 | 0.9660 | 0.7892 |
|  |  | rs782937 | A/C | 0.2737 | 0.7633 | 0.2509 |
|  |  | rs16943672 | T/A | 0.9977 | 0.2316 | 0.9977 |
|  |  | rs4775360 | G/C | 0.6772 | 0.7087 | 0.7157 |
|  |  | rs7183595 | C/G | 0.7750 | 0.5354 | 0.9093 |
|  |  | rs11634887 | T/C | 0.7270 | 0.8151 | 0.7414 |
|  |  | rs719006 | T/A | 0.8623 | 0.6810 | 0.9543 |
|  |  | rs1160694 | G/A | 0.8902 | 0.5625 | 0.7183 |
|  |  | rs1159814 | T/C | 0.7849 | 0.9289 | 0.7778 |
|  |  | rs78512626 | C/A | 0.5949 | 0.8282 | 0.5811 |
|  |  | rs9788699 | T/C | 0.9163 | 0.8804 | 0.8788 |
|  |  | rs9788704 | T/C | 0.9285 | 0.1873 | 0.3662 |
|  |  | rs11071587 | G/A | 0.5765 | 0.3393 | 0.9923 |
|  |  | rs11071588 | T/G | 0.9368 | 0.7103 | 0.8624 |
|  |  | rs9788745 | G/A | 0.5671 | 0.6735 | 0.6033 |
|  |  | rs7163680 | G/T | 0.4804 | 0.7467 | 0.4210 |
|  |  | rs12900813 | T/C | 0.5211 | 0.7378 | 0.4865 |
|  |  | rs12900948 | T/C | 0.5199 | 0.7622 | 0.4679 |
|  |  | rs4238351 | A/G | 0.4473 | 0.1829 | 0.7418 |
|  |  | rs12592385 | T/C | 0.2235 | 0.7289 | 0.1880 |
|  |  | rs12900176 | T/C | 0.4421 | 0.9107 | 0.3014 |
|  |  | rs737112 | C/T | 0.4960 | 0.8932 | 0.3437 |
|  |  | rs17237759 | G/T | 0.8540 | 0.1858 | 0.9787 |
|  |  | rs17303509 | T/C | 0.7921 | 0.9956 | 0.7812 |
|  |  | rs1370433 | A/T | 0.3319 | 0.4197 | 0.4164 |
|  |  | rs117080246 | T/G | 0.6204 | 0.3546 | 0.6430 |
|  |  | rs17303523 | C/G | 0.0862 | 0.5847 | 0.0667 |
|  |  | rs17303530 | G/T | 0.0583 | 0.5478 | 0.0432 |
|  |  | rs4775368 | T/C | 0.2924 | 0.8461 | 0.1780 |
|  |  | rs11071590 | G/A | 0.2695 | 0.6859 | 0.1768 |
|  |  | rs11071591 | C/T | 0.2131 | 0.5237 | 0.1716 |
|  |  | rs7171405 | A/G | 0.5592 | 0.2788 | 0.4376 |
|  |  | rs4774388 | C/T | 0.5298 | 0.6243 | 0.5862 |
|  |  | rs4775370 | A/G | 0.6006 | 0.6390 | 0.6719 |
|  |  | rs1816624 | C/T | 0.7186 | 0.4908 | 0.9271 |
|  |  | rs4774390 | G/C | 0.7688 | 0.4363 | 0.9114 |
|  |  | rs4775371 | A/G | 0.0634 | 0.1021 | 0.0391 |
|  |  | rs1370431 | C/T | 0.4235 | 0.7498 | 0.2896 |
|  |  | rs12324086 | C/T | 0.4186 | 0.4585 | 0.2476 |
|  |  | rs17204952 | T/C | 0.1177 | 0.1302 | 0.0721 |
|  |  | rs17204959 | T/C | 0.7002 | 0.8003 | 0.7149 |
|  |  | rs12441507 | A/C | 0.8912 | 0.7762 | 0.9197 |
|  |  | rs1025676 | G/A | 0.9456 | 0.8561 | 0.9650 |
|  |  | rs55870008 | G/A | 0.6249 | 0.9153 | 0.5320 |
|  |  | rs7177846 | G/A | 0.7988 | 0.7867 | 0.5078 |
|  |  | rs2277557 | T/C | 0.6347 | 0.9803 | 0.6136 |
|  |  | rs10851691 | C/T | 0.9506 | 0.8801 | 0.9965 |
|  |  | rs10519116 | C/G | 0.3579 | 0.1927 | 0.2650 |
|  |  | rs726913 | A/G | 0.9069 | 0.7269 | 0.9531 |
|  |  | rs726955 | A/G | 0.4409 | 0.2131 | 0.3480 |
|  |  | rs2118326 | T/C | 0.8491 | 0.2930 | 0.7139 |
|  |  | rs34720147 | T/C | 0.9518 | 0.7065 | 0.7530 |
|  |  | rs4775374 | G/A | 0.8835 | 0.3015 | 0.8030 |
|  |  | rs17204973 | C/T | 0.5234 | 0.4357 | 0.4364 |
|  |  | rs1550226 | T/C | 0.9110 | 0.5400 | 0.8575 |
|  |  | rs11631786 | C/T | 0.2258 | 0.7247 | 0.1820 |
|  |  | rs11637553 | C/T | 0.7898 | 0.1688 | 0.9871 |
|  |  | rs12900971 | T/C | 0.2893 | 0.0779 | 0.6500 |
|  |  | rs146660446 | C/T | 0.4570 | 0.0232 | 0.5125 |
| *RORB* | 9 | rs17293191 | G/A | 0.9983 | 0.3116 | 0.9983 |
|  |  | rs4098048 | C/T | 0.9456 | 0.1947 | 0.9145 |
|  |  | rs13293006 | A/C | 0.0683 | 0.8194 | 0.0668 |
|  |  | rs28672222 | T/C | 0.9333 | 0.1415 | 0.8985 |
|  |  | rs1018584 | A/C | 0.4601 | 0.0229 | 0.4876 |
|  |  | rs4745330 | C/A | 0.1034 | 0.5418 | 0.1056 |
|  |  | rs7042950 | A/G | 0.0613 | 0.1292 | 0.0784 |
|  |  | rs10869418 | A/T | 0.1276 | 0.1738 | 0.1611 |
|  |  | rs17611535 | T/C | 0.2490 | 0.8918 | 0.2444 |
|  |  | rs7037043 | A/G | 0.5419 | 0.1033 | 0.6248 |
|  |  | rs75657768 | T/C | 0.3846 | 0.1156 | 0.2330 |
|  |  | rs972903 | C/T | 0.0238 | 0.0102 | 0.0437 |
|  |  | rs972902 | A/G | 0.0227 | 0.0087 | 0.0426 |
|  |  | rs17612113 | C/A | 0.3162 | 0.9921 | 0.3131 |
|  |  | rs1323354 | T/C | 0.4055 | 0.1321 | 0.3676 |
|  |  | rs62554058 | C/T | 0.1025 | 0.0478 | 0.1189 |
|  |  | rs67022110 | A/G | 0.0994 | 0.5110 | 0.1026 |
|  |  | rs10869430 | G/A | 0.2956 | 0.1619 | 0.5976 |
|  |  | rs75206074 | A/G | 0.2020 | 0.8068 | 0.2018 |
|  |  | rs1157358 | T/C | 0.3066 | 0.3184 | 0.2844 |
|  |  | rs11144029 | C/T | 0.4693 | 0.0812 | 0.6426 |
|  |  | rs3750420 | C/T | 0.2088 | 0.6739 | 0.1554 |
|  |  | rs1013078 | T/A | 0.2424 | 0.6638 | 0.2352 |
|  |  | rs10512037 | G/A | 0.0820 | 0.3089 | 0.0713 |
|  |  | rs11144032 | A/G | 0.1647 | 0.1073 | 0.4051 |
|  |  | rs2273975 | A/G | 0.7554 | 0.3641 | 0.7217 |
|  |  | rs1319551 | C/T | 0.7031 | 0.1086 | 0.7532 |
|  |  | rs10869433 | T/G | 0.6449 | 0.4219 | 0.5566 |
|  |  | rs11144039 | C/T | 0.0375 | 0.0915 | 0.0859 |
|  |  | rs72614684 | T/C | 0.0536 | 0.1321 | 0.0998 |
|  |  | rs59894901 | A/C | 0.3754 | 0.0372 | 0.2912 |
|  |  | rs10781247 | G/A | 0.8432 | 0.7019 | 0.5178 |
|  |  | rs11144045 | G/T | 0.8064 | 0.8573 | 0.7155 |
|  |  | rs7865407 | G/T | 0.6334 | 0.6976 | 0.6178 |
|  |  | rs10869435 | A/T | 0.9068 | 0.8157 | 0.6783 |
|  |  | rs10869436 | G/A | 0.8053 | 0.6784 | 0.9007 |
|  |  | rs11144053 | C/G | 0.1508 | 0.2360 | 0.1084 |
|  |  | rs3818559 | A/C | 0.7335 | 0.8269 | 0.6372 |
|  |  | rs3793517 | T/G | 0.5122 | 0.3679 | 0.6466 |
|  |  | rs10521463 | G/T | 0.3195 | 0.4120 | 0.3957 |
|  |  | rs11144064 | T/C | 0.3509 | 0.4686 | 0.4146 |
| *RORC* | 1 | rs9017 | T/C | 0.2649 | 0.7658 | 0.2344 |
|  |  | rs9826 | C/T | 0.3243 | 0.1047 | 0.4962 |
|  |  | rs3828057 | T/C | 0.3465 | 0.2804 | 0.4057 |
|  |  | rs12045886 | T/C | 0.4606 | 0.2500 | 0.8933 |
|  |  | rs1521177 | G/T | 0.9669 | 0.1003 | 0.6675 |
|  |  | rs10494269 | G/C | 0.8794 | 0.1888 | 0.9275 |
|  |  | rs7540530 | A/G | 0.8801 | 0.7383 | 0.9964 |
|  |  | rs6693413 | A/G | 0.8507 | 0.6887 | 0.9953 |
|  |  | rs11582525 | G/C | 0.2794 | 0.3456 | 0.2953 |
|  |  | rs7531041 | A/C | 0.8368 | 0.6805 | 0.9833 |
|  |  | rs72692783 | T/C | 0.9069 | 0.1538 | 0.8113 |
| *SENP3* | 17 | rs8068222 | T/G | 0.5863 | 0.3928 | 0.7041 |
|  |  | rs4602096 | C/A | 0.3387 | 0.3603 | 0.4691 |
|  |  | rs4968213 | C/T | 0.7157 | 0.2640 | 0.7514 |
|  |  | rs10468481 | A/G | 0.3802 | 0.2405 | 0.5030 |
|  |  | rs114255812 | C/T | 0.5628 | 0.8840 | 0.5628 |
| *SERPINE1* | 7 | rs2227631 | A/G | 0.4453 | 0.2380 | 0.7338 |
|  |  | rs2227672 | T/G | 0.3303 | 0.4660 | 0.3421 |
|  |  | rs2227690 | G/A | 0.2971 | 0.4968 | 0.3062 |
|  |  | rs1050955 | A/G | 0.2560 | 0.6374 | 0.1979 |
| *TIMELESS* | 12 | rs2291739 | A/G | 0.6155 | 0.7588 | 0.5072 |
|  |  | rs774048 | A/C | 0.2195 | 0.3102 | 0.2024 |
|  |  | rs774027 | T/A | 0.7745 | 0.6712 | 0.6721 |
|  |  | rs7302060 | T/C | 0.9926 | 0.4160 | 0.7236 |
|  |  | rs774045 | A/G | 0.0711 | 0.6296 | 0.0657 |
|  |  | rs3809125 | T/C | 0.9742 | 0.3012 | 0.8013 |
| *TIPIN* | 15 | rs3759786 | A/C | 0.9821 | 0.3167 | 0.9627 |
| *VIP* | 6 | rs601240 | C/G | 0.4814 | 0.9115 | 0.4716 |
|  |  | rs17083008 | A/G | 0.9523 | 0.9018 | 0.9485 |
|  |  | rs73013001 | G/A | 0.7206 | 0.9975 | 0.7188 |
|  |  | rs12212849 | G/C | 0.3207 | 0.9724 | 0.3174 |
|  |  | rs3823082 | T/C | 0.9466 | 0.9724 | 0.9452 |
|  |  | rs12201030 | G/A | 0.0312 | 0.9862 | 0.0286 |
| *VIPR2* | 7 | rs6950938 | T/C | 0.0784 | 0.5813 | 0.0773 |
|  |  | rs2730254 | C/G | 0.7658 | 0.0230 | 0.9963 |
|  |  | rs2540359 | G/A | 0.8416 | 0.0324 | 0.6109 |
|  |  | rs7784586 | C/T | 0.9546 | 0.0401 | 0.8198 |
|  |  | rs56236179 | A/G | 0.9968 | 0.0183 | 0.7360 |
|  |  | rs78219996 | A/G | 0.9304 | 0.9288 | 0.9317 |
|  |  | rs3793217 | G/A | 0.6850 | 0.2517 | 0.7562 |
|  |  | rs3793223 | A/C | 0.4452 | 0.0576 | 0.5585 |
|  |  | rs2540352 | T/C | 0.0122 | 0.0366 | 0.0175 |
|  |  | rs10263510 | G/A | 0.6753 | 0.7466 | 0.5527 |
|  |  | rs55683317 | T/C | 0.6544 | 0.1020 | 0.7743 |
|  |  | rs3793227 | G/A | 0.5625 | 0.0554 | 0.6977 |
|  |  | rs2270314 | G/C | 0.3257 | 0.0375 | 0.4230 |
|  |  | rs3793232 | C/T | 0.7277 | 0.3084 | 0.8454 |
|  |  | rs17837875 | C/T | 0.9240 | 0.0808 | 0.9257 |
|  |  | rs6974127 | T/C | 0.7437 | 0.2968 | 0.8662 |
|  |  | rs399867 | A/G | 0.0524 | 0.0223 | 0.0808 |
|  |  | rs6950857 | A/G | 0.5661 | 0.0442 | 0.6335 |
|  |  | rs3793237 | T/C | 0.5739 | 0.0639 | 0.7021 |
|  |  | rs3793239 | G/T | 0.7356 | 0.5561 | 0.8527 |
|  |  | rs74790021 | T/C | 0.8503 | 0.0543 | 0.9294 |
|  |  | rs6459928 | T/C | 0.6437 | 0.5123 | 0.6876 |
|  |  | rs3812310 | C/T | 0.0896 | 0.1935 | 0.1344 |
|  |  | rs3812311 | C/T | 0.1060 | 0.2017 | 0.1589 |
|  |  | rs3812313 | A/G | 0.5127 | 0.1245 | 0.6059 |

Chr = chromosome, MetS = metabolic syndrome.

Analysis was obtained after adjustment for covariates including age and gender.
