# Supplementary material for: Effects of circadian clock genes and health-related behavior on metabolic syndrome in a Taiwanese population: Evidence from association and interaction analysis
Source: PLoS One. 2017 Mar 15;12(3):e0173861. doi: 10.1371/journal.pone.0173861 (PMC5352001; doi:10.1371/journal.pone.0173861)
Supplement: S2 Table — (DOC) [file pone.0173861.s002.doc]

**S2 Table.** Odds ratio analysis with odds ratios after adjustment for covariates (including age and gender) between the MetS and 13 selective circadian clock genes, which have an evidence of association (P < 0.05).

| Gene | SNP | Alleles | | Additive model | | | Dominant model | | | Recessive model | | |
| --- | --- | --- | --- | --- | --- | --- | --- | --- | --- | --- | --- | --- |
|  |  |  |  | OR | 95% CI | P | OR | 95% CI | P | OR | 95% CI | P |
| *ARNTL* | rs1481892 | G | C | 0.94 | 0.82-1.07 | 0.3611 | 0.80 | 0.65-0.99 | **0.0356** | 1.04 | 0.82-1.31 | 0.7525 |
|  | rs10832020 | C | T | 1.23 | 1.05-1.45 | **0.0114** | 1.06 | 0.87-1.28 | 0.5586 | 1.54 | 1.13-2.09 | **0.0065** |
|  | rs9633835 | G | A | 0.90 | 0.78-1.03 | 0.1139 | 0.80 | 0.66-0.98 | **0.0338** | 0.92 | 0.72-1.16 | 0.4712 |
|  | rs75854041 | C | T | 1.59 | 1.06-2.39 | **0.0252** | 1.00 | 0.79-1.28 | 0.9724 | 2.55 | 1.14-5.75 | **0.0234** |
|  |  |  |  |  |  |  |  |  |  |  |  |  |
| *ARNTL2* | rs4931075 | A | G | 0.90 | 0.79-1.04 | 0.1493 | 0.79 | 0.65-0.97 | **0.0260** | 0.94 | 0.74-1.21 | 0.6396 |
|  | rs1256955 | G | T | 0.90 | 0.78-1.03 | 0.1159 | 0.80 | 0.66-0.99 | **0.0366** | 0.92 | 0.72-1.17 | 0.4816 |
|  |  |  |  |  |  |  |  |  |  |  |  |  |
| *BHLHE40* | rs9844696 | T | C | 0.83 | 0.70-0.99 | **0.0389** | 0.91 | 0.75-1.10 | 0.3454 | 0.71 | 0.51-0.98 | **0.0390** |
|  |  |  |  |  |  |  |  |  |  |  |  |  |
| *CRY1* | rs7303842 | A | G | 1.25 | 1.02-1.55 | **0.0357** | 1.25 | 1.03-1.51 | **0.0261** | 1.47 | 0.97-2.22 | 0.0701 |
|  | rs79487478 | A | G | 1.02 | 0.74-1.41 | 0.9048 | 0.79 | 0.63-0.99 | **0.0374** | 1.11 | 0.58-2.11 | 0.7603 |
|  | rs17038985 | A | G | 1.26 | 1.02-1.56 | **0.0328** | 1.25 | 1.03-1.51 | **0.0251** | 1.48 | 0.98-2.24 | 0.0652 |
|  |  |  |  |  |  |  |  |  |  |  |  |  |
| *GSK3B* | rs3732361 | G | A | 0.90 | 0.78-1.05 | 0.1723 | 1.08 | 0.88-1.33 | 0.4377 | 0.73 | 0.56-0.96 | **0.0228** |
|  | rs6782799 | C | T | 0.91 | 0.79-1.06 | 0.2347 | 1.07 | 0.88-1.31 | 0.4911 | 0.76 | 0.58-1.00 | **0.0469** |
|  | rs9878473 | T | C | 0.91 | 0.78-1.05 | 0.1950 | 1.08 | 0.88-1.32 | 0.4452 | 0.74 | 0.57-0.97 | **0.0320** |
|  | rs75126237 | T | C | 0.69 | 0.33-1.44 | 0.3281 | 1.33 | 1.04-1.71 | **0.0254** | 0.46 | 0.11-1.98 | 0.2948 |
|  | rs2199503 | T | C | 0.83 | 0.70-0.99 | **0.0331** | 1.04 | 0.85-1.26 | 0.7245 | 0.64 | 0.47-0.89 | **0.0070** |
|  |  |  |  |  |  |  |  |  |  |  |  |  |
| *HCRTR2* | rs4712099 | A | G | 0.96 | 0.79-1.16 | 0.6424 | 0.82 | 0.68-1.00 | **0.0485** | 1.00 | 0.68-1.45 | 0.9801 |
|  | rs3134711 | A | G | 1.21 | 0.98-1.49 | 0.0789 | 0.90 | 0.74-1.10 | 0.3191 | 1.55 | 1.02-2.34 | **0.0396** |
|  |  |  |  |  |  |  |  |  |  |  |  |  |
| *NPAS2* | rs72627430 | T | C | 1.03 | 0.65-1.62 | 0.9048 | 1.33 | 1.07-1.67 | **0.0120** | 0.99 | 0.40-2.46 | 0.9848 |
|  | rs77985008 | T | C | 0.69 | 0.47-1.00 | **0.0482** | 1.08 | 0.88-1.33 | 0.4466 | 0.45 | 0.22-0.95 | **0.0361** |
|  | rs13429998 | G | A | 1.85 | 1.02-3.33 | **0.0416** | 1.03 | 0.76-1.40 | 0.8541 | 3.42 | 1.05-11.14 | **0.0409** |
|  | rs6738097 | C | T | 0.80 | 0.63-1.04 | 0.0908 | 0.82 | 0.67-0.99 | **0.0432** | 0.69 | 0.42-1.14 | 0.1456 |
|  | rs12622050 | G | A | 1.18 | 1.01-1.39 | 0.0401 | 1.09 | 0.90-1.32 | 0.3814 | 1.38 | 1.02-1.88 | **0.0383** |
|  | rs2278727 | T | C | 0.86 | 0.73-1.01 | 0.0609 | 0.97 | 0.80-1.17 | 0.7368 | 0.72 | 0.53-0.98 | **0.0338** |
|  |  |  |  |  |  |  |  |  |  |  |  |  |
| *PER3* | rs228727 | T | C | 0.87 | 0.76-1.01 | 0.0655 | 0.99 | 0.80-1.21 | 0.8891 | 0.73 | 0.56-0.94 | **0.0134** |
|  | rs10746473 | G | A | 1.20 | 1.04-1.38 | **0.0116** | 1.46 | 1.17-1.83 | **0.0010** | 1.11 | 0.88-1.40 | 0.3691 |
|  | rs2797685 | C | T | 1.17 | 1.01-1.35 | **0.0314** | 1.41 | 1.11-1.78 | **0.0047** | 1.07 | 0.85-1.33 | 0.5731 |
|  | rs1689904 | C | T | 1.16 | 1.01-1.34 | **0.0352** | 1.39 | 1.10-1.76 | **0.0061** | 1.07 | 0.86-1.33 | 0.5618 |
|  | rs1773138 | T | C | 0.85 | 0.74-0.99 | **0.0304** | 0.94 | 0.76-1.17 | 0.5902 | 0.71 | 0.56-0.90 | **0.0043** |
|  | rs12563789 | G | A | 1.04 | 0.87-1.25 | 0.6475 | 1.24 | 1.02-1.50 | **0.0299** | 0.98 | 0.69-1.39 | 0.8946 |
|  |  |  |  |  |  |  |  |  |  |  |  |  |
| *REV1* | rs28745277 | C | T | 1.72 | 1.02-2.91 | **0.0429** | 1.06 | 0.79-1.42 | 0.7108 | 2.96 | 1.04-8.47 | **0.0426** |
|  |  |  |  |  |  |  |  |  |  |  |  |  |
| *RORA* | rs35277300 | C | T | 0.89 | 0.76-1.03 | 0.1039 | 0.82 | 0.68-1.00 | **0.0470** | 0.86 | 0.65-1.13 | 0.2805 |
|  | rs17237367 | A | G | 0.82 | 0.68-0.98 | **0.0263** | 0.75 | 0.62-0.91 | **0.0036** | 0.74 | 0.53-1.05 | 0.0926 |
|  | rs7168905 | G | T | 1.04 | 0.60-1.81 | 0.8878 | 1.36 | 1.07-1.73 | **0.0110** | 1.02 | 0.34-3.08 | 0.9694 |
|  | rs76194223 | T | C | 1.65 | 1.03-2.66 | **0.0385** | 1.18 | 0.90-1.54 | 0.2289 | 2.70 | 1.04-6.99 | **0.0414** |
|  | rs17303153 | G | A | 0.84 | 0.73-0.96 | **0.0140** | 0.85 | 0.70-1.05 | 0.1250 | 0.74 | 0.57-0.95 | **0.0181** |
|  | rs58469372 | A | G | 0.82 | 0.71-0.95 | **0.0092** | 0.85 | 0.70-1.04 | 0.1113 | 0.71 | 0.54-0.93 | **0.0129** |
|  | rs9302215 | C | T | 1.14 | 0.97-1.33 | 0.1042 | 1.23 | 1.02-1.50 | **0.0330** | 1.17 | 0.87-1.57 | 0.2917 |
|  | rs12591650 | A | G | 1.20 | 1.04-1.38 | **0.0109** | 1.39 | 1.11-1.75 | **0.0049** | 1.16 | 0.93-1.44 | 0.1948 |
|  | rs12594188 | C | T | 1.21 | 1.01-1.45 | **0.0389** | 1.33 | 1.10-1.61 | **0.0035** | 1.32 | 0.92-1.88 | 0.1272 |
|  | rs62002749 | A | G | 1.06 | 0.83-1.36 | 0.6452 | 1.24 | 1.02-1.51 | **0.0323** | 1.04 | 0.64-1.70 | 0.8730 |
|  | rs17270446 | G | C | 0.97 | 0.70-1.35 | 0.8455 | 1.37 | 1.12-1.68 | **0.0021** | 0.84 | 0.44-1.63 | 0.6109 |
|  | rs2899662 | T | C | 1.04 | 0.68-1.57 | 0.8705 | 1.28 | 1.04-1.59 | **0.0213** | 1.00 | 0.44-2.30 | 0.9943 |
|  | rs1680446 | T | C | 0.95 | 0.44-2.06 | 0.9054 | 1.33 | 1.02-1.75 | **0.0390** | 0.88 | 0.19-4.10 | 0.8669 |
|  | rs877228 | G | A | 0.91 | 0.78-1.05 | 0.1977 | 1.06 | 0.87-1.30 | 0.5416 | 0.75 | 0.57-0.99 | 0.0456 |
|  | rs7162615 | A | G | 1.27 | 1.03-1.57 | **0.0248** | 1.12 | 0.93-1.37 | 0.2373 | 1.58 | 1.05-2.40 | **0.0299** |
|  | rs10519076 | C | G | 1.03 | 0.83-1.29 | 0.7654 | 0.80 | 0.66-0.98 | **0.0293** | 1.18 | 0.76-1.83 | 0.4645 |
|  | rs10152719 | T | C | 1.04 | 0.83-1.30 | 0.7487 | 0.82 | 0.68-1.00 | **0.0473** | 1.18 | 0.76-1.82 | 0.4727 |
|  | rs12439995 | G | C | 1.00 | 0.82-1.22 | 0.9858 | 0.81 | 0.67-0.98 | **0.0296** | 1.10 | 0.74-1.64 | 0.6326 |
|  | rs11630062 | C | T | 0.92 | 0.80-1.05 | 0.2267 | 0.76 | 0.62-0.93 | **0.0082** | 1.02 | 0.80-1.30 | 0.8834 |
|  | rs12910281 | C | T | 1.03 | 0.89-1.19 | 0.7346 | 1.27 | 1.02-1.57 | **0.0328** | 0.86 | 0.67-1.11 | 0.2407 |
|  | rs28705880 | T | G | 0.84 | 0.72-0.98 | **0.0238** | 0.84 | 0.69-1.03 | 0.0885 | 0.76 | 0.57-1.00 | **0.0483** |
|  | rs72752780 | C | A | 0.77 | 0.60-1.00 | **0.0457** | 0.92 | 0.76-1.12 | 0.4306 | 0.60 | 0.36-0.99 | **0.0471** |
|  | rs8029848 | G | A | 1.28 | 1.05-1.56 | **0.0127** | 1.41 | 1.17-1.71 | **0.0004** | 1.43 | 0.98-2.09 | 0.0629 |
|  | rs8034880 | G | A | 1.29 | 1.05-1.58 | **0.0150** | 1.44 | 1.19-1.74 | **0.0002** | 1.44 | 0.97-2.14 | 0.0711 |
|  | rs8034950 | C | T | 1.17 | 1.01-1.34 | **0.0341** | 1.23 | 1.00-1.51 | **0.0466** | 1.23 | 0.95-1.58 | 0.1144 |
|  | rs4775340 | A | G | 0.94 | 0.69-1.28 | 0.7009 | 0.78 | 0.63-0.97 | **0.0271** | 0.95 | 0.51-1.75 | 0.8594 |
|  | rs17237521 | T | C | 0.84 | 0.72-0.98 | **0.0234** | 0.89 | 0.73-1.08 | 0.2443 | 0.72 | 0.55-0.96 | **0.0228** |
|  | rs72752802 | C | A | 0.81 | 0.69-0.94 | **0.0053** | 0.87 | 0.72-1.06 | 0.1716 | 0.67 | 0.50-0.88 | **0.0045** |
|  | rs11631656 | G | A | 0.43 | 0.21-0.87 | **0.0186** | 0.92 | 0.74-1.15 | 0.4531 | 0.18 | 0.04-0.75 | **0.0188** |
|  | rs1467304 | C | T | 0.52 | 0.29-0.94 | **0.0294** | 0.91 | 0.73-1.13 | 0.3874 | 0.27 | 0.08-0.88 | **0.0305** |
|  | rs62005615 | A | C | 0.75 | 0.36-1.57 | 0.4473 | 0.74 | 0.55-0.99 | **0.0459** | 0.58 | 0.13-2.56 | 0.4756 |
|  | rs72625740 | C | T | 1.24 | 1.01-1.52 | **0.0407** | 1.04 | 0.86-1.27 | 0.6828 | 1.55 | 1.03-2.32 | **0.0344** |
|  | rs72625742 | T | C | 1.22 | 1.03-1.45 | **0.0208** | 1.04 | 0.86-1.26 | 0.6951 | 1.52 | 1.09-2.10 | **0.0127** |
|  | rs1403739 | G | A | 1.19 | 1.01-1.41 | **0.0437** | 1.04 | 0.86-1.26 | 0.7018 | 1.44 | 1.04-2.00 | **0.0305** |
|  | rs6494243 | G | A | 0.60 | 0.38-0.96 | **0.0327** | 0.84 | 0.67-1.06 | 0.1430 | 0.37 | 0.15-0.94 | **0.0368** |
|  | rs782948 | A | G | 0.59 | 0.35-0.98 | **0.0432** | 0.89 | 0.71-1.12 | 0.3314 | 0.35 | 0.12-0.98 | **0.0453** |
|  | rs7173460 | A | G | 1.46 | 1.03-2.08 | **0.0339** | 0.92 | 0.73-1.17 | 0.4917 | 2.21 | 1.09-4.46 | **0.0273** |
|  | rs77786240 | A | G | 1.63 | 1.01-2.62 | **0.0435** | 1.20 | 0.92-1.57 | 0.1736 | 2.61 | 1.01-6.73 | **0.0475** |
|  | rs13329643 | T | C | 1.43 | 1.02-1.99 | **0.0356** | 1.07 | 0.86-1.33 | 0.5597 | 2.03 | 1.05-3.92 | **0.0359** |
|  | rs8024672 | A | C | 1.06 | 0.73-1.55 | 0.7452 | 0.78 | 0.61-1.00 | **0.0455** | 1.19 | 0.56-2.53 | 0.6424 |
|  | rs17303530 | G | T | 1.31 | 0.99-1.73 | 0.0583 | 0.94 | 0.76-1.16 | 0.5478 | 1.77 | 1.02-3.08 | **0.0432** |
|  | rs4775371 | A | G | 1.32 | 0.98-1.76 | 0.0634 | 0.83 | 0.66-1.04 | 0.1021 | 1.84 | 1.03-3.29 | **0.0391** |
|  | rs146660446 | C | T | 0.82 | 0.48-1.40 | 0.4570 | 0.74 | 0.57-0.96 | **0.0232** | 0.70 | 0.24-2.04 | 0.5125 |
|  |  |  |  |  |  |  |  |  |  |  |  |  |
| *RORB* | rs1018584 | A | C | 0.68 | 0.24-1.90 | 0.4601 | 0.73 | 0.55-0.96 | **0.0229** | 0.48 | 0.06-3.80 | 0.4876 |
|  | rs972903 | C | T | 0.73 | 0.55-0.96 | **0.0238** | 0.77 | 0.63-0.94 | **0.0102** | 0.57 | 0.33-0.98 | **0.0437** |
|  | rs972902 | A | G | 0.72 | 0.55-0.96 | **0.0227** | 0.76 | 0.62-0.93 | **0.0087** | 0.57 | 0.33-0.98 | **0.0426** |
|  | rs62554058 | C | T | 0.61 | 0.34-1.10 | 0.1025 | 0.79 | 0.63-1.00 | **0.0478** | 0.39 | 0.12-1.28 | 0.1189 |
|  | rs11144039 | C | T | 1.15 | 1.01-1.32 | **0.0375** | 1.20 | 0.97-1.49 | 0.0915 | 1.22 | 0.97-1.53 | 0.0859 |
|  | rs59894901 | A | C | 1.18 | 0.82-1.70 | 0.3754 | 0.78 | 0.62-0.99 | **0.0372** | 1.48 | 0.71-3.08 | 0.2912 |
|  |  |  |  |  |  |  |  |  |  |  |  |  |
| *VIP* | rs12201030 | G | A | 1.51 | 1.04-2.21 | **0.0312** | 1.00 | 0.79-1.25 | 0.9862 | 2.32 | 1.09-4.92 | **0.0286** |
|  |  |  |  |  |  |  |  |  |  |  |  |  |
| *VIPR2* | rs2730254 | C | G | 0.96 | 0.74-1.24 | 0.7658 | 0.79 | 0.64-0.97 | **0.0230** | 1.00 | 0.60-1.67 | 0.9963 |
|  | rs2540359 | G | A | 1.03 | 0.80-1.32 | 0.8416 | 0.80 | 0.65-0.98 | **0.0324** | 1.14 | 0.69-1.89 | 0.6109 |
|  | rs7784586 | C | T | 0.99 | 0.77-1.28 | 0.9546 | 0.81 | 0.66-0.99 | **0.0401** | 1.06 | 0.64-1.75 | 0.8198 |
|  | rs56236179 | A | G | 1.00 | 0.78-1.29 | 0.9968 | 0.78 | 0.64-0.96 | **0.0183** | 1.09 | 0.66-1.80 | 0.7360 |
|  | rs2540352 | T | C | 0.64 | 0.45-0.91 | **0.0122** | 0.80 | 0.65-0.99 | **0.0366** | 0.43 | 0.21-0.86 | **0.0175** |
|  | rs2270314 | G | C | 0.85 | 0.63-1.17 | 0.3257 | 0.80 | 0.64-0.99 | **0.0375** | 0.78 | 0.42-1.45 | 0.4230 |
|  | rs399867 | A | G | 0.75 | 0.56-1.00 | 0.0524 | 0.78 | 0.64-0.97 | **0.0223** | 0.60 | 0.34-1.07 | 0.0808 |
|  | rs6950857 | A | G | 1.14 | 0.74-1.75 | 0.5661 | 1.28 | 1.01-1.63 | **0.0442** | 1.24 | 0.52-2.94 | 0.6335 |

CI = confidence interval, MetS = metabolic syndrome, OR = odds ratio.

Analysis was obtained after adjustment for covariates including age and gender. P values of < 0.05 are shown in bold.
