# Supplementary material for: Effects of circadian clock genes and health-related behavior on metabolic syndrome in a Taiwanese population: Evidence from association and interaction analysis
Source: PLoS One. 2017 Mar 15;12(3):e0173861. doi: 10.1371/journal.pone.0173861 (PMC5352001; doi:10.1371/journal.pone.0173861)
Supplement: S3 Table — (DOC) [file pone.0173861.s003.doc]

**S3 Table.** Q values and FDRs for odds ratio analysis between the MetS and 881 SNPs in 29 circadian clock genes.

| Gene | CHR | SNP | Q (Additive) | FDR (Additive) | Q (Dominant) | FDR (Dominant) | Q (Recessive) | FDR (Recessive) |
| --- | --- | --- | --- | --- | --- | --- | --- | --- |
| *ADCYAP1* | 18 | rs1893154 | 0.9761 | 1.0000 | 0.9986 | 1.0000 | 0.9880 | 1.0000 |
|  |  | rs8086678 | 0.9705 | 1.0000 | 0.9874 | 1.0000 | 0.9609 | 1.0000 |
|  |  | rs928980 | 0.9502 | 1.0000 | 0.8260 | 0.9863 | 1.0000 | 1.0000 |
|  |  | rs2231187 | 1.0000 | 1.0000 | 0.9874 | 1.0000 | 1.0000 | 1.0000 |
|  |  | rs1610037 | 1.0000 | 1.0000 | 0.9671 | 1.0000 | 1.0000 | 1.0000 |
| *ARNTL* | 11 | rs1481892 | 0.9502 | 1.0000 | 0.7844 | 0.7360 | 1.0000 | 1.0000 |
|  |  | rs7950226 | 1.0000 | 1.0000 | 0.9700 | 1.0000 | 1.0000 | 1.0000 |
|  |  | rs7951393 | 0.9927 | 1.0000 | 0.7844 | 0.8063 | 0.9985 | 1.0000 |
|  |  | rs72867496 | 0.9705 | 1.0000 | 0.9084 | 1.0000 | 0.9880 | 1.0000 |
|  |  | rs10832020 | 0.8604 | 0.9013 | 0.9671 | 1.0000 | 0.8722 | 0.9861 |
|  |  | rs6486120 | 0.9743 | 1.0000 | 0.9700 | 1.0000 | 0.8881 | 0.9861 |
|  |  | rs11022761 | 0.9502 | 1.0000 | 0.9224 | 1.0000 | 0.9609 | 1.0000 |
|  |  | rs4757143 | 0.9502 | 1.0000 | 0.8587 | 1.0000 | 0.9546 | 1.0000 |
|  |  | rs10741616 | 0.9502 | 1.0000 | 0.8015 | 0.8495 | 0.9880 | 1.0000 |
|  |  | rs11022762 | 0.9705 | 1.0000 | 0.9493 | 1.0000 | 1.0000 | 1.0000 |
|  |  | rs10766076 | 1.0000 | 1.0000 | 0.9634 | 1.0000 | 0.9880 | 1.0000 |
|  |  | rs12805304 | 0.8604 | 0.9689 | 0.9835 | 1.0000 | 0.8722 | 0.9861 |
|  |  | rs9633835 | 0.8604 | 0.9573 | 0.7844 | 0.7360 | 0.9880 | 1.0000 |
|  |  | rs34188368 | 0.9502 | 1.0000 | 0.9854 | 1.0000 | 0.9609 | 1.0000 |
|  |  | rs28711392 | 1.0000 | 1.0000 | 0.8015 | 0.9225 | 0.9972 | 1.0000 |
|  |  | rs10766077 | 1.0000 | 1.0000 | 0.8353 | 0.9990 | 0.9972 | 1.0000 |
|  |  | rs7924734 | 0.9502 | 1.0000 | 0.9874 | 1.0000 | 0.9540 | 0.9953 |
|  |  | rs11022769 | 0.9705 | 1.0000 | 0.9863 | 1.0000 | 0.9546 | 1.0000 |
|  |  | rs16912743 | 0.9502 | 1.0000 | 0.9594 | 1.0000 | 0.9546 | 1.0000 |
|  |  | rs6486121 | 1.0000 | 1.0000 | 0.9380 | 1.0000 | 1.0000 | 1.0000 |
|  |  | rs7947951 | 1.0000 | 1.0000 | 0.9986 | 1.0000 | 1.0000 | 1.0000 |
|  |  | rs16912751 | 0.9705 | 1.0000 | 0.9429 | 1.0000 | 0.9972 | 1.0000 |
|  |  | rs1026071 | 1.0000 | 1.0000 | 0.8587 | 1.0000 | 1.0000 | 1.0000 |
|  |  | rs34834014 | 1.0000 | 1.0000 | 0.9612 | 1.0000 | 1.0000 | 1.0000 |
|  |  | rs11022775 | 1.0000 | 1.0000 | 0.9594 | 1.0000 | 1.0000 | 1.0000 |
|  |  | rs34991502 | 0.9502 | 1.0000 | 0.9874 | 1.0000 | 0.9609 | 1.0000 |
|  |  | rs1868049 | 0.9502 | 1.0000 | 0.9594 | 1.0000 | 0.9546 | 1.0000 |
|  |  | rs3789327 | 0.9502 | 1.0000 | 0.9863 | 1.0000 | 0.9609 | 1.0000 |
|  |  | rs11022778 | 1.0000 | 1.0000 | 0.9787 | 1.0000 | 1.0000 | 1.0000 |
|  |  | rs4757151 | 1.0000 | 1.0000 | 0.9833 | 1.0000 | 1.0000 | 1.0000 |
|  |  | rs75854041 | 0.8604 | 0.9013 | 0.9986 | 1.0000 | 0.8722 | 0.9861 |
|  |  | rs74762146 | 1.0000 | 1.0000 | 0.9491 | 1.0000 | 1.0000 | 1.0000 |
|  |  | rs72869173 | 0.9502 | 1.0000 | 0.9863 | 1.0000 | 0.9609 | 1.0000 |
|  |  | rs11022779 | 1.0000 | 1.0000 | 0.9594 | 1.0000 | 1.0000 | 1.0000 |
|  |  | rs969485 | 0.9502 | 1.0000 | 0.9787 | 1.0000 | 0.9609 | 1.0000 |
| *ARNTL2* | 12 | rs7137588 | 0.9502 | 1.0000 | 0.8015 | 0.9191 | 0.9609 | 1.0000 |
|  |  | rs4964052 | 0.9502 | 1.0000 | 0.9612 | 1.0000 | 0.9546 | 1.0000 |
|  |  | rs16931885 | 1.0000 | 1.0000 | 0.8410 | 1.0000 | 1.0000 | 1.0000 |
|  |  | rs17497606 | 0.9705 | 1.0000 | 0.8444 | 1.0000 | 0.9880 | 1.0000 |
|  |  | rs16931888 | 1.0000 | 1.0000 | 0.8361 | 1.0000 | 1.0000 | 1.0000 |
|  |  | rs61915952 | 1.0000 | 1.0000 | 0.8444 | 1.0000 | 1.0000 | 1.0000 |
|  |  | rs11048977 | 1.0000 | 1.0000 | 0.9700 | 1.0000 | 1.0000 | 1.0000 |
|  |  | rs1037924 | 1.0000 | 1.0000 | 0.9986 | 1.0000 | 0.9972 | 1.0000 |
|  |  | rs17497857 | 1.0000 | 1.0000 | 0.8343 | 0.9935 | 1.0000 | 1.0000 |
|  |  | rs2968756 | 1.0000 | 1.0000 | 0.9380 | 1.0000 | 1.0000 | 1.0000 |
|  |  | rs10771339 | 1.0000 | 1.0000 | 0.9594 | 1.0000 | 0.9880 | 1.0000 |
|  |  | rs2927030 | 1.0000 | 1.0000 | 0.8256 | 0.9726 | 1.0000 | 1.0000 |
|  |  | rs3751220 | 1.0000 | 1.0000 | 0.7844 | 0.8111 | 1.0000 | 1.0000 |
|  |  | rs4964059 | 0.9705 | 1.0000 | 0.8015 | 0.9278 | 1.0000 | 1.0000 |
|  |  | rs11048994 | 0.9705 | 1.0000 | 0.9095 | 1.0000 | 0.9973 | 1.0000 |
|  |  | rs11048995 | 0.9502 | 1.0000 | 0.8444 | 1.0000 | 0.9609 | 1.0000 |
|  |  | rs4964060 | 0.9502 | 1.0000 | 0.9456 | 1.0000 | 0.9609 | 1.0000 |
|  |  | rs12300289 | 0.9502 | 1.0000 | 0.8015 | 0.8960 | 0.9609 | 1.0000 |
|  |  | rs3751221 | 0.9502 | 1.0000 | 0.8015 | 0.8860 | 0.9546 | 1.0000 |
|  |  | rs16931937 | 0.9502 | 1.0000 | 0.8015 | 0.8943 | 0.9660 | 1.0000 |
|  |  | rs16931939 | 0.9502 | 1.0000 | 0.8015 | 0.8839 | 0.9609 | 1.0000 |
|  |  | rs4931075 | 0.9502 | 1.0000 | 0.7844 | 0.7360 | 1.0000 | 1.0000 |
|  |  | rs813535 | 1.0000 | 1.0000 | 0.8256 | 0.9790 | 0.9972 | 1.0000 |
|  |  | rs1256955 | 0.8604 | 0.9607 | 0.7844 | 0.7360 | 0.9880 | 1.0000 |
|  |  | rs10842913 | 0.9502 | 1.0000 | 0.8256 | 0.9762 | 0.9609 | 1.0000 |
|  |  | rs2682706 | 0.8604 | 0.9546 | 0.7844 | 0.7360 | 0.9972 | 1.0000 |
|  |  | rs73079936 | 0.9502 | 1.0000 | 0.7844 | 0.7992 | 0.9609 | 1.0000 |
|  |  | rs11049010 | 0.8604 | 0.9608 | 0.8015 | 0.8888 | 0.9547 | 1.0000 |
|  |  | rs1443859 | 0.9502 | 1.0000 | 0.9671 | 1.0000 | 0.9609 | 1.0000 |
|  |  | rs683813 | 0.9705 | 1.0000 | 0.8256 | 0.9833 | 0.9972 | 1.0000 |
| *BHLHE40* | 3 | rs58358486 | 1.0000 | 1.0000 | 0.9854 | 1.0000 | 0.9972 | 1.0000 |
|  |  | rs6808127 | 0.9502 | 1.0000 | 0.9986 | 1.0000 | 0.8881 | 0.9861 |
|  |  | rs6775761 | 1.0000 | 1.0000 | 0.9787 | 1.0000 | 0.9609 | 1.0000 |
|  |  | rs9844696 | 0.8604 | 0.9013 | 0.8976 | 1.0000 | 0.8722 | 0.9861 |
|  |  | rs9875607 | 1.0000 | 1.0000 | 0.8444 | 1.0000 | 1.0000 | 1.0000 |
|  |  | rs728390 | 0.9812 | 1.0000 | 0.9863 | 1.0000 | 0.9742 | 1.0000 |
|  |  | rs728389 | 0.9705 | 1.0000 | 0.9874 | 1.0000 | 0.9609 | 1.0000 |
|  |  | rs17041730 | 1.0000 | 1.0000 | 0.8677 | 1.0000 | 1.0000 | 1.0000 |
|  |  | rs17041733 | 1.0000 | 1.0000 | 0.9787 | 1.0000 | 1.0000 | 1.0000 |
|  |  | rs1514451 | 1.0000 | 1.0000 | 0.8015 | 0.9202 | 0.9609 | 1.0000 |
|  |  | rs59218436 | 1.0000 | 1.0000 | 0.8015 | 0.9197 | 1.0000 | 1.0000 |
|  |  | rs13069852 | 0.9705 | 1.0000 | 0.9671 | 1.0000 | 0.9880 | 1.0000 |
|  |  | rs79878432 | 1.0000 | 1.0000 | 0.8444 | 1.0000 | 1.0000 | 1.0000 |
|  |  | rs4402927 | 1.0000 | 1.0000 | 0.9594 | 1.0000 | 1.0000 | 1.0000 |
|  |  | rs7629118 | 1.0000 | 1.0000 | 0.9793 | 1.0000 | 1.0000 | 1.0000 |
|  |  | rs9990146 | 0.8604 | 0.9013 | 0.7844 | 0.7360 | 0.9609 | 1.0000 |
|  |  | rs9990172 | 0.9502 | 1.0000 | 0.9787 | 1.0000 | 0.9609 | 1.0000 |
|  |  | rs6442920 | 1.0000 | 1.0000 | 0.9671 | 1.0000 | 1.0000 | 1.0000 |
|  |  | rs6764243 | 0.9502 | 1.0000 | 0.9787 | 1.0000 | 0.9609 | 1.0000 |
|  |  | rs75888088 | 0.9502 | 1.0000 | 0.9787 | 1.0000 | 0.9540 | 1.0000 |
|  |  | rs9311438 | 0.9705 | 1.0000 | 0.9671 | 1.0000 | 0.9742 | 1.0000 |
|  |  | rs79740996 | 1.0000 | 1.0000 | 0.9594 | 1.0000 | 1.0000 | 1.0000 |
|  |  | rs9836120 | 0.9502 | 1.0000 | 0.7856 | 0.8313 | 0.9880 | 1.0000 |
|  |  | rs72997163 | 0.9993 | 1.0000 | 0.9771 | 1.0000 | 1.0000 | 1.0000 |
|  |  | rs13065754 | 0.9743 | 1.0000 | 0.9986 | 1.0000 | 0.9609 | 1.0000 |
|  |  | rs7635516 | 0.9502 | 1.0000 | 0.9671 | 1.0000 | 0.9546 | 1.0000 |
|  |  | rs7638383 | 1.0000 | 1.0000 | 0.9671 | 1.0000 | 1.0000 | 1.0000 |
|  |  | rs63538861 | 1.0000 | 1.0000 | 0.8444 | 1.0000 | 0.9973 | 1.0000 |
|  |  | rs3846155 | 1.0000 | 1.0000 | 0.8444 | 1.0000 | 1.0000 | 1.0000 |
|  |  | rs7640124 | 1.0000 | 1.0000 | 0.9793 | 1.0000 | 1.0000 | 1.0000 |
|  |  | rs6442925 | 0.9705 | 1.0000 | 0.9594 | 1.0000 | 0.9880 | 1.0000 |
|  |  | rs1588794 | 1.0000 | 1.0000 | 0.7844 | 0.8192 | 1.0000 | 1.0000 |
|  |  | rs6764396 | 1.0000 | 1.0000 | 0.9671 | 1.0000 | 1.0000 | 1.0000 |
|  |  | rs1110261 | 1.0000 | 1.0000 | 0.9863 | 1.0000 | 1.0000 | 1.0000 |
|  |  | rs1104976 | 1.0000 | 1.0000 | 0.9403 | 1.0000 | 1.0000 | 1.0000 |
| *CLOCK* | 4 | rs3749473 | 1.0000 | 1.0000 | 0.9693 | 1.0000 | 1.0000 | 1.0000 |
|  |  | rs6832769 | 1.0000 | 1.0000 | 0.9793 | 1.0000 | 1.0000 | 1.0000 |
|  |  | rs11932595 | 1.0000 | 1.0000 | 0.9806 | 1.0000 | 1.0000 | 1.0000 |
|  |  | rs12642716 | 1.0000 | 1.0000 | 0.9980 | 1.0000 | 1.0000 | 1.0000 |
|  |  | rs62303728 | 1.0000 | 1.0000 | 0.9874 | 1.0000 | 1.0000 | 1.0000 |
|  |  | rs11133391 | 1.0000 | 1.0000 | 0.9863 | 1.0000 | 1.0000 | 1.0000 |
|  |  | rs7673908 | 1.0000 | 1.0000 | 0.9874 | 1.0000 | 1.0000 | 1.0000 |
|  |  | rs117876670 | NA | NA | 0.7844 | 0.8009 | NA | NA |
| *CRY1* | 12 | rs7303842 | 0.8604 | 0.9013 | 0.7844 | 0.7360 | 0.8722 | 0.9861 |
|  |  | rs79487478 | 1.0000 | 1.0000 | 0.7844 | 0.7360 | 1.0000 | 1.0000 |
|  |  | rs11113179 | 0.9502 | 1.0000 | 0.9863 | 1.0000 | 0.9609 | 1.0000 |
|  |  | rs17038985 | 0.8604 | 0.9013 | 0.7844 | 0.7360 | 0.8722 | 0.9861 |
| *CRY2* | 11 | rs10838524 | 0.8604 | 0.9510 | 0.9986 | 1.0000 | 0.8722 | 0.9861 |
|  |  | rs11605924 | 0.8604 | 0.9077 | 0.9986 | 1.0000 | 0.8722 | 0.9861 |
|  |  | rs4756034 | 0.8604 | 0.9228 | 0.8353 | 1.0000 | 0.9374 | 0.9902 |
|  |  | rs4756035 | 0.8604 | 0.9304 | 0.8353 | 1.0000 | 0.9540 | 1.0000 |
|  |  | rs2292912 | 0.8604 | 0.9013 | 0.8256 | 0.9725 | 0.9030 | 0.9861 |
|  |  | rs11038699 | 0.9502 | 1.0000 | 0.8064 | 0.9546 | 0.9609 | 1.0000 |
|  |  | rs2292910 | 0.9502 | 1.0000 | 0.9766 | 1.0000 | 0.9609 | 1.0000 |
| *CSNK1D* | 17 | rs116950947 | 1.0000 | 1.0000 | 0.9594 | 1.0000 | 1.0000 | 1.0000 |
|  |  | rs3829773 | 1.0000 | 1.0000 | 0.9793 | 1.0000 | 1.0000 | 1.0000 |
|  |  | rs4789846 | 0.9705 | 1.0000 | 0.8587 | 1.0000 | 0.9972 | 1.0000 |
| *CSNK1E* | 22 | rs5750581 | 0.9502 | 1.0000 | 0.9986 | 1.0000 | 0.9609 | 1.0000 |
|  |  | rs135763 | 0.9502 | 1.0000 | 0.9986 | 1.0000 | 0.9609 | 1.0000 |
|  |  | rs1997644 | 0.9502 | 1.0000 | 0.9700 | 1.0000 | 0.9355 | 0.9861 |
|  |  | rs3747169 | 1.0000 | 1.0000 | 0.8015 | 0.8982 | 1.0000 | 1.0000 |
|  |  | rs195314 | 0.9502 | 1.0000 | 0.9084 | 1.0000 | 0.8881 | 0.9861 |
|  |  | rs17753544 | 1.0000 | 1.0000 | 0.8015 | 0.8891 | 1.0000 | 1.0000 |
|  |  | rs55719829 | 1.0000 | 1.0000 | 0.8015 | 0.8853 | 1.0000 | 1.0000 |
|  |  | rs196105 | 1.0000 | 1.0000 | 0.7844 | 0.7753 | 1.0000 | 1.0000 |
|  |  | rs138366 | 0.9502 | 1.0000 | 0.9456 | 1.0000 | 0.9546 | 1.0000 |
|  |  | rs138369 | 0.9502 | 1.0000 | 0.9392 | 1.0000 | 0.9842 | 1.0000 |
| *GSK3B* | 3 | rs3732361 | 0.9502 | 1.0000 | 0.9493 | 1.0000 | 0.8722 | 0.9861 |
|  |  | rs2873950 | 0.9705 | 1.0000 | 0.9890 | 1.0000 | 0.9546 | 1.0000 |
|  |  | rs10934500 | 0.9502 | 1.0000 | 0.7844 | 0.8184 | 1.0000 | 1.0000 |
|  |  | rs1719888 | 1.0000 | 1.0000 | 0.9693 | 1.0000 | 1.0000 | 1.0000 |
|  |  | rs45567135 | 0.9502 | 1.0000 | 0.7844 | 0.7879 | 0.9609 | 1.0000 |
|  |  | rs16830594 | 1.0000 | 1.0000 | 0.8375 | 1.0000 | 0.9609 | 1.0000 |
|  |  | rs6795653 | 0.9705 | 1.0000 | 0.9538 | 1.0000 | 0.9374 | 0.9861 |
|  |  | rs6782799 | 0.9502 | 1.0000 | 0.9594 | 1.0000 | 0.8722 | 0.9861 |
|  |  | rs79646574 | 1.0000 | 1.0000 | 0.9671 | 1.0000 | 1.0000 | 1.0000 |
|  |  | rs4340737 | 0.9502 | 1.0000 | 0.8015 | 0.8730 | 1.0000 | 1.0000 |
|  |  | rs78268305 | 1.0000 | 1.0000 | 0.9634 | 1.0000 | 1.0000 | 1.0000 |
|  |  | rs13314595 | 0.9743 | 1.0000 | 0.9594 | 1.0000 | 0.9880 | 1.0000 |
|  |  | rs7431209 | 0.8604 | 0.9013 | 0.9793 | 1.0000 | 0.8722 | 0.9861 |
|  |  | rs9878473 | 0.9502 | 1.0000 | 0.9504 | 1.0000 | 0.8722 | 0.9861 |
|  |  | rs76950779 | 1.0000 | 1.0000 | 0.7844 | 0.7719 | 1.0000 | 1.0000 |
|  |  | rs6807868 | 1.0000 | 1.0000 | 0.9763 | 1.0000 | 1.0000 | 1.0000 |
|  |  | rs12054090 | 0.9705 | 1.0000 | 0.8015 | 0.9086 | 1.0000 | 1.0000 |
|  |  | rs12638973 | 0.9502 | 1.0000 | 0.8015 | 0.8442 | 1.0000 | 1.0000 |
|  |  | rs76330913 | 1.0000 | 1.0000 | 0.9671 | 1.0000 | 1.0000 | 1.0000 |
|  |  | rs75008340 | 1.0000 | 1.0000 | 0.9224 | 1.0000 | 1.0000 | 1.0000 |
|  |  | rs10934506 | 0.9705 | 1.0000 | 0.8256 | 0.9664 | 1.0000 | 1.0000 |
|  |  | rs968824 | 1.0000 | 1.0000 | 0.9671 | 1.0000 | 1.0000 | 1.0000 |
|  |  | rs75126237 | 0.9502 | 1.0000 | 0.7844 | 0.7360 | 0.9609 | 1.0000 |
|  |  | rs2199503 | 0.8604 | 0.9013 | 0.9793 | 1.0000 | 0.8722 | 0.9861 |
|  |  | rs334535 | 1.0000 | 1.0000 | 0.9392 | 1.0000 | 0.9972 | 1.0000 |
|  |  | rs334559 | 1.0000 | 1.0000 | 0.9594 | 1.0000 | 0.9973 | 1.0000 |
| [*HCRTR2*](mailto:HCRTR@) | 6 | rs10456181 | 0.9705 | 1.0000 | 0.8444 | 1.0000 | 0.9880 | 1.0000 |
|  |  | rs4364482 | 0.8604 | 0.9028 | 0.8587 | 1.0000 | 0.8722 | 0.9861 |
|  |  | rs6927478 | 1.0000 | 1.0000 | 0.9986 | 1.0000 | 1.0000 | 1.0000 |
|  |  | rs3134689 | 1.0000 | 1.0000 | 0.9986 | 1.0000 | 0.9976 | 1.0000 |
|  |  | rs3122152 | 1.0000 | 1.0000 | 0.9874 | 1.0000 | 1.0000 | 1.0000 |
|  |  | rs4712099 | 1.0000 | 1.0000 | 0.7844 | 0.7467 | 1.0000 | 1.0000 |
|  |  | rs9475195 | 0.9502 | 1.0000 | 0.9538 | 1.0000 | 0.9546 | 1.0000 |
|  |  | rs12526414 | 1.0000 | 1.0000 | 0.9926 | 1.0000 | 0.9972 | 1.0000 |
|  |  | rs3134704 | 0.9502 | 1.0000 | 0.9771 | 1.0000 | 0.9026 | 0.9861 |
|  |  | rs3134705 | 0.9502 | 1.0000 | 0.9527 | 1.0000 | 0.9546 | 1.0000 |
|  |  | rs9370402 | NA | NA | 0.9700 | 1.0000 | NA | NA |
|  |  | rs3122155 | 0.9502 | 1.0000 | 0.8976 | 1.0000 | 0.9546 | 1.0000 |
|  |  | rs3122156 | 0.9502 | 1.0000 | 0.9627 | 1.0000 | 0.9609 | 1.0000 |
|  |  | rs3122160 | 0.9705 | 1.0000 | 0.9671 | 1.0000 | 0.9679 | 1.0000 |
|  |  | rs3122162 | 0.9502 | 1.0000 | 0.8444 | 1.0000 | 0.9026 | 0.9861 |
|  |  | rs60748030 | 1.0000 | 1.0000 | 0.7844 | 0.7859 | 0.9609 | 1.0000 |
|  |  | rs3134711 | 0.8604 | 0.9013 | 0.8587 | 1.0000 | 0.8722 | 0.9861 |
|  |  | rs7741664 | 0.8604 | 0.9013 | 0.9854 | 1.0000 | 0.8722 | 0.9861 |
|  |  | rs12525016 | 0.9544 | 1.0000 | 0.9594 | 1.0000 | 0.9609 | 1.0000 |
|  |  | rs3122167 | 0.9050 | 0.9856 | 0.8983 | 1.0000 | 0.8722 | 0.9861 |
|  |  | rs9396073 | 0.9075 | 0.9898 | 0.9854 | 1.0000 | 0.9030 | 0.9861 |
|  |  | rs2811239 | 0.9502 | 1.0000 | 0.9787 | 1.0000 | 0.8722 | 0.9861 |
|  |  | rs74937200 | 1.0000 | 1.0000 | 0.9456 | 1.0000 | 1.0000 | 1.0000 |
|  |  | rs2653342 | 1.0000 | 1.0000 | 0.9890 | 1.0000 | 1.0000 | 1.0000 |
|  |  | rs2653344 | 1.0000 | 1.0000 | 0.9874 | 1.0000 | 0.9972 | 1.0000 |
|  |  | rs7452651 | 1.0000 | 1.0000 | 0.9863 | 1.0000 | 1.0000 | 1.0000 |
|  |  | rs2653346 | 1.0000 | 1.0000 | 0.9863 | 1.0000 | 0.9976 | 1.0000 |
|  |  | rs2811241 | 1.0000 | 1.0000 | 0.9771 | 1.0000 | 0.9880 | 1.0000 |
|  |  | rs2653350 | 1.0000 | 1.0000 | 0.9693 | 1.0000 | 1.0000 | 1.0000 |
|  |  | rs74296544 | 0.9705 | 1.0000 | 0.9634 | 1.0000 | 0.9880 | 1.0000 |
|  |  | rs3800543 | 1.0000 | 1.0000 | 0.9986 | 1.0000 | 1.0000 | 1.0000 |
|  |  | rs76380807 | 1.0000 | 1.0000 | 0.9863 | 1.0000 | 1.0000 | 1.0000 |
| *KLF10* | 8 | rs3191333 | 1.0000 | 1.0000 | 0.9932 | 1.0000 | 1.0000 | 1.0000 |
|  |  | rs11552577 | 1.0000 | 1.0000 | 0.9771 | 1.0000 | 1.0000 | 1.0000 |
| *NFIL3* | 9 | rs80064239 | 0.9790 | 1.0000 | 0.7856 | 0.8258 | 0.9880 | 1.0000 |
|  |  | rs7021746 | 1.0000 | 1.0000 | 0.9790 | 1.0000 | 1.0000 | 1.0000 |
|  |  | rs10991925 | 0.9502 | 1.0000 | 0.8526 | 1.0000 | 0.9609 | 1.0000 |
|  |  | rs2440589 | 0.9502 | 1.0000 | 0.8444 | 1.0000 | 0.9609 | 1.0000 |
|  |  | rs2482357 | 1.0000 | 1.0000 | 0.9863 | 1.0000 | 0.9973 | 1.0000 |
|  |  | rs2482704 | 0.9502 | 1.0000 | 0.8444 | 1.0000 | 0.9609 | 1.0000 |
|  |  | rs2482359 | 0.9705 | 1.0000 | 0.8015 | 0.8974 | 0.9972 | 1.0000 |
|  |  | rs10820852 | 0.9502 | 1.0000 | 0.8444 | 1.0000 | 0.9880 | 1.0000 |
|  |  | rs13297268 | 0.9502 | 1.0000 | 0.8015 | 0.8519 | 0.9609 | 1.0000 |
|  |  | rs10116162 | 1.0000 | 1.0000 | 0.9771 | 1.0000 | 1.0000 | 1.0000 |
| *NPAS2* | 2 | rs13390078 | 1.0000 | 1.0000 | 0.9224 | 1.0000 | 1.0000 | 1.0000 |
|  |  | rs6542992 | 0.9705 | 1.0000 | 0.9612 | 1.0000 | 0.9976 | 1.0000 |
|  |  | rs75656425 | 0.9050 | 0.9816 | 0.9693 | 1.0000 | 0.9030 | 0.9861 |
|  |  | rs77568479 | 0.9502 | 1.0000 | 0.9863 | 1.0000 | 0.9609 | 1.0000 |
|  |  | rs78801659 | 0.9502 | 1.0000 | 0.9874 | 1.0000 | 0.9540 | 1.0000 |
|  |  | rs3896080 | 0.9502 | 1.0000 | 0.9771 | 1.0000 | 0.9374 | 0.9861 |
|  |  | rs79218385 | 1.0000 | 1.0000 | 0.8444 | 1.0000 | 1.0000 | 1.0000 |
|  |  | rs3860455 | 0.8604 | 0.9013 | 0.8375 | 1.0000 | 0.8722 | 0.9861 |
|  |  | rs57365275 | 0.9502 | 1.0000 | 0.9084 | 1.0000 | 0.9609 | 1.0000 |
|  |  | rs72627416 | 0.9502 | 1.0000 | 0.8587 | 1.0000 | 0.9609 | 1.0000 |
|  |  | rs6542994 | 1.0000 | 1.0000 | 0.9854 | 1.0000 | 1.0000 | 1.0000 |
|  |  | rs17699370 | 1.0000 | 1.0000 | 0.8444 | 1.0000 | 1.0000 | 1.0000 |
|  |  | rs55982284 | 1.0000 | 1.0000 | 0.9700 | 1.0000 | 1.0000 | 1.0000 |
|  |  | rs62156093 | 1.0000 | 1.0000 | 0.9771 | 1.0000 | 0.9976 | 1.0000 |
|  |  | rs13012930 | 0.9502 | 1.0000 | 0.9771 | 1.0000 | 0.9609 | 1.0000 |
|  |  | rs4611661 | 0.9502 | 1.0000 | 0.9793 | 1.0000 | 0.9609 | 1.0000 |
|  |  | rs4377354 | 0.9502 | 1.0000 | 0.8015 | 0.8469 | 0.9609 | 1.0000 |
|  |  | rs72816926 | 0.8604 | 0.9152 | 0.9986 | 1.0000 | 0.8881 | 0.9861 |
|  |  | rs11691732 | 0.9502 | 1.0000 | 0.9874 | 1.0000 | 0.9609 | 1.0000 |
|  |  | rs7582455 | 0.8604 | 0.9013 | 0.9700 | 1.0000 | 0.8722 | 0.9861 |
|  |  | rs2309992 | 1.0000 | 1.0000 | 0.9242 | 1.0000 | 1.0000 | 1.0000 |
|  |  | rs7598826 | 1.0000 | 1.0000 | 0.9693 | 1.0000 | 1.0000 | 1.0000 |
|  |  | rs59005495 | 0.9502 | 1.0000 | 0.9814 | 1.0000 | 0.9609 | 1.0000 |
|  |  | rs75803056 | 1.0000 | 1.0000 | 0.7844 | 0.8017 | 1.0000 | 1.0000 |
|  |  | rs76376883 | 1.0000 | 1.0000 | 0.9986 | 1.0000 | 1.0000 | 1.0000 |
|  |  | rs1811399 | 1.0000 | 1.0000 | 0.9771 | 1.0000 | 1.0000 | 1.0000 |
|  |  | rs983287 | 0.9502 | 1.0000 | 0.8015 | 0.9372 | 0.9609 | 1.0000 |
|  |  | rs2043534 | 0.9790 | 1.0000 | 0.9986 | 1.0000 | 0.9880 | 1.0000 |
|  |  | rs6759386 | 0.9502 | 1.0000 | 0.7844 | 0.8201 | 0.9609 | 1.0000 |
|  |  | rs930309 | 1.0000 | 1.0000 | 0.9771 | 1.0000 | 1.0000 | 1.0000 |
|  |  | rs12472321 | 0.9939 | 1.0000 | 0.9671 | 1.0000 | 1.0000 | 1.0000 |
|  |  | rs12476292 | 1.0000 | 1.0000 | 0.8176 | 0.9617 | 0.9939 | 1.0000 |
|  |  | rs17024926 | 0.8604 | 0.9618 | 0.9700 | 1.0000 | 0.8722 | 0.9861 |
|  |  | rs72627426 | 0.9502 | 1.0000 | 0.9890 | 1.0000 | 0.9540 | 1.0000 |
|  |  | rs72627427 | 0.9502 | 1.0000 | 0.9986 | 1.0000 | 0.9374 | 0.9895 |
|  |  | rs12712084 | 0.9502 | 1.0000 | 0.7844 | 0.7510 | 1.0000 | 1.0000 |
|  |  | rs1369481 | 0.9502 | 1.0000 | 0.7844 | 0.7830 | 0.9880 | 1.0000 |
|  |  | rs17654772 | 0.9812 | 1.0000 | 0.9771 | 1.0000 | 0.9880 | 1.0000 |
|  |  | rs920086 | 0.9502 | 1.0000 | 0.7844 | 0.8221 | 1.0000 | 1.0000 |
|  |  | rs11123853 | 1.0000 | 1.0000 | 0.9700 | 1.0000 | 1.0000 | 1.0000 |
|  |  | rs34333438 | 1.0000 | 1.0000 | 0.9671 | 1.0000 | 1.0000 | 1.0000 |
|  |  | rs72627430 | 1.0000 | 1.0000 | 0.7551 | 0.7360 | 1.0000 | 1.0000 |
|  |  | rs7570190 | 1.0000 | 1.0000 | 0.9538 | 1.0000 | 1.0000 | 1.0000 |
|  |  | rs12989454 | 1.0000 | 1.0000 | 0.9671 | 1.0000 | 1.0000 | 1.0000 |
|  |  | rs13418893 | 0.9502 | 1.0000 | 0.8444 | 1.0000 | 1.0000 | 1.0000 |
|  |  | rs11894322 | 1.0000 | 1.0000 | 0.9874 | 1.0000 | 1.0000 | 1.0000 |
|  |  | rs11894370 | 1.0000 | 1.0000 | 0.9986 | 1.0000 | 1.0000 | 1.0000 |
|  |  | rs356643 | 0.9705 | 1.0000 | 0.9863 | 1.0000 | 0.9609 | 1.0000 |
|  |  | rs77985008 | 0.8604 | 0.9013 | 0.9504 | 1.0000 | 0.8722 | 0.9861 |
|  |  | rs7602455 | 0.9502 | 1.0000 | 0.9874 | 1.0000 | 0.9374 | 0.9872 |
|  |  | rs17655330 | 0.9705 | 1.0000 | 0.8444 | 1.0000 | 0.9609 | 1.0000 |
|  |  | rs6725296 | 0.8604 | 0.9013 | 0.9986 | 1.0000 | 0.8722 | 0.9861 |
|  |  | rs79320559 | 0.8604 | 0.9013 | 0.9493 | 1.0000 | 0.8722 | 0.9861 |
|  |  | rs356652 | 0.9705 | 1.0000 | 0.9874 | 1.0000 | 0.9609 | 1.0000 |
|  |  | rs3754674 | 1.0000 | 1.0000 | 0.9084 | 1.0000 | 1.0000 | 1.0000 |
|  |  | rs3754675 | 0.8604 | 0.9415 | 0.8361 | 1.0000 | 0.9540 | 1.0000 |
|  |  | rs3820786 | 0.9705 | 1.0000 | 0.9224 | 1.0000 | 0.9742 | 1.0000 |
|  |  | rs13025524 | 1.0000 | 1.0000 | 0.9491 | 1.0000 | 1.0000 | 1.0000 |
|  |  | rs3768984 | 0.9502 | 1.0000 | 0.8587 | 1.0000 | 0.9880 | 1.0000 |
|  |  | rs17025005 | 0.9502 | 1.0000 | 0.8444 | 1.0000 | 0.9609 | 1.0000 |
|  |  | rs7605434 | 0.9502 | 1.0000 | 0.9594 | 1.0000 | 0.9546 | 1.0000 |
|  |  | rs13429998 | 0.8604 | 0.9013 | 0.9874 | 1.0000 | 0.8722 | 0.9861 |
|  |  | rs4851384 | 1.0000 | 1.0000 | 0.9693 | 1.0000 | 1.0000 | 1.0000 |
|  |  | rs3820787 | 0.9705 | 1.0000 | 0.9771 | 1.0000 | 0.9880 | 1.0000 |
|  |  | rs4851386 | 0.8604 | 0.9013 | 0.7844 | 0.7738 | 0.9540 | 1.0000 |
|  |  | rs73945847 | 0.9169 | 0.9926 | 0.7844 | 0.7831 | 0.9880 | 1.0000 |
|  |  | rs4851390 | 0.9502 | 1.0000 | 0.8015 | 0.9019 | 0.9972 | 1.0000 |
|  |  | rs882272 | 0.8604 | 0.9549 | 0.9771 | 1.0000 | 0.9030 | 0.9861 |
|  |  | rs895520 | 0.8604 | 0.9540 | 0.9771 | 1.0000 | 0.9030 | 0.9861 |
|  |  | rs6738097 | 0.8604 | 0.9105 | 0.7844 | 0.7412 | 0.9540 | 1.0000 |
|  |  | rs12622050 | 0.8604 | 0.9013 | 0.9224 | 1.0000 | 0.8722 | 0.9861 |
|  |  | rs17025078 | 0.9955 | 1.0000 | 0.9081 | 1.0000 | 1.0000 | 1.0000 |
|  |  | rs4851391 | 0.8604 | 0.9385 | 0.9986 | 1.0000 | 0.8722 | 0.9861 |
|  |  | rs3768985 | 0.9502 | 1.0000 | 0.9538 | 1.0000 | 0.9679 | 1.0000 |
|  |  | rs4851392 | 1.0000 | 1.0000 | 0.9771 | 1.0000 | 1.0000 | 1.0000 |
|  |  | rs2289950 | 0.9502 | 1.0000 | 0.9527 | 1.0000 | 0.9609 | 1.0000 |
|  |  | rs4851393 | 0.8604 | 0.9013 | 0.7856 | 0.8300 | 0.9540 | 1.0000 |
|  |  | rs1562313 | 0.9502 | 1.0000 | 0.7844 | 0.8114 | 0.9609 | 1.0000 |
|  |  | rs2305160 | 1.0000 | 1.0000 | 0.9793 | 1.0000 | 1.0000 | 1.0000 |
|  |  | rs2305159 | 0.9705 | 1.0000 | 0.9874 | 1.0000 | 0.9609 | 1.0000 |
|  |  | rs1542179 | 0.9502 | 1.0000 | 0.9874 | 1.0000 | 0.9609 | 1.0000 |
|  |  | rs1542178 | 1.0000 | 1.0000 | 0.9986 | 1.0000 | 1.0000 | 1.0000 |
|  |  | rs3768988 | 1.0000 | 1.0000 | 0.9771 | 1.0000 | 0.9972 | 1.0000 |
|  |  | rs62152925 | 0.8604 | 0.9329 | 0.9538 | 1.0000 | 0.9026 | 0.9861 |
|  |  | rs75763901 | 0.9960 | 1.0000 | 0.9456 | 1.0000 | 0.9973 | 1.0000 |
|  |  | rs75159075 | 0.9502 | 1.0000 | 0.8015 | 0.9374 | 0.9609 | 1.0000 |
|  |  | rs2278728 | 1.0000 | 1.0000 | 0.9634 | 1.0000 | 1.0000 | 1.0000 |
|  |  | rs2278727 | 0.8604 | 0.9013 | 0.9793 | 1.0000 | 0.8722 | 0.9861 |
|  |  | rs6719533 | 0.9502 | 1.0000 | 0.7844 | 0.7825 | 1.0000 | 1.0000 |
|  |  | rs3754677 | 1.0000 | 1.0000 | 0.9986 | 1.0000 | 1.0000 | 1.0000 |
|  |  | rs3754678 | 1.0000 | 1.0000 | 0.9980 | 1.0000 | 1.0000 | 1.0000 |
|  |  | rs3754680 | 1.0000 | 1.0000 | 0.9986 | 1.0000 | 1.0000 | 1.0000 |
|  |  | rs3768990 | 1.0000 | 1.0000 | 0.9771 | 1.0000 | 1.0000 | 1.0000 |
|  |  | rs9223 | 0.9705 | 1.0000 | 0.8587 | 1.0000 | 0.9973 | 1.0000 |
|  |  | rs3739008 | 0.9502 | 1.0000 | 0.8444 | 1.0000 | 0.9880 | 1.0000 |
| *NR1D1* | 17 | rs883871 | 0.8604 | 0.9013 | 0.9538 | 1.0000 | 0.8722 | 0.9861 |
|  |  | rs2071427 | 0.9502 | 1.0000 | 0.9793 | 1.0000 | 0.9546 | 1.0000 |
|  |  | rs2269457 | 1.0000 | 1.0000 | 0.9986 | 1.0000 | 1.0000 | 1.0000 |
|  |  | rs12941497 | 1.0000 | 1.0000 | 0.9874 | 1.0000 | 1.0000 | 1.0000 |
|  |  | rs939347 | 1.0000 | 1.0000 | 0.9863 | 1.0000 | 1.0000 | 1.0000 |
|  |  | rs2071570 | 1.0000 | 1.0000 | 0.9874 | 1.0000 | 1.0000 | 1.0000 |
| *NR1D2* | 3 | rs6778577 | 1.0000 | 1.0000 | 0.9771 | 1.0000 | 1.0000 | 1.0000 |
|  |  | rs34593533 | 1.0000 | 1.0000 | 0.9771 | 1.0000 | 1.0000 | 1.0000 |
|  |  | rs11922609 | 1.0000 | 1.0000 | 0.7844 | 0.8034 | 1.0000 | 1.0000 |
|  |  | rs4858095 | 1.0000 | 1.0000 | 0.8587 | 1.0000 | 0.9880 | 1.0000 |
|  |  | rs61556088 | 0.9790 | 1.0000 | 0.9986 | 1.0000 | 0.9880 | 1.0000 |
|  |  | rs13095392 | 1.0000 | 1.0000 | 0.8444 | 1.0000 | 1.0000 | 1.0000 |
|  |  | rs35962983 | 1.0000 | 1.0000 | 0.8587 | 1.0000 | 0.9880 | 1.0000 |
|  |  | rs11717862 | 1.0000 | 1.0000 | 0.7844 | 0.7915 | 0.9880 | 1.0000 |
|  |  | rs9882735 | 0.9743 | 1.0000 | 0.9771 | 1.0000 | 0.9540 | 1.0000 |
|  |  | rs76873718 | 0.9502 | 1.0000 | 0.9986 | 1.0000 | 0.9609 | 1.0000 |
| *PER1* | 17 | rs2304911 | 1.0000 | 1.0000 | 0.7844 | 0.7777 | 1.0000 | 1.0000 |
| *PER2* | 2 | rs934945 | 1.0000 | 1.0000 | 0.9793 | 1.0000 | 1.0000 | 1.0000 |
|  |  | rs6431590 | 1.0000 | 1.0000 | 0.7844 | 0.8075 | 1.0000 | 1.0000 |
|  |  | rs2304669 | 1.0000 | 1.0000 | 0.9693 | 1.0000 | 1.0000 | 1.0000 |
|  |  | rs3739064 | 1.0000 | 1.0000 | 0.9863 | 1.0000 | 1.0000 | 1.0000 |
|  |  | rs1972874 | 1.0000 | 1.0000 | 0.9634 | 1.0000 | 0.9972 | 1.0000 |
|  |  | rs2304677 | 1.0000 | 1.0000 | 0.9874 | 1.0000 | 1.0000 | 1.0000 |
|  |  | rs2304674 | 1.0000 | 1.0000 | 0.8015 | 0.8871 | 1.0000 | 1.0000 |
|  |  | rs71426512 | 0.9502 | 1.0000 | 0.9806 | 1.0000 | 0.9609 | 1.0000 |
|  |  | rs67860414 | 1.0000 | 1.0000 | 0.7844 | 0.7789 | 1.0000 | 1.0000 |
| *PER3* | 1 | rs228727 | 0.8604 | 0.9013 | 0.9874 | 1.0000 | 0.8722 | 0.9861 |
|  |  | rs11121023 | 0.9502 | 1.0000 | 0.9806 | 1.0000 | 0.9609 | 1.0000 |
|  |  | rs75307902 | 0.9705 | 1.0000 | 0.9693 | 1.0000 | 0.9609 | 1.0000 |
|  |  | rs10864315 | 0.9502 | 1.0000 | 0.9771 | 1.0000 | 0.9609 | 1.0000 |
|  |  | rs117562183 | 1.0000 | 1.0000 | 0.9168 | 1.0000 | 0.9973 | 1.0000 |
|  |  | rs228682 | 0.9502 | 1.0000 | 0.8015 | 0.8850 | 0.9609 | 1.0000 |
|  |  | rs77567305 | 0.9502 | 1.0000 | 0.7844 | 0.7504 | 0.9609 | 1.0000 |
|  |  | rs10746473 | 0.8604 | 0.9013 | 0.2937 | 0.4530 | 0.9609 | 1.0000 |
|  |  | rs2797685 | 0.8604 | 0.9013 | 0.5396 | 0.6971 | 0.9973 | 1.0000 |
|  |  | rs118049345 | 1.0000 | 1.0000 | 0.7856 | 0.8266 | 1.0000 | 1.0000 |
|  |  | rs1773135 | 1.0000 | 1.0000 | 0.9594 | 1.0000 | 1.0000 | 1.0000 |
|  |  | rs1689904 | 0.8604 | 0.9013 | 0.5971 | 0.7196 | 0.9972 | 1.0000 |
|  |  | rs1773138 | 0.8604 | 0.9013 | 0.9693 | 1.0000 | 0.8722 | 0.9861 |
|  |  | rs12563789 | 1.0000 | 1.0000 | 0.7844 | 0.7360 | 1.0000 | 1.0000 |
| *REV1* | 2 | rs2305354 | 1.0000 | 1.0000 | 0.8677 | 1.0000 | 0.9609 | 1.0000 |
|  |  | rs28382964 | 1.0000 | 1.0000 | 0.9491 | 1.0000 | 1.0000 | 1.0000 |
|  |  | rs2290261 | 1.0000 | 1.0000 | 0.9671 | 1.0000 | 0.9972 | 1.0000 |
|  |  | rs4535093 | 1.0000 | 1.0000 | 0.8015 | 0.9499 | 0.9880 | 1.0000 |
|  |  | rs12619546 | 0.9936 | 1.0000 | 0.9874 | 1.0000 | 0.9972 | 1.0000 |
|  |  | rs4341989 | 0.9502 | 1.0000 | 0.8444 | 1.0000 | 0.9609 | 1.0000 |
|  |  | rs3792146 | 1.0000 | 1.0000 | 0.8444 | 1.0000 | 1.0000 | 1.0000 |
|  |  | rs7585019 | 0.9705 | 1.0000 | 0.9863 | 1.0000 | 0.9609 | 1.0000 |
|  |  | rs28745277 | 0.8604 | 0.9013 | 0.9787 | 1.0000 | 0.8722 | 0.9861 |
| *RORA* | 15 | rs75461420 | 0.9743 | 1.0000 | 0.9403 | 1.0000 | 0.9880 | 1.0000 |
|  |  | rs3743266 | 1.0000 | 1.0000 | 0.9874 | 1.0000 | 1.0000 | 1.0000 |
|  |  | rs17270188 | 0.9502 | 1.0000 | 0.9787 | 1.0000 | 0.9546 | 1.0000 |
|  |  | rs78280589 | 0.9502 | 1.0000 | 0.7844 | 0.8051 | 0.9609 | 1.0000 |
|  |  | rs6494204 | 0.9502 | 1.0000 | 0.9594 | 1.0000 | 0.9609 | 1.0000 |
|  |  | rs10438338 | 0.9812 | 1.0000 | 0.8015 | 0.9156 | 1.0000 | 1.0000 |
|  |  | rs1866007 | 0.9502 | 1.0000 | 0.9084 | 1.0000 | 0.9546 | 1.0000 |
|  |  | rs10519051 | 0.9705 | 1.0000 | 0.9612 | 1.0000 | 0.9880 | 1.0000 |
|  |  | rs17237283 | 0.8604 | 0.9013 | 0.9594 | 1.0000 | 0.8881 | 0.9861 |
|  |  | rs4594196 | 0.9743 | 1.0000 | 0.9787 | 1.0000 | 0.9880 | 1.0000 |
|  |  | rs4774367 | 0.9502 | 1.0000 | 0.8976 | 1.0000 | 0.9609 | 1.0000 |
|  |  | rs11635975 | 1.0000 | 1.0000 | 0.8587 | 1.0000 | 1.0000 | 1.0000 |
|  |  | rs2028122 | 1.0000 | 1.0000 | 0.7844 | 0.8007 | 1.0000 | 1.0000 |
|  |  | rs8033552 | 1.0000 | 1.0000 | 0.8015 | 0.9314 | 1.0000 | 1.0000 |
|  |  | rs4775281 | 0.9502 | 1.0000 | 0.8015 | 0.9372 | 0.9609 | 1.0000 |
|  |  | rs116861339 | 1.0000 | 1.0000 | 0.9793 | 1.0000 | 1.0000 | 1.0000 |
|  |  | rs79360097 | 1.0000 | 1.0000 | 0.7844 | 0.8233 | 1.0000 | 1.0000 |
|  |  | rs8041381 | 0.9705 | 1.0000 | 0.8587 | 1.0000 | 0.9609 | 1.0000 |
|  |  | rs16942772 | 0.9502 | 1.0000 | 0.9634 | 1.0000 | 0.9546 | 1.0000 |
|  |  | rs340002 | 1.0000 | 1.0000 | 0.9806 | 1.0000 | 1.0000 | 1.0000 |
|  |  | rs11632600 | 1.0000 | 1.0000 | 0.9527 | 1.0000 | 1.0000 | 1.0000 |
|  |  | rs11634234 | 1.0000 | 1.0000 | 0.9700 | 1.0000 | 0.9976 | 1.0000 |
|  |  | rs340005 | 1.0000 | 1.0000 | 0.9771 | 1.0000 | 1.0000 | 1.0000 |
|  |  | rs2289162 | 0.9502 | 1.0000 | 0.9986 | 1.0000 | 0.9609 | 1.0000 |
|  |  | rs2289163 | 0.9502 | 1.0000 | 0.9874 | 1.0000 | 0.9609 | 1.0000 |
|  |  | rs339969 | 1.0000 | 1.0000 | 0.9700 | 1.0000 | 1.0000 | 1.0000 |
|  |  | rs12443044 | 1.0000 | 1.0000 | 0.9771 | 1.0000 | 0.9880 | 1.0000 |
|  |  | rs72748739 | 0.9502 | 1.0000 | 0.9986 | 1.0000 | 0.9609 | 1.0000 |
|  |  | rs340009 | 0.9705 | 1.0000 | 0.9806 | 1.0000 | 0.9546 | 1.0000 |
|  |  | rs12591786 | 0.9743 | 1.0000 | 0.9986 | 1.0000 | 0.9880 | 1.0000 |
|  |  | rs58306294 | 0.9705 | 1.0000 | 0.9787 | 1.0000 | 0.9742 | 1.0000 |
|  |  | rs340021 | 0.9502 | 1.0000 | 0.9874 | 1.0000 | 0.9609 | 1.0000 |
|  |  | rs340023 | 0.9502 | 1.0000 | 0.8015 | 0.9441 | 1.0000 | 1.0000 |
|  |  | rs340026 | 0.8604 | 0.9684 | 0.9380 | 1.0000 | 0.9374 | 0.9861 |
|  |  | rs3784611 | 0.9050 | 0.9882 | 0.9538 | 1.0000 | 0.9540 | 0.9984 |
|  |  | rs3784610 | 1.0000 | 1.0000 | 0.9874 | 1.0000 | 0.9880 | 1.0000 |
|  |  | rs3784609 | 1.0000 | 1.0000 | 0.7844 | 0.7560 | 1.0000 | 1.0000 |
|  |  | rs72748759 | 1.0000 | 1.0000 | 0.7844 | 0.7869 | 1.0000 | 1.0000 |
|  |  | rs28408562 | 1.0000 | 1.0000 | 0.8015 | 0.8941 | 0.8881 | 0.9861 |
|  |  | rs28724570 | 1.0000 | 1.0000 | 0.8015 | 0.8935 | 0.9540 | 1.0000 |
|  |  | rs75981965 | 0.9502 | 1.0000 | 0.9986 | 1.0000 | 0.9609 | 1.0000 |
|  |  | rs72748764 | 1.0000 | 1.0000 | 0.8390 | 1.0000 | 1.0000 | 1.0000 |
|  |  | rs1657792 | 1.0000 | 1.0000 | 0.9793 | 1.0000 | 0.9959 | 1.0000 |
|  |  | rs11630262 | 1.0000 | 1.0000 | 0.9456 | 1.0000 | 1.0000 | 1.0000 |
|  |  | rs78746013 | 0.9502 | 1.0000 | 0.9890 | 1.0000 | 0.9609 | 1.0000 |
|  |  | rs75995412 | 0.9502 | 1.0000 | 0.9874 | 1.0000 | 0.9609 | 1.0000 |
|  |  | rs2241794 | 1.0000 | 1.0000 | 0.9693 | 1.0000 | 1.0000 | 1.0000 |
|  |  | rs80313728 | 1.0000 | 1.0000 | 0.9806 | 1.0000 | 1.0000 | 1.0000 |
|  |  | rs339995 | 1.0000 | 1.0000 | 0.9793 | 1.0000 | 1.0000 | 1.0000 |
|  |  | rs339996 | 0.9705 | 1.0000 | 0.8015 | 0.9100 | 0.9880 | 1.0000 |
|  |  | rs17237318 | 1.0000 | 1.0000 | 0.8587 | 1.0000 | 1.0000 | 1.0000 |
|  |  | rs339998 | 0.9713 | 1.0000 | 0.9700 | 1.0000 | 0.9972 | 1.0000 |
|  |  | rs9630427 | 0.8604 | 0.9013 | 0.9242 | 1.0000 | 0.8881 | 0.9861 |
|  |  | rs16942900 | 1.0000 | 1.0000 | 0.9986 | 1.0000 | 1.0000 | 1.0000 |
|  |  | rs79416181 | 1.0000 | 1.0000 | 0.9771 | 1.0000 | 1.0000 | 1.0000 |
|  |  | rs2433026 | 1.0000 | 1.0000 | 0.9926 | 1.0000 | 0.9973 | 1.0000 |
|  |  | rs41356552 | 0.9502 | 1.0000 | 0.9594 | 1.0000 | 0.9609 | 1.0000 |
|  |  | rs7166370 | 0.9502 | 1.0000 | 0.9634 | 1.0000 | 0.9609 | 1.0000 |
|  |  | rs12437754 | 0.9502 | 1.0000 | 0.8015 | 0.9478 | 0.9609 | 1.0000 |
|  |  | rs1657800 | 0.9502 | 1.0000 | 0.9771 | 1.0000 | 0.9609 | 1.0000 |
|  |  | rs11629660 | 0.9502 | 1.0000 | 0.9700 | 1.0000 | 0.9609 | 1.0000 |
|  |  | rs11629864 | 1.0000 | 1.0000 | 0.9874 | 1.0000 | 1.0000 | 1.0000 |
|  |  | rs7172874 | 0.9502 | 1.0000 | 0.9793 | 1.0000 | 0.9609 | 1.0000 |
|  |  | rs74800820 | 1.0000 | 1.0000 | 0.9504 | 1.0000 | 1.0000 | 1.0000 |
|  |  | rs2553234 | 1.0000 | 1.0000 | 0.9986 | 1.0000 | 1.0000 | 1.0000 |
|  |  | rs9920661 | 1.0000 | 1.0000 | 0.9874 | 1.0000 | 1.0000 | 1.0000 |
|  |  | rs9920767 | 1.0000 | 1.0000 | 0.9854 | 1.0000 | 1.0000 | 1.0000 |
|  |  | rs76105700 | 1.0000 | 1.0000 | 0.9863 | 1.0000 | 1.0000 | 1.0000 |
|  |  | rs880626 | 0.9502 | 1.0000 | 0.9752 | 1.0000 | 0.9540 | 1.0000 |
|  |  | rs880625 | 0.9169 | 0.9937 | 0.9854 | 1.0000 | 0.8881 | 0.9861 |
|  |  | rs2553236 | 0.9999 | 1.0000 | 0.8015 | 0.8871 | 1.0000 | 1.0000 |
|  |  | rs8040332 | 1.0000 | 1.0000 | 0.7844 | 0.7948 | 1.0000 | 1.0000 |
|  |  | rs8042370 | 1.0000 | 1.0000 | 0.9771 | 1.0000 | 1.0000 | 1.0000 |
|  |  | rs919000 | 1.0000 | 1.0000 | 0.9833 | 1.0000 | 1.0000 | 1.0000 |
|  |  | rs999449 | 1.0000 | 1.0000 | 0.8361 | 1.0000 | 1.0000 | 1.0000 |
|  |  | rs11629812 | 0.9705 | 1.0000 | 0.8587 | 1.0000 | 0.9972 | 1.0000 |
|  |  | rs59558657 | 0.9705 | 1.0000 | 0.9986 | 1.0000 | 0.9880 | 1.0000 |
|  |  | rs6494217 | 1.0000 | 1.0000 | 0.9594 | 1.0000 | 0.9609 | 1.0000 |
|  |  | rs118138621 | 0.9705 | 1.0000 | 0.9986 | 1.0000 | 0.9880 | 1.0000 |
|  |  | rs1425287 | 0.9502 | 1.0000 | 0.9806 | 1.0000 | 0.9609 | 1.0000 |
|  |  | rs78164583 | 1.0000 | 1.0000 | 0.9671 | 1.0000 | 1.0000 | 1.0000 |
|  |  | rs117194204 | 0.9502 | 1.0000 | 0.9863 | 1.0000 | 0.9609 | 1.0000 |
|  |  | rs13329238 | 0.9502 | 1.0000 | 0.7844 | 0.7896 | 0.9609 | 1.0000 |
|  |  | rs4774371 | 1.0000 | 1.0000 | 0.9627 | 1.0000 | 1.0000 | 1.0000 |
|  |  | rs17237346 | 0.9502 | 1.0000 | 0.9787 | 1.0000 | 0.9609 | 1.0000 |
|  |  | rs8027032 | 0.9502 | 1.0000 | 0.9504 | 1.0000 | 0.9609 | 1.0000 |
|  |  | rs8038077 | 1.0000 | 1.0000 | 0.9793 | 1.0000 | 1.0000 | 1.0000 |
|  |  | rs2433025 | 0.9502 | 1.0000 | 0.8015 | 0.8779 | 0.9880 | 1.0000 |
|  |  | rs17303111 | 0.9502 | 1.0000 | 0.9235 | 1.0000 | 0.9609 | 1.0000 |
|  |  | rs7173461 | 0.9705 | 1.0000 | 0.9084 | 1.0000 | 0.9880 | 1.0000 |
|  |  | rs2414680 | 0.9502 | 1.0000 | 0.8587 | 1.0000 | 0.9609 | 1.0000 |
|  |  | rs6494219 | 0.9743 | 1.0000 | 0.9392 | 1.0000 | 0.9679 | 1.0000 |
|  |  | rs6494221 | 1.0000 | 1.0000 | 0.8353 | 0.9963 | 1.0000 | 1.0000 |
|  |  | rs12899193 | 0.9050 | 0.9866 | 0.9863 | 1.0000 | 0.8722 | 0.9861 |
|  |  | rs16943000 | 0.8604 | 0.9647 | 0.9986 | 1.0000 | 0.9030 | 0.9861 |
|  |  | rs11071551 | 1.0000 | 1.0000 | 0.9793 | 1.0000 | 1.0000 | 1.0000 |
|  |  | rs16943012 | 0.9502 | 1.0000 | 0.9986 | 1.0000 | 0.9609 | 1.0000 |
|  |  | rs1834335 | 1.0000 | 1.0000 | 0.9693 | 1.0000 | 1.0000 | 1.0000 |
|  |  | rs1820357 | 0.8604 | 0.9013 | 0.8015 | 0.9019 | 0.8881 | 0.9861 |
|  |  | rs17237353 | 0.9502 | 1.0000 | 0.9671 | 1.0000 | 0.9540 | 1.0000 |
|  |  | rs12591749 | 0.9502 | 1.0000 | 0.8444 | 1.0000 | 0.9609 | 1.0000 |
|  |  | rs341413 | 1.0000 | 1.0000 | 0.9224 | 1.0000 | 1.0000 | 1.0000 |
|  |  | rs35277300 | 0.8604 | 0.9389 | 0.7844 | 0.7452 | 0.9609 | 1.0000 |
|  |  | rs17237367 | 0.8604 | 0.9013 | 0.5286 | 0.6649 | 0.8881 | 0.9861 |
|  |  | rs78554936 | 0.8604 | 0.9013 | 0.8015 | 0.9332 | 0.8881 | 0.9861 |
|  |  | rs7168905 | 1.0000 | 1.0000 | 0.7455 | 0.7360 | 1.0000 | 1.0000 |
|  |  | rs9920962 | 1.0000 | 1.0000 | 0.9771 | 1.0000 | 1.0000 | 1.0000 |
|  |  | rs76194223 | 0.8604 | 0.9013 | 0.8353 | 1.0000 | 0.8722 | 0.9861 |
|  |  | rs4775292 | 1.0000 | 1.0000 | 0.8015 | 0.8425 | 1.0000 | 1.0000 |
|  |  | rs7172011 | 1.0000 | 1.0000 | 0.8863 | 1.0000 | 1.0000 | 1.0000 |
|  |  | rs1993471 | 1.0000 | 1.0000 | 0.9986 | 1.0000 | 1.0000 | 1.0000 |
|  |  | rs17204367 | 0.9050 | 0.9853 | 0.8015 | 0.9420 | 0.9609 | 1.0000 |
|  |  | rs17303153 | 0.8604 | 0.9013 | 0.8015 | 0.8428 | 0.8722 | 0.9861 |
|  |  | rs1020729 | 0.8604 | 0.9072 | 0.8015 | 0.8862 | 0.9546 | 1.0000 |
|  |  | rs58469372 | 0.8604 | 0.9013 | 0.7844 | 0.8233 | 0.8722 | 0.9861 |
|  |  | rs1020730 | 0.8604 | 0.9485 | 0.9671 | 1.0000 | 0.9026 | 0.9861 |
|  |  | rs17204402 | 1.0000 | 1.0000 | 0.9256 | 1.0000 | 1.0000 | 1.0000 |
|  |  | rs961299 | 0.8604 | 0.9013 | 0.9863 | 1.0000 | 0.8722 | 0.9861 |
|  |  | rs12900122 | 0.9502 | 1.0000 | 0.8260 | 0.9875 | 0.9609 | 1.0000 |
|  |  | rs2279297 | 1.0000 | 1.0000 | 0.7844 | 0.8162 | 1.0000 | 1.0000 |
|  |  | rs8025689 | 0.8604 | 0.9013 | 0.8353 | 1.0000 | 0.8881 | 0.9861 |
|  |  | rs17204426 | 1.0000 | 1.0000 | 0.8353 | 1.0000 | 1.0000 | 1.0000 |
|  |  | rs62002747 | 1.0000 | 1.0000 | 0.8256 | 0.9747 | 1.0000 | 1.0000 |
|  |  | rs9302215 | 0.8604 | 0.9395 | 0.7844 | 0.7360 | 0.9609 | 1.0000 |
|  |  | rs12591650 | 0.8604 | 0.9013 | 0.5396 | 0.7013 | 0.9546 | 1.0000 |
|  |  | rs1482057 | 1.0000 | 1.0000 | 0.9169 | 1.0000 | 1.0000 | 1.0000 |
|  |  | rs17204440 | 0.9628 | 1.0000 | 0.8015 | 0.8556 | 0.9880 | 1.0000 |
|  |  | rs11639084 | 0.9502 | 1.0000 | 0.8015 | 0.8807 | 0.9546 | 1.0000 |
|  |  | rs12594188 | 0.8604 | 0.9013 | 0.5286 | 0.6611 | 0.9374 | 0.9861 |
|  |  | rs10519067 | 0.9502 | 1.0000 | 0.9806 | 1.0000 | 0.9609 | 1.0000 |
|  |  | rs12438866 | 1.0000 | 1.0000 | 0.9771 | 1.0000 | 1.0000 | 1.0000 |
|  |  | rs10519070 | 0.8604 | 0.9119 | 0.8256 | 0.9744 | 0.9374 | 0.9861 |
|  |  | rs62002749 | 1.0000 | 1.0000 | 0.7844 | 0.7360 | 1.0000 | 1.0000 |
|  |  | rs11071557 | 1.0000 | 1.0000 | 0.9863 | 1.0000 | 1.0000 | 1.0000 |
|  |  | rs11071558 | 1.0000 | 1.0000 | 0.9863 | 1.0000 | 1.0000 | 1.0000 |
|  |  | rs11071559 | 1.0000 | 1.0000 | 0.9806 | 1.0000 | 1.0000 | 1.0000 |
|  |  | rs922782 | 1.0000 | 1.0000 | 0.8015 | 0.8574 | 1.0000 | 1.0000 |
|  |  | rs922781 | 1.0000 | 1.0000 | 0.8353 | 1.0000 | 1.0000 | 1.0000 |
|  |  | rs4774372 | 0.8604 | 0.9013 | 0.8048 | 0.9526 | 0.8881 | 0.9861 |
|  |  | rs1963497 | 1.0000 | 1.0000 | 0.7844 | 0.7499 | 1.0000 | 1.0000 |
|  |  | rs17270446 | 1.0000 | 1.0000 | 0.4625 | 0.5821 | 1.0000 | 1.0000 |
|  |  | rs2899662 | 1.0000 | 1.0000 | 0.7844 | 0.7360 | 1.0000 | 1.0000 |
|  |  | rs1680446 | 1.0000 | 1.0000 | 0.7844 | 0.7371 | 1.0000 | 1.0000 |
|  |  | rs877228 | 0.9502 | 1.0000 | 0.9634 | 1.0000 | 0.8722 | 0.9861 |
|  |  | rs4775297 | 0.9502 | 1.0000 | 0.7844 | 0.7485 | 0.9609 | 1.0000 |
|  |  | rs16943117 | 1.0000 | 1.0000 | 0.9538 | 1.0000 | 1.0000 | 1.0000 |
|  |  | rs12915776 | 1.0000 | 1.0000 | 0.9693 | 1.0000 | 1.0000 | 1.0000 |
|  |  | rs341459 | 0.9705 | 1.0000 | 0.9874 | 1.0000 | 0.9880 | 1.0000 |
|  |  | rs12593925 | 0.8604 | 0.9438 | 0.8256 | 0.9845 | 0.9540 | 1.0000 |
|  |  | rs78498480 | 0.9502 | 1.0000 | 0.8444 | 1.0000 | 0.9609 | 1.0000 |
|  |  | rs10162630 | 0.9502 | 1.0000 | 0.9874 | 1.0000 | 0.9374 | 0.9861 |
|  |  | rs12591848 | 0.9502 | 1.0000 | 0.9493 | 1.0000 | 0.9609 | 1.0000 |
|  |  | rs12440185 | 0.8604 | 0.9013 | 0.9793 | 1.0000 | 0.8722 | 0.9861 |
|  |  | rs12902540 | 0.9502 | 1.0000 | 0.9612 | 1.0000 | 0.9609 | 1.0000 |
|  |  | rs7162615 | 0.8604 | 0.9013 | 0.8354 | 1.0000 | 0.8722 | 0.9861 |
|  |  | rs17270459 | 1.0000 | 1.0000 | 0.7844 | 0.8186 | 1.0000 | 1.0000 |
|  |  | rs875339 | 1.0000 | 1.0000 | 0.7844 | 0.7493 | 1.0000 | 1.0000 |
|  |  | rs62004360 | 1.0000 | 1.0000 | 0.8015 | 0.8845 | 1.0000 | 1.0000 |
|  |  | rs341366 | 1.0000 | 1.0000 | 0.9854 | 1.0000 | 0.9972 | 1.0000 |
|  |  | rs16943131 | 0.8604 | 0.9013 | 0.9806 | 1.0000 | 0.8722 | 0.9861 |
|  |  | rs75866172 | 0.8604 | 0.9013 | 0.9874 | 1.0000 | 0.8722 | 0.9861 |
|  |  | rs6494225 | 0.8604 | 0.9013 | 0.9787 | 1.0000 | 0.8722 | 0.9861 |
|  |  | rs6494227 | 0.8604 | 0.9013 | 0.8353 | 1.0000 | 0.8722 | 0.9861 |
|  |  | rs79610262 | 0.8604 | 0.9013 | 0.8410 | 1.0000 | 0.8722 | 0.9861 |
|  |  | rs10519076 | 1.0000 | 1.0000 | 0.7844 | 0.7360 | 0.9880 | 1.0000 |
|  |  | rs341373 | 0.9589 | 1.0000 | 0.8256 | 0.9706 | 0.9880 | 1.0000 |
|  |  | rs78507043 | 1.0000 | 1.0000 | 0.9986 | 1.0000 | 1.0000 | 1.0000 |
|  |  | rs10152719 | 1.0000 | 1.0000 | 0.7844 | 0.7455 | 0.9880 | 1.0000 |
|  |  | rs341381 | 1.0000 | 1.0000 | 0.8015 | 0.9047 | 0.9972 | 1.0000 |
|  |  | rs16943172 | 1.0000 | 1.0000 | 0.9538 | 1.0000 | 1.0000 | 1.0000 |
|  |  | rs12439995 | 1.0000 | 1.0000 | 0.7844 | 0.7360 | 1.0000 | 1.0000 |
|  |  | rs341392 | 1.0000 | 1.0000 | 0.9594 | 1.0000 | 0.9609 | 1.0000 |
|  |  | rs6494229 | 0.9743 | 1.0000 | 0.9814 | 1.0000 | 0.9880 | 1.0000 |
|  |  | rs8041061 | 0.9502 | 1.0000 | 0.9671 | 1.0000 | 0.9609 | 1.0000 |
|  |  | rs8042149 | 0.9502 | 1.0000 | 0.9771 | 1.0000 | 0.9609 | 1.0000 |
|  |  | rs4775301 | 0.9812 | 1.0000 | 0.7844 | 0.8128 | 1.0000 | 1.0000 |
|  |  | rs6494230 | 0.9502 | 1.0000 | 0.7844 | 0.8110 | 1.0000 | 1.0000 |
|  |  | rs11634976 | 0.9705 | 1.0000 | 0.8015 | 0.8678 | 1.0000 | 1.0000 |
|  |  | rs8023252 | 1.0000 | 1.0000 | 0.9594 | 1.0000 | 0.9939 | 1.0000 |
|  |  | rs341398 | 1.0000 | 1.0000 | 0.9693 | 1.0000 | 1.0000 | 1.0000 |
|  |  | rs1224251 | 1.0000 | 1.0000 | 0.7844 | 0.7490 | 1.0000 | 1.0000 |
|  |  | rs11630062 | 0.9502 | 1.0000 | 0.6968 | 0.7333 | 1.0000 | 1.0000 |
|  |  | rs341403 | 1.0000 | 1.0000 | 0.9986 | 1.0000 | 1.0000 | 1.0000 |
|  |  | rs12595623 | 1.0000 | 1.0000 | 0.9961 | 1.0000 | 1.0000 | 1.0000 |
|  |  | rs11630227 | 0.9713 | 1.0000 | 0.9986 | 1.0000 | 0.9609 | 1.0000 |
|  |  | rs10519080 | 0.9502 | 1.0000 | 0.9594 | 1.0000 | 0.9609 | 1.0000 |
|  |  | rs341408 | 0.9812 | 1.0000 | 0.9527 | 1.0000 | 0.9609 | 1.0000 |
|  |  | rs17204545 | 1.0000 | 1.0000 | 0.9986 | 1.0000 | 1.0000 | 1.0000 |
|  |  | rs79409065 | 1.0000 | 1.0000 | 0.9874 | 1.0000 | 0.9972 | 1.0000 |
|  |  | rs341411 | 0.9502 | 1.0000 | 0.9671 | 1.0000 | 0.9609 | 1.0000 |
|  |  | rs75084363 | 0.9502 | 1.0000 | 0.9671 | 1.0000 | 0.9609 | 1.0000 |
|  |  | rs11858268 | 0.9502 | 1.0000 | 0.9986 | 1.0000 | 0.9609 | 1.0000 |
|  |  | rs7497885 | 0.9705 | 1.0000 | 0.9594 | 1.0000 | 0.9609 | 1.0000 |
|  |  | rs2306502 | 1.0000 | 1.0000 | 0.9863 | 1.0000 | 1.0000 | 1.0000 |
|  |  | rs10519085 | 0.9741 | 1.0000 | 0.8375 | 1.0000 | 1.0000 | 1.0000 |
|  |  | rs8041466 | 1.0000 | 1.0000 | 0.8587 | 1.0000 | 1.0000 | 1.0000 |
|  |  | rs12913890 | 1.0000 | 1.0000 | 0.9771 | 1.0000 | 1.0000 | 1.0000 |
|  |  | rs72750668 | 1.0000 | 1.0000 | 0.9793 | 1.0000 | 1.0000 | 1.0000 |
|  |  | rs77282013 | 1.0000 | 1.0000 | 0.9986 | 1.0000 | 1.0000 | 1.0000 |
|  |  | rs1902618 | 0.9812 | 1.0000 | 0.9380 | 1.0000 | 0.9972 | 1.0000 |
|  |  | rs341365 | 1.0000 | 1.0000 | 0.9787 | 1.0000 | 0.9609 | 1.0000 |
|  |  | rs7182392 | 1.0000 | 1.0000 | 0.9594 | 1.0000 | 1.0000 | 1.0000 |
|  |  | rs4775309 | 1.0000 | 1.0000 | 0.9095 | 1.0000 | 1.0000 | 1.0000 |
|  |  | rs341387 | 1.0000 | 1.0000 | 0.8015 | 0.8633 | 0.9546 | 1.0000 |
|  |  | rs11631432 | 1.0000 | 1.0000 | 0.9874 | 1.0000 | 0.9609 | 1.0000 |
|  |  | rs4775311 | 1.0000 | 1.0000 | 0.9224 | 1.0000 | 1.0000 | 1.0000 |
|  |  | rs8039990 | 1.0000 | 1.0000 | 0.9833 | 1.0000 | 0.9880 | 1.0000 |
|  |  | rs8040450 | 1.0000 | 1.0000 | 0.9538 | 1.0000 | 0.9880 | 1.0000 |
|  |  | rs341389 | 0.9790 | 1.0000 | 0.9863 | 1.0000 | 0.9609 | 1.0000 |
|  |  | rs12907550 | 1.0000 | 1.0000 | 0.9793 | 1.0000 | 1.0000 | 1.0000 |
|  |  | rs8036723 | 1.0000 | 1.0000 | 0.9986 | 1.0000 | 0.9976 | 1.0000 |
|  |  | rs16943284 | 1.0000 | 1.0000 | 0.9793 | 1.0000 | 1.0000 | 1.0000 |
|  |  | rs12915127 | 1.0000 | 1.0000 | 0.9538 | 1.0000 | 1.0000 | 1.0000 |
|  |  | rs28692829 | 0.9502 | 1.0000 | 0.9671 | 1.0000 | 0.9540 | 1.0000 |
|  |  | rs2414682 | 1.0000 | 1.0000 | 0.8677 | 1.0000 | 1.0000 | 1.0000 |
|  |  | rs79067694 | 1.0000 | 1.0000 | 0.7844 | 0.8008 | 1.0000 | 1.0000 |
|  |  | rs729977 | 1.0000 | 1.0000 | 0.9594 | 1.0000 | 0.9880 | 1.0000 |
|  |  | rs7172917 | 1.0000 | 1.0000 | 0.9986 | 1.0000 | 1.0000 | 1.0000 |
|  |  | rs4775313 | 1.0000 | 1.0000 | 0.9693 | 1.0000 | 1.0000 | 1.0000 |
|  |  | rs4774376 | 1.0000 | 1.0000 | 0.8587 | 1.0000 | 1.0000 | 1.0000 |
|  |  | rs35598844 | 0.9502 | 1.0000 | 0.9771 | 1.0000 | 0.9609 | 1.0000 |
|  |  | rs7168782 | 0.9502 | 1.0000 | 0.9986 | 1.0000 | 0.9609 | 1.0000 |
|  |  | rs117779544 | 1.0000 | 1.0000 | 0.8274 | 0.9894 | 1.0000 | 1.0000 |
|  |  | rs8041087 | 1.0000 | 1.0000 | 0.9634 | 1.0000 | 1.0000 | 1.0000 |
|  |  | rs2414686 | 1.0000 | 1.0000 | 0.9479 | 1.0000 | 0.9660 | 1.0000 |
|  |  | rs877862 | 1.0000 | 1.0000 | 0.9671 | 1.0000 | 1.0000 | 1.0000 |
|  |  | rs12904857 | 0.9705 | 1.0000 | 0.9863 | 1.0000 | 0.9880 | 1.0000 |
|  |  | rs12910281 | 1.0000 | 1.0000 | 0.7844 | 0.7360 | 0.9609 | 1.0000 |
|  |  | rs12909379 | 1.0000 | 1.0000 | 0.8015 | 0.9049 | 0.9609 | 1.0000 |
|  |  | rs16943299 | 1.0000 | 1.0000 | 0.9594 | 1.0000 | 1.0000 | 1.0000 |
|  |  | rs117795767 | 1.0000 | 1.0000 | 0.8015 | 0.9086 | 1.0000 | 1.0000 |
|  |  | rs2899664 | 1.0000 | 1.0000 | 0.9671 | 1.0000 | 1.0000 | 1.0000 |
|  |  | rs1054789 | 1.0000 | 1.0000 | 0.7844 | 0.7991 | 0.9679 | 1.0000 |
|  |  | rs2062091 | 1.0000 | 1.0000 | 0.7844 | 0.7628 | 0.9609 | 1.0000 |
|  |  | rs1384121 | 0.9502 | 1.0000 | 0.7844 | 0.7983 | 0.9609 | 1.0000 |
|  |  | rs11855147 | 0.9940 | 1.0000 | 0.9793 | 1.0000 | 0.9972 | 1.0000 |
|  |  | rs8027424 | 0.8604 | 0.9686 | 0.7844 | 0.7540 | 0.9609 | 1.0000 |
|  |  | rs17237486 | 0.9502 | 1.0000 | 0.7844 | 0.7557 | 1.0000 | 1.0000 |
|  |  | rs7162937 | 0.9502 | 1.0000 | 0.9854 | 1.0000 | 0.9609 | 1.0000 |
|  |  | rs12148149 | 0.9502 | 1.0000 | 0.9874 | 1.0000 | 0.9609 | 1.0000 |
|  |  | rs12901574 | 0.9251 | 0.9961 | 0.7844 | 0.7837 | 0.9609 | 1.0000 |
|  |  | rs5813053 | 0.8604 | 0.9013 | 0.7856 | 0.8323 | 0.9030 | 0.9861 |
|  |  | rs6494232 | 0.9502 | 1.0000 | 0.7844 | 0.8227 | 1.0000 | 1.0000 |
|  |  | rs16943318 | 0.9502 | 1.0000 | 0.9949 | 1.0000 | 0.9609 | 1.0000 |
|  |  | rs8028796 | 1.0000 | 1.0000 | 0.9594 | 1.0000 | 1.0000 | 1.0000 |
|  |  | rs4775318 | 0.9502 | 1.0000 | 0.9874 | 1.0000 | 0.9609 | 1.0000 |
|  |  | rs2062094 | 0.9502 | 1.0000 | 0.9854 | 1.0000 | 0.9546 | 1.0000 |
|  |  | rs2062092 | 0.9936 | 1.0000 | 0.9874 | 1.0000 | 0.9959 | 1.0000 |
|  |  | rs1482052 | 0.9705 | 1.0000 | 0.9771 | 1.0000 | 0.9880 | 1.0000 |
|  |  | rs10220727 | 0.9502 | 1.0000 | 0.9818 | 1.0000 | 0.9609 | 1.0000 |
|  |  | rs17303258 | 0.9502 | 1.0000 | 0.9914 | 1.0000 | 0.9609 | 1.0000 |
|  |  | rs35715615 | 0.9705 | 1.0000 | 0.9671 | 1.0000 | 0.9546 | 1.0000 |
|  |  | rs2279291 | 0.9502 | 1.0000 | 0.9874 | 1.0000 | 0.9540 | 0.9975 |
|  |  | rs1482049 | 0.9502 | 1.0000 | 0.9793 | 1.0000 | 0.9355 | 0.9861 |
|  |  | rs79995443 | 0.9502 | 1.0000 | 0.8015 | 0.9472 | 0.9609 | 1.0000 |
|  |  | rs28705880 | 0.8604 | 0.9013 | 0.7844 | 0.7930 | 0.8722 | 0.9861 |
|  |  | rs1351545 | 0.9705 | 1.0000 | 0.9863 | 1.0000 | 0.9609 | 1.0000 |
|  |  | rs8034886 | 0.9502 | 1.0000 | 0.9594 | 1.0000 | 0.9540 | 1.0000 |
|  |  | rs4775328 | 0.9050 | 0.9870 | 0.8784 | 1.0000 | 0.9540 | 1.0000 |
|  |  | rs79271390 | 1.0000 | 1.0000 | 0.9980 | 1.0000 | 1.0000 | 1.0000 |
|  |  | rs58413143 | 0.8604 | 0.9603 | 0.9863 | 1.0000 | 0.8722 | 0.9861 |
|  |  | rs7176774 | 1.0000 | 1.0000 | 0.9793 | 1.0000 | 1.0000 | 1.0000 |
|  |  | rs72752780 | 0.8604 | 0.9013 | 0.9484 | 1.0000 | 0.8722 | 0.9861 |
|  |  | rs4335725 | 0.9502 | 1.0000 | 0.8444 | 1.0000 | 0.9540 | 1.0000 |
|  |  | rs12903220 | 0.9705 | 1.0000 | 0.8015 | 0.9013 | 0.9609 | 1.0000 |
|  |  | rs12593927 | 0.9502 | 1.0000 | 0.9081 | 1.0000 | 0.9609 | 1.0000 |
|  |  | rs8029848 | 0.8604 | 0.9013 | 0.1762 | 0.3022 | 0.8722 | 0.9861 |
|  |  | rs8034880 | 0.8604 | 0.9013 | 0.1762 | 0.2206 | 0.8722 | 0.9861 |
|  |  | rs8034950 | 0.8604 | 0.9013 | 0.7844 | 0.7447 | 0.9030 | 0.9861 |
|  |  | rs28575275 | 0.9502 | 1.0000 | 0.9874 | 1.0000 | 0.9030 | 0.9861 |
|  |  | rs12912233 | 0.9502 | 1.0000 | 0.7844 | 0.7570 | 0.9972 | 1.0000 |
|  |  | rs4775339 | 1.0000 | 1.0000 | 0.9874 | 1.0000 | 1.0000 | 1.0000 |
|  |  | rs4775340 | 1.0000 | 1.0000 | 0.7844 | 0.7360 | 1.0000 | 1.0000 |
|  |  | rs17237521 | 0.8604 | 0.9013 | 0.8375 | 1.0000 | 0.8722 | 0.9861 |
|  |  | rs6494237 | 0.8604 | 0.9013 | 0.9084 | 1.0000 | 0.8722 | 0.9861 |
|  |  | rs72752802 | 0.8604 | 0.9013 | 0.8015 | 0.9139 | 0.8722 | 0.9861 |
|  |  | rs2140442 | 0.9930 | 1.0000 | 0.9594 | 1.0000 | 0.9972 | 1.0000 |
|  |  | rs7168987 | 0.9705 | 1.0000 | 0.8441 | 1.0000 | 0.9742 | 1.0000 |
|  |  | rs11631656 | 0.8604 | 0.9013 | 0.9527 | 1.0000 | 0.8722 | 0.9861 |
|  |  | rs7176329 | 1.0000 | 1.0000 | 0.8015 | 0.9138 | 1.0000 | 1.0000 |
|  |  | rs16943444 | 0.9502 | 1.0000 | 0.8444 | 1.0000 | 1.0000 | 1.0000 |
|  |  | rs1467304 | 0.8604 | 0.9013 | 0.9224 | 1.0000 | 0.8722 | 0.9861 |
|  |  | rs7174217 | 0.9502 | 1.0000 | 0.8256 | 0.9803 | 0.9880 | 1.0000 |
|  |  | rs7171713 | 1.0000 | 1.0000 | 0.8587 | 1.0000 | 1.0000 | 1.0000 |
|  |  | rs34299559 | 0.9502 | 1.0000 | 0.8256 | 0.9819 | 0.9742 | 1.0000 |
|  |  | rs16943453 | 1.0000 | 1.0000 | 0.8353 | 1.0000 | 1.0000 | 1.0000 |
|  |  | rs7174288 | 1.0000 | 1.0000 | 0.9771 | 1.0000 | 0.9972 | 1.0000 |
|  |  | rs11637844 | 1.0000 | 1.0000 | 0.9986 | 1.0000 | 1.0000 | 1.0000 |
|  |  | rs10519097 | 1.0000 | 1.0000 | 0.9874 | 1.0000 | 1.0000 | 1.0000 |
|  |  | rs17204770 | 0.8604 | 0.9152 | 0.9456 | 1.0000 | 0.8881 | 0.9861 |
|  |  | rs2030619 | 0.8604 | 0.9587 | 0.9671 | 1.0000 | 0.8722 | 0.9861 |
|  |  | rs11638929 | 0.9743 | 1.0000 | 0.7844 | 0.7980 | 1.0000 | 1.0000 |
|  |  | rs17237563 | 1.0000 | 1.0000 | 0.8587 | 1.0000 | 1.0000 | 1.0000 |
|  |  | rs1523530 | 0.9790 | 1.0000 | 0.9084 | 1.0000 | 1.0000 | 1.0000 |
|  |  | rs62005615 | 0.9705 | 1.0000 | 0.7844 | 0.7440 | 0.9880 | 1.0000 |
|  |  | rs72625740 | 0.8604 | 0.9013 | 0.9781 | 1.0000 | 0.8722 | 0.9861 |
|  |  | rs60257905 | 0.9502 | 1.0000 | 0.8587 | 1.0000 | 0.9609 | 1.0000 |
|  |  | rs17237570 | 0.8604 | 0.9687 | 0.7844 | 0.7987 | 0.9609 | 1.0000 |
|  |  | rs8040930 | 0.8604 | 0.9101 | 0.9863 | 1.0000 | 0.8722 | 0.9861 |
|  |  | rs12898479 | 0.8604 | 0.9013 | 0.9833 | 1.0000 | 0.8722 | 0.9861 |
|  |  | rs12592612 | 0.9050 | 0.9881 | 0.7844 | 0.7871 | 0.9546 | 1.0000 |
|  |  | rs17303341 | 0.9743 | 1.0000 | 0.9657 | 1.0000 | 0.9972 | 1.0000 |
|  |  | rs75336871 | 0.9705 | 1.0000 | 0.9594 | 1.0000 | 0.9939 | 1.0000 |
|  |  | rs17303355 | 0.8604 | 0.9013 | 0.9806 | 1.0000 | 0.8722 | 0.9861 |
|  |  | rs4775349 | 1.0000 | 1.0000 | 0.7883 | 0.8348 | 0.8881 | 0.9861 |
|  |  | rs72625742 | 0.8604 | 0.9013 | 0.9787 | 1.0000 | 0.8722 | 0.9861 |
|  |  | rs1403739 | 0.8604 | 0.9013 | 0.9787 | 1.0000 | 0.8722 | 0.9861 |
|  |  | rs17303369 | 0.9705 | 1.0000 | 0.8015 | 0.9380 | 0.9609 | 1.0000 |
|  |  | rs6494243 | 0.8604 | 0.9013 | 0.8015 | 0.8696 | 0.8722 | 0.9861 |
|  |  | rs12438879 | 0.9743 | 1.0000 | 0.9594 | 1.0000 | 0.9609 | 1.0000 |
|  |  | rs74687025 | 0.9940 | 1.0000 | 0.8256 | 0.9836 | 1.0000 | 1.0000 |
|  |  | rs10519107 | 0.9705 | 1.0000 | 0.9612 | 1.0000 | 0.9609 | 1.0000 |
|  |  | rs116919391 | 1.0000 | 1.0000 | 0.9986 | 1.0000 | 1.0000 | 1.0000 |
|  |  | rs809736 | 0.8604 | 0.9013 | 0.8015 | 0.9467 | 0.8881 | 0.9861 |
|  |  | rs2280595 | 0.9705 | 1.0000 | 0.9854 | 1.0000 | 0.9880 | 1.0000 |
|  |  | rs4775350 | 0.9927 | 1.0000 | 0.8354 | 1.0000 | 0.9609 | 1.0000 |
|  |  | rs1437549 | 1.0000 | 1.0000 | 0.9594 | 1.0000 | 1.0000 | 1.0000 |
|  |  | rs4775351 | 0.9502 | 1.0000 | 0.9493 | 1.0000 | 0.9546 | 1.0000 |
|  |  | rs7172342 | 0.9502 | 1.0000 | 0.8545 | 1.0000 | 0.9609 | 1.0000 |
|  |  | rs4774384 | 1.0000 | 1.0000 | 0.9771 | 1.0000 | 0.9880 | 1.0000 |
|  |  | rs782944 | 1.0000 | 1.0000 | 0.9863 | 1.0000 | 1.0000 | 1.0000 |
|  |  | rs10519108 | 1.0000 | 1.0000 | 0.7844 | 0.8208 | 1.0000 | 1.0000 |
|  |  | rs782948 | 0.8604 | 0.9013 | 0.8784 | 1.0000 | 0.8722 | 0.9861 |
|  |  | rs2247306 | 0.9502 | 1.0000 | 0.8015 | 0.9427 | 0.9880 | 1.0000 |
|  |  | rs12324535 | 0.9705 | 1.0000 | 0.8587 | 1.0000 | 1.0000 | 1.0000 |
|  |  | rs873962 | 0.9502 | 1.0000 | 0.9771 | 1.0000 | 0.9609 | 1.0000 |
|  |  | rs873961 | 0.9705 | 1.0000 | 0.8976 | 1.0000 | 0.9609 | 1.0000 |
|  |  | rs8037669 | 1.0000 | 1.0000 | 0.9095 | 1.0000 | 0.9880 | 1.0000 |
|  |  | rs7173460 | 0.8604 | 0.9013 | 0.9594 | 1.0000 | 0.8722 | 0.9861 |
|  |  | rs6494246 | 1.0000 | 1.0000 | 0.9634 | 1.0000 | 1.0000 | 1.0000 |
|  |  | rs78100524 | 1.0000 | 1.0000 | 0.9594 | 1.0000 | 1.0000 | 1.0000 |
|  |  | rs782956 | 0.9502 | 1.0000 | 0.9456 | 1.0000 | 0.9546 | 1.0000 |
|  |  | rs11632352 | 1.0000 | 1.0000 | 0.8444 | 1.0000 | 1.0000 | 1.0000 |
|  |  | rs782903 | 0.9812 | 1.0000 | 0.9980 | 1.0000 | 0.9880 | 1.0000 |
|  |  | rs16943579 | 1.0000 | 1.0000 | 0.9169 | 1.0000 | 1.0000 | 1.0000 |
|  |  | rs11854619 | 0.9705 | 1.0000 | 0.9594 | 1.0000 | 0.9880 | 1.0000 |
|  |  | rs12915672 | 1.0000 | 1.0000 | 0.9986 | 1.0000 | 1.0000 | 1.0000 |
|  |  | rs12915830 | 1.0000 | 1.0000 | 0.9986 | 1.0000 | 1.0000 | 1.0000 |
|  |  | rs708680 | 0.9743 | 1.0000 | 0.7856 | 0.8279 | 1.0000 | 1.0000 |
|  |  | rs12903172 | 1.0000 | 1.0000 | 0.9671 | 1.0000 | 1.0000 | 1.0000 |
|  |  | rs2689352 | 0.8604 | 0.9013 | 0.7844 | 0.7588 | 0.9609 | 1.0000 |
|  |  | rs940222 | 0.9502 | 1.0000 | 0.9392 | 1.0000 | 0.9609 | 1.0000 |
|  |  | rs62005642 | 0.9502 | 1.0000 | 0.7844 | 0.7976 | 0.9609 | 1.0000 |
|  |  | rs7171287 | 1.0000 | 1.0000 | 0.8015 | 0.8574 | 1.0000 | 1.0000 |
|  |  | rs1437551 | 0.9502 | 1.0000 | 0.7844 | 0.8104 | 0.9609 | 1.0000 |
|  |  | rs77786240 | 0.8604 | 0.9013 | 0.8015 | 0.9170 | 0.8722 | 0.9861 |
|  |  | rs782907 | 1.0000 | 1.0000 | 0.9671 | 1.0000 | 1.0000 | 1.0000 |
|  |  | rs782908 | 0.9502 | 1.0000 | 0.9612 | 1.0000 | 0.9609 | 1.0000 |
|  |  | rs4774386 | 1.0000 | 1.0000 | 0.9863 | 1.0000 | 1.0000 | 1.0000 |
|  |  | rs893286 | 1.0000 | 1.0000 | 0.9771 | 1.0000 | 1.0000 | 1.0000 |
|  |  | rs718911 | 1.0000 | 1.0000 | 0.9671 | 1.0000 | 1.0000 | 1.0000 |
|  |  | rs76853459 | 0.9502 | 1.0000 | 0.9594 | 1.0000 | 0.9540 | 1.0000 |
|  |  | rs12902142 | 1.0000 | 1.0000 | 0.9986 | 1.0000 | 1.0000 | 1.0000 |
|  |  | rs4775356 | 0.9705 | 1.0000 | 0.9874 | 1.0000 | 0.9880 | 1.0000 |
|  |  | rs782910 | 0.9705 | 1.0000 | 0.8587 | 1.0000 | 0.9939 | 1.0000 |
|  |  | rs8035885 | 0.9502 | 1.0000 | 0.9890 | 1.0000 | 0.9609 | 1.0000 |
|  |  | rs76824799 | 0.9544 | 1.0000 | 0.9771 | 1.0000 | 0.9609 | 1.0000 |
|  |  | rs60094610 | 1.0000 | 1.0000 | 0.8015 | 0.8974 | 1.0000 | 1.0000 |
|  |  | rs1437535 | 1.0000 | 1.0000 | 0.9980 | 1.0000 | 1.0000 | 1.0000 |
|  |  | rs1437537 | 1.0000 | 1.0000 | 0.9874 | 1.0000 | 1.0000 | 1.0000 |
|  |  | rs8042259 | 1.0000 | 1.0000 | 0.9693 | 1.0000 | 0.9973 | 1.0000 |
|  |  | rs76431303 | 1.0000 | 1.0000 | 0.7883 | 0.8354 | 1.0000 | 1.0000 |
|  |  | rs3803479 | 1.0000 | 1.0000 | 0.8015 | 0.8675 | 0.9880 | 1.0000 |
|  |  | rs893288 | 0.9502 | 1.0000 | 0.9787 | 1.0000 | 0.9609 | 1.0000 |
|  |  | rs78573683 | 0.9502 | 1.0000 | 0.9771 | 1.0000 | 0.9546 | 1.0000 |
|  |  | rs13329643 | 0.8604 | 0.9013 | 0.9671 | 1.0000 | 0.8722 | 0.9861 |
|  |  | rs782915 | 0.9502 | 1.0000 | 0.9888 | 1.0000 | 0.9609 | 1.0000 |
|  |  | rs8024672 | 1.0000 | 1.0000 | 0.7844 | 0.7436 | 1.0000 | 1.0000 |
|  |  | rs782919 | 1.0000 | 1.0000 | 0.9224 | 1.0000 | 1.0000 | 1.0000 |
|  |  | rs782931 | 1.0000 | 1.0000 | 0.9456 | 1.0000 | 1.0000 | 1.0000 |
|  |  | rs782933 | 0.9705 | 1.0000 | 0.9986 | 1.0000 | 0.9609 | 1.0000 |
|  |  | rs782935 | 0.9502 | 1.0000 | 0.9986 | 1.0000 | 0.9546 | 1.0000 |
|  |  | rs17303474 | 1.0000 | 1.0000 | 0.9986 | 1.0000 | 1.0000 | 1.0000 |
|  |  | rs782937 | 0.9502 | 1.0000 | 0.9806 | 1.0000 | 0.9609 | 1.0000 |
|  |  | rs16943672 | 1.0000 | 1.0000 | 0.8353 | 1.0000 | 1.0000 | 1.0000 |
|  |  | rs4775360 | 1.0000 | 1.0000 | 0.9787 | 1.0000 | 1.0000 | 1.0000 |
|  |  | rs7183595 | 1.0000 | 1.0000 | 0.9634 | 1.0000 | 1.0000 | 1.0000 |
|  |  | rs11634887 | 1.0000 | 1.0000 | 0.9863 | 1.0000 | 1.0000 | 1.0000 |
|  |  | rs719006 | 1.0000 | 1.0000 | 0.9771 | 1.0000 | 1.0000 | 1.0000 |
|  |  | rs1160694 | 1.0000 | 1.0000 | 0.9671 | 1.0000 | 1.0000 | 1.0000 |
|  |  | rs1159814 | 1.0000 | 1.0000 | 0.9980 | 1.0000 | 1.0000 | 1.0000 |
|  |  | rs78512626 | 1.0000 | 1.0000 | 0.9863 | 1.0000 | 0.9976 | 1.0000 |
|  |  | rs9788699 | 1.0000 | 1.0000 | 0.9874 | 1.0000 | 1.0000 | 1.0000 |
|  |  | rs9788704 | 1.0000 | 1.0000 | 0.8015 | 0.9385 | 0.9609 | 1.0000 |
|  |  | rs11071587 | 1.0000 | 1.0000 | 0.8923 | 1.0000 | 1.0000 | 1.0000 |
|  |  | rs11071588 | 1.0000 | 1.0000 | 0.9787 | 1.0000 | 1.0000 | 1.0000 |
|  |  | rs9788745 | 1.0000 | 1.0000 | 0.9771 | 1.0000 | 1.0000 | 1.0000 |
|  |  | rs7163680 | 0.9705 | 1.0000 | 0.9793 | 1.0000 | 0.9742 | 1.0000 |
|  |  | rs12900813 | 0.9812 | 1.0000 | 0.9793 | 1.0000 | 0.9880 | 1.0000 |
|  |  | rs12900948 | 0.9812 | 1.0000 | 0.9806 | 1.0000 | 0.9880 | 1.0000 |
|  |  | rs4238351 | 0.9705 | 1.0000 | 0.8015 | 0.9316 | 1.0000 | 1.0000 |
|  |  | rs12592385 | 0.9502 | 1.0000 | 0.9793 | 1.0000 | 0.9546 | 1.0000 |
|  |  | rs12900176 | 0.9705 | 1.0000 | 0.9926 | 1.0000 | 0.9609 | 1.0000 |
|  |  | rs737112 | 0.9743 | 1.0000 | 0.9874 | 1.0000 | 0.9609 | 1.0000 |
|  |  | rs17237759 | 1.0000 | 1.0000 | 0.8015 | 0.9361 | 1.0000 | 1.0000 |
|  |  | rs17303509 | 1.0000 | 1.0000 | 0.9986 | 1.0000 | 1.0000 | 1.0000 |
|  |  | rs1370433 | 0.9502 | 1.0000 | 0.9456 | 1.0000 | 0.9742 | 1.0000 |
|  |  | rs117080246 | 1.0000 | 1.0000 | 0.9081 | 1.0000 | 1.0000 | 1.0000 |
|  |  | rs17303523 | 0.8604 | 0.9013 | 0.9671 | 1.0000 | 0.8722 | 0.9861 |
|  |  | rs17303530 | 0.8604 | 0.9013 | 0.9671 | 1.0000 | 0.8722 | 0.9861 |
|  |  | rs4775368 | 0.9502 | 1.0000 | 0.9874 | 1.0000 | 0.9546 | 1.0000 |
|  |  | rs11071590 | 0.9502 | 1.0000 | 0.9787 | 1.0000 | 0.9546 | 1.0000 |
|  |  | rs11071591 | 0.9502 | 1.0000 | 0.9612 | 1.0000 | 0.9546 | 1.0000 |
|  |  | rs7171405 | 1.0000 | 1.0000 | 0.8444 | 1.0000 | 0.9880 | 1.0000 |
|  |  | rs4774388 | 0.9868 | 1.0000 | 0.9752 | 1.0000 | 0.9985 | 1.0000 |
|  |  | rs4775370 | 1.0000 | 1.0000 | 0.9771 | 1.0000 | 1.0000 | 1.0000 |
|  |  | rs1816624 | 1.0000 | 1.0000 | 0.9594 | 1.0000 | 1.0000 | 1.0000 |
|  |  | rs4774390 | 1.0000 | 1.0000 | 0.9491 | 1.0000 | 1.0000 | 1.0000 |
|  |  | rs4775371 | 0.8604 | 0.9013 | 0.7844 | 0.8107 | 0.8722 | 0.9861 |
|  |  | rs1370431 | 0.9705 | 1.0000 | 0.9806 | 1.0000 | 0.9609 | 1.0000 |
|  |  | rs12324086 | 0.9705 | 1.0000 | 0.9538 | 1.0000 | 0.9609 | 1.0000 |
|  |  | rs17204952 | 0.8604 | 0.9636 | 0.8015 | 0.8504 | 0.8722 | 0.9861 |
|  |  | rs17204959 | 1.0000 | 1.0000 | 0.9863 | 1.0000 | 1.0000 | 1.0000 |
|  |  | rs12441507 | 1.0000 | 1.0000 | 0.9833 | 1.0000 | 1.0000 | 1.0000 |
|  |  | rs1025676 | 1.0000 | 1.0000 | 0.9874 | 1.0000 | 1.0000 | 1.0000 |
|  |  | rs55870008 | 1.0000 | 1.0000 | 0.9943 | 1.0000 | 0.9972 | 1.0000 |
|  |  | rs7177846 | 1.0000 | 1.0000 | 0.9854 | 1.0000 | 0.9880 | 1.0000 |
|  |  | rs2277557 | 1.0000 | 1.0000 | 0.9986 | 1.0000 | 1.0000 | 1.0000 |
|  |  | rs10851691 | 1.0000 | 1.0000 | 0.9874 | 1.0000 | 1.0000 | 1.0000 |
|  |  | rs10519116 | 0.9502 | 1.0000 | 0.8015 | 0.9468 | 0.9609 | 1.0000 |
|  |  | rs726913 | 1.0000 | 1.0000 | 0.9793 | 1.0000 | 1.0000 | 1.0000 |
|  |  | rs726955 | 0.9705 | 1.0000 | 0.8256 | 0.9781 | 0.9609 | 1.0000 |
|  |  | rs2118326 | 1.0000 | 1.0000 | 0.8587 | 1.0000 | 1.0000 | 1.0000 |
|  |  | rs34720147 | 1.0000 | 1.0000 | 0.9787 | 1.0000 | 1.0000 | 1.0000 |
|  |  | rs4775374 | 1.0000 | 1.0000 | 0.8587 | 1.0000 | 1.0000 | 1.0000 |
|  |  | rs17204973 | 0.9812 | 1.0000 | 0.9491 | 1.0000 | 0.9880 | 1.0000 |
|  |  | rs1550226 | 1.0000 | 1.0000 | 0.9634 | 1.0000 | 1.0000 | 1.0000 |
|  |  | rs11631786 | 0.9502 | 1.0000 | 0.9793 | 1.0000 | 0.9546 | 1.0000 |
|  |  | rs11637553 | 1.0000 | 1.0000 | 0.8015 | 0.9095 | 1.0000 | 1.0000 |
|  |  | rs12900971 | 0.9502 | 1.0000 | 0.7844 | 0.7799 | 1.0000 | 1.0000 |
|  |  | rs146660446 | 0.9705 | 1.0000 | 0.7844 | 0.7360 | 0.9880 | 1.0000 |
| *RORB* | 9 | rs17293191 | 1.0000 | 1.0000 | 0.8587 | 1.0000 | 1.0000 | 1.0000 |
|  |  | rs4098048 | 1.0000 | 1.0000 | 0.8015 | 0.9499 | 1.0000 | 1.0000 |
|  |  | rs13293006 | 0.8604 | 0.9013 | 0.9863 | 1.0000 | 0.8722 | 0.9861 |
|  |  | rs28672222 | 1.0000 | 1.0000 | 0.8015 | 0.8674 | 1.0000 | 1.0000 |
|  |  | rs1018584 | 0.9705 | 1.0000 | 0.7844 | 0.7360 | 0.9880 | 1.0000 |
|  |  | rs4745330 | 0.8604 | 0.9379 | 0.9634 | 1.0000 | 0.9026 | 0.9861 |
|  |  | rs7042950 | 0.8604 | 0.9013 | 0.8015 | 0.8489 | 0.8722 | 0.9861 |
|  |  | rs10869418 | 0.8993 | 0.9781 | 0.8015 | 0.9174 | 0.9540 | 1.0000 |
|  |  | rs17611535 | 0.9502 | 1.0000 | 0.9874 | 1.0000 | 0.9609 | 1.0000 |
|  |  | rs7037043 | 0.9940 | 1.0000 | 0.7844 | 0.8123 | 1.0000 | 1.0000 |
|  |  | rs75657768 | 0.9502 | 1.0000 | 0.7856 | 0.8293 | 0.9609 | 1.0000 |
|  |  | rs972903 | 0.8604 | 0.9013 | 0.7455 | 0.7360 | 0.8722 | 0.9861 |
|  |  | rs972902 | 0.8604 | 0.9013 | 0.6968 | 0.7345 | 0.8722 | 0.9861 |
|  |  | rs17612113 | 0.9502 | 1.0000 | 0.9986 | 1.0000 | 0.9609 | 1.0000 |
|  |  | rs1323354 | 0.9705 | 1.0000 | 0.8015 | 0.8532 | 0.9609 | 1.0000 |
|  |  | rs62554058 | 0.8604 | 0.9361 | 0.7844 | 0.7460 | 0.9030 | 0.9861 |
|  |  | rs67022110 | 0.8604 | 0.9297 | 0.9594 | 1.0000 | 0.9026 | 0.9861 |
|  |  | rs10869430 | 0.9502 | 1.0000 | 0.8015 | 0.8988 | 1.0000 | 1.0000 |
|  |  | rs75206074 | 0.9502 | 1.0000 | 0.9863 | 1.0000 | 0.9546 | 1.0000 |
|  |  | rs1157358 | 0.9502 | 1.0000 | 0.8587 | 1.0000 | 0.9609 | 1.0000 |
|  |  | rs11144029 | 0.9705 | 1.0000 | 0.7844 | 0.7839 | 1.0000 | 1.0000 |
|  |  | rs3750420 | 0.9502 | 1.0000 | 0.9771 | 1.0000 | 0.9540 | 1.0000 |
|  |  | rs1013078 | 0.9502 | 1.0000 | 0.9771 | 1.0000 | 0.9609 | 1.0000 |
|  |  | rs10512037 | 0.8604 | 0.9013 | 0.8587 | 1.0000 | 0.8722 | 0.9861 |
|  |  | rs11144032 | 0.9502 | 1.0000 | 0.7844 | 0.8177 | 0.9679 | 1.0000 |
|  |  | rs2273975 | 1.0000 | 1.0000 | 0.9084 | 1.0000 | 1.0000 | 1.0000 |
|  |  | rs1319551 | 1.0000 | 1.0000 | 0.7844 | 0.8195 | 1.0000 | 1.0000 |
|  |  | rs10869433 | 1.0000 | 1.0000 | 0.9456 | 1.0000 | 0.9972 | 1.0000 |
|  |  | rs11144039 | 0.8604 | 0.9013 | 0.7844 | 0.7968 | 0.8881 | 0.9861 |
|  |  | rs72614684 | 0.8604 | 0.9013 | 0.8015 | 0.8532 | 0.8881 | 0.9861 |
|  |  | rs59894901 | 0.9502 | 1.0000 | 0.7844 | 0.7360 | 0.9609 | 1.0000 |
|  |  | rs10781247 | 1.0000 | 1.0000 | 0.9787 | 1.0000 | 0.9939 | 1.0000 |
|  |  | rs11144045 | 1.0000 | 1.0000 | 0.9874 | 1.0000 | 1.0000 | 1.0000 |
|  |  | rs7865407 | 1.0000 | 1.0000 | 0.9787 | 1.0000 | 1.0000 | 1.0000 |
|  |  | rs10869435 | 1.0000 | 1.0000 | 0.9863 | 1.0000 | 1.0000 | 1.0000 |
|  |  | rs10869436 | 1.0000 | 1.0000 | 0.9771 | 1.0000 | 1.0000 | 1.0000 |
|  |  | rs11144053 | 0.9502 | 1.0000 | 0.8354 | 1.0000 | 0.9026 | 0.9861 |
|  |  | rs3818559 | 1.0000 | 1.0000 | 0.9863 | 1.0000 | 1.0000 | 1.0000 |
|  |  | rs3793517 | 0.9790 | 1.0000 | 0.9095 | 1.0000 | 1.0000 | 1.0000 |
|  |  | rs10521463 | 0.9502 | 1.0000 | 0.9403 | 1.0000 | 0.9609 | 1.0000 |
|  |  | rs11144064 | 0.9502 | 1.0000 | 0.9538 | 1.0000 | 0.9742 | 1.0000 |
| *RORC* | 1 | rs9017 | 0.9502 | 1.0000 | 0.9806 | 1.0000 | 0.9609 | 1.0000 |
|  |  | rs9826 | 0.9502 | 1.0000 | 0.7844 | 0.8142 | 0.9880 | 1.0000 |
|  |  | rs3828057 | 0.9502 | 1.0000 | 0.8444 | 1.0000 | 0.9679 | 1.0000 |
|  |  | rs12045886 | 0.9705 | 1.0000 | 0.8410 | 1.0000 | 1.0000 | 1.0000 |
|  |  | rs1521177 | 1.0000 | 1.0000 | 0.7844 | 0.8083 | 1.0000 | 1.0000 |
|  |  | rs10494269 | 1.0000 | 1.0000 | 0.8015 | 0.9408 | 1.0000 | 1.0000 |
|  |  | rs7540530 | 1.0000 | 1.0000 | 0.9793 | 1.0000 | 1.0000 | 1.0000 |
|  |  | rs6693413 | 1.0000 | 1.0000 | 0.9787 | 1.0000 | 1.0000 | 1.0000 |
|  |  | rs11582525 | 0.9502 | 1.0000 | 0.8976 | 1.0000 | 0.9609 | 1.0000 |
|  |  | rs7531041 | 1.0000 | 1.0000 | 0.9771 | 1.0000 | 1.0000 | 1.0000 |
|  |  | rs72692783 | 1.0000 | 1.0000 | 0.8015 | 0.8862 | 1.0000 | 1.0000 |
| *SENP3* | 17 | rs8068222 | 1.0000 | 1.0000 | 0.9253 | 1.0000 | 1.0000 | 1.0000 |
|  |  | rs4602096 | 0.9502 | 1.0000 | 0.9084 | 1.0000 | 0.9880 | 1.0000 |
|  |  | rs4968213 | 1.0000 | 1.0000 | 0.8444 | 1.0000 | 1.0000 | 1.0000 |
|  |  | rs10468481 | 0.9502 | 1.0000 | 0.8361 | 1.0000 | 0.9880 | 1.0000 |
|  |  | rs114255812 | 1.0000 | 1.0000 | 0.9874 | 1.0000 | 0.9972 | 1.0000 |
| *SERPINE1* | 7 | rs2227631 | 0.9705 | 1.0000 | 0.8354 | 1.0000 | 1.0000 | 1.0000 |
|  |  | rs2227672 | 0.9502 | 1.0000 | 0.9538 | 1.0000 | 0.9609 | 1.0000 |
|  |  | rs2227690 | 0.9502 | 1.0000 | 0.9594 | 1.0000 | 0.9609 | 1.0000 |
|  |  | rs1050955 | 0.9502 | 1.0000 | 0.9771 | 1.0000 | 0.9546 | 1.0000 |
| *TIMELESS* | 12 | rs2291739 | 1.0000 | 1.0000 | 0.9806 | 1.0000 | 0.9880 | 1.0000 |
|  |  | rs774048 | 0.9502 | 1.0000 | 0.8587 | 1.0000 | 0.9546 | 1.0000 |
|  |  | rs774027 | 1.0000 | 1.0000 | 0.9771 | 1.0000 | 1.0000 | 1.0000 |
|  |  | rs7302060 | 1.0000 | 1.0000 | 0.9446 | 1.0000 | 1.0000 | 1.0000 |
|  |  | rs774045 | 0.8604 | 0.9013 | 0.9771 | 1.0000 | 0.8722 | 0.9861 |
|  |  | rs3809125 | 1.0000 | 1.0000 | 0.8587 | 1.0000 | 1.0000 | 1.0000 |
| *TIPIN* | 15 | rs3759786 | 1.0000 | 1.0000 | 0.8587 | 1.0000 | 1.0000 | 1.0000 |
| *VIP* | 6 | rs601240 | 0.9705 | 1.0000 | 0.9926 | 1.0000 | 0.9880 | 1.0000 |
|  |  | rs17083008 | 1.0000 | 1.0000 | 0.9890 | 1.0000 | 1.0000 | 1.0000 |
|  |  | rs73013001 | 1.0000 | 1.0000 | 0.9986 | 1.0000 | 1.0000 | 1.0000 |
|  |  | rs12212849 | 0.9502 | 1.0000 | 0.9986 | 1.0000 | 0.9609 | 1.0000 |
|  |  | rs3823082 | 1.0000 | 1.0000 | 0.9986 | 1.0000 | 1.0000 | 1.0000 |
|  |  | rs12201030 | 0.8604 | 0.9013 | 0.9986 | 1.0000 | 0.8722 | 0.9861 |
| *VIPR2* | 7 | rs6950938 | 0.8604 | 0.9013 | 0.9671 | 1.0000 | 0.8722 | 0.9861 |
|  |  | rs2730254 | 1.0000 | 1.0000 | 0.7844 | 0.7360 | 1.0000 | 1.0000 |
|  |  | rs2540359 | 1.0000 | 1.0000 | 0.7844 | 0.7360 | 1.0000 | 1.0000 |
|  |  | rs7784586 | 1.0000 | 1.0000 | 0.7844 | 0.7382 | 1.0000 | 1.0000 |
|  |  | rs56236179 | 1.0000 | 1.0000 | 0.7844 | 0.7360 | 1.0000 | 1.0000 |
|  |  | rs78219996 | 1.0000 | 1.0000 | 0.9980 | 1.0000 | 1.0000 | 1.0000 |
|  |  | rs3793217 | 1.0000 | 1.0000 | 0.8410 | 1.0000 | 1.0000 | 1.0000 |
|  |  | rs3793223 | 0.9705 | 1.0000 | 0.7844 | 0.7566 | 0.9972 | 1.0000 |
|  |  | rs2540352 | 0.8604 | 0.9013 | 0.7844 | 0.7360 | 0.8722 | 0.9861 |
|  |  | rs10263510 | 1.0000 | 1.0000 | 0.9793 | 1.0000 | 0.9972 | 1.0000 |
|  |  | rs55683317 | 1.0000 | 1.0000 | 0.7844 | 0.8106 | 1.0000 | 1.0000 |
|  |  | rs3793227 | 1.0000 | 1.0000 | 0.7844 | 0.7542 | 1.0000 | 1.0000 |
|  |  | rs2270314 | 0.9502 | 1.0000 | 0.7844 | 0.7360 | 0.9742 | 1.0000 |
|  |  | rs3793232 | 1.0000 | 1.0000 | 0.8587 | 1.0000 | 1.0000 | 1.0000 |
|  |  | rs17837875 | 1.0000 | 1.0000 | 0.7844 | 0.7835 | 1.0000 | 1.0000 |
|  |  | rs6974127 | 1.0000 | 1.0000 | 0.8587 | 1.0000 | 1.0000 | 1.0000 |
|  |  | rs399867 | 0.8604 | 0.9013 | 0.7844 | 0.7360 | 0.8788 | 0.9861 |
|  |  | rs6950857 | 1.0000 | 1.0000 | 0.7844 | 0.7423 | 1.0000 | 1.0000 |
|  |  | rs3793237 | 1.0000 | 1.0000 | 0.7844 | 0.7636 | 1.0000 | 1.0000 |
|  |  | rs3793239 | 1.0000 | 1.0000 | 0.9671 | 1.0000 | 1.0000 | 1.0000 |
|  |  | rs74790021 | 1.0000 | 1.0000 | 0.7844 | 0.7530 | 1.0000 | 1.0000 |
|  |  | rs6459928 | 1.0000 | 1.0000 | 0.9594 | 1.0000 | 1.0000 | 1.0000 |
|  |  | rs3812310 | 0.8604 | 0.9077 | 0.8015 | 0.9481 | 0.9374 | 0.9876 |
|  |  | rs3812311 | 0.8604 | 0.9430 | 0.8176 | 0.9607 | 0.9540 | 1.0000 |
|  |  | rs3812313 | 0.9790 | 1.0000 | 0.8015 | 0.8421 | 1.0000 | 1.0000 |

Chr = chromosome, FDR = false discovery rate, MetS = metabolic syndrome.
