# Supplementary material for: Effects of circadian clock genes and health-related behavior on metabolic syndrome in a Taiwanese population: Evidence from association and interaction analysis
Source: PLoS One. 2017 Mar 15;12(3):e0173861. doi: 10.1371/journal.pone.0173861 (PMC5352001; doi:10.1371/journal.pone.0173861)
Supplement: S4 Table — (DOC) [file pone.0173861.s004.doc]

**S4 Table.** Q values and FDRs for odds ratio analysis between individual components of the MetS and five key SNPs in the five circadian clock genes (including *ARNTL* rs10832020, *GSK3B* rs2199503, *PER3* rs10746473, *RORA* rs8034880, and *RORB* rs972902).

| Individual components of the MetS | Additive model | | Recessive model | | Dominant model | |
| --- | --- | --- | --- | --- | --- | --- |
|  | Q value | FDR | Q value | FDR | Q value | FDR |
| (1) *ARNTL* rs10832020 | | | | | | |
| High waist circumference^a^ | 0.3588 | 0.8284 | 0.0761 | 0.1606 | 0.6945 | 1.0000 |
| High triglyceride^b^ | 0.6633 | 1.0000 | 0.1723 | 0.3483 | 0.7105 | 1.0000 |
| Low HDL^c^ | 0.3054 | 0.6500 | 0.0761 | 0.1648 | 0.7933 | 1.0000 |
| High blood pressure^d^ | 0.6633 | 1.0000 | 0.2164 | 0.3852 | 0.7105 | 1.0000 |
| High fasting glucose^e^ | 0.4091 | 1.0000 | 0.1587 | 0.3131 | 0.5025 | 1.0000 |
| (2) *GSK3B* rs2199503 | | | | | | |
| High waist circumference^a^ | 0.3588 | 0.8865 | 0.1587 | 0.2777 | 0.5193 | 1.0000 |
| High triglyceride^b^ | 0.7932 | 1.0000 | 0.2326 | 0.3855 | 0.7933 | 1.0000 |
| Low HDL^c^ | 0.7722 | 1.0000 | 0.1587 | 0.3132 | 0.3019 | 0.6408 |
| High blood pressure^d^ | 0.6633 | 1.0000 | 0.2044 | 0.3772 | 0.7933 | 1.0000 |
| High fasting glucose^e^ | 0.0040 | 0.0140 | 0.0019 | 0.0093 | 0.1875 | 0.4558 |
| (3) *PER3* rs10746473 | | | | | | |
| High waist circumference^a^ | 0.0182 | 0.0649 | 0.0750 | 0.1272 | 0.0325 | 0.0780 |
| High triglyceride^b^ | 0.7525 | 1.0000 | 0.1723 | 0.3449 | 0.7105 | 1.0000 |
| Low HDL^c^ | 0.1785 | 0.3713 | 0.1317 | 0.2464 | 0.1875 | 0.3225 |
| High blood pressure^d^ | 0.3320 | 0.7258 | 0.1587 | 0.3003 | 0.3564 | 0.7441 |
| High fasting glucose^e^ | 0.5801 | 1.0000 | 0.0750 | 0.1256 | 0.5025 | 1.0000 |
| (4) *RORA* rs8034880 | | | | | | |
| High waist circumference^a^ | 0.6633 | 1.0000 | 0.2326 | 0.3855 | 0.1875 | 0.4452 |
| High triglyceride^b^ | 0.5689 | 1.0000 | 0.1723 | 0.3514 | 0.5473 | 1.0000 |
| Low HDL^c^ | 0.3588 | 0.8456 | 0.1317 | 0.2447 | 0.6303 | 1.0000 |
| High blood pressure^d^ | 0.3054 | 0.5903 | 0.1219 | 0.2163 | 0.1875 | 0.4610 |
| High fasting glucose^e^ | 0.7525 | 1.0000 | 0.2214 | 0.3855 | 0.1875 | 0.2794 |
| (5) *RORB* rs972902 | | | | | | |
| High waist circumference^a^ | 0.7459 | 1.0000 | 0.2044 | 0.3794 | 0.6585 | 1.0000 |
| High triglyceride^b^ | 0.4257 | 1.0000 | 0.1721 | 0.3307 | 0.1875 | 0.4310 |
| Low HDL^c^ | 0.4091 | 0.9988 | 0.1587 | 0.2892 | 0.5025 | 1.0000 |
| High blood pressure^d^ | 0.3054 | 0.6175 | 0.0761 | 0.1476 | 0.6945 | 1.0000 |
| High fasting glucose^e^ | 0.1008 | 0.2354 | 0.0560 | 0.0827 | 0.5025 | 0.9746 |

FDR = false discovery rate, HDL = high-density lipoprotein cholesterol, MetS = metabolic syndrome.

^a^ Waist circumference ≥ 90 cm in male subjects, waist circumference ≥ 80 cm in female subjects.

^b^ Triglyceride ≥ 150 mg/dl.

^c^ HDL< 40 mg/dl in male subjects, HDL < 50 mg/dl in female subjects.

^d^ Systolic blood pressure ≥ 130 mmHg or diastolic blood pressure ≥ 85 mmHg.

^e^ Fasting glucose ≥ 100 mg/dl.
