# Supplementary material for: Effects of circadian clock genes and health-related behavior on metabolic syndrome in a Taiwanese population: Evidence from association and interaction analysis
Source: PLoS One. 2017 Mar 15;12(3):e0173861. doi: 10.1371/journal.pone.0173861 (PMC5352001; doi:10.1371/journal.pone.0173861)
Supplement: S5 Table — (DOC) [file pone.0173861.s005.doc]

**S5 Table.** Q values and FDRs for two-way gene-gene interaction models by using the GMDR method.

| Phenotype | Two-way interaction model | Q value | FDR |
| --- | --- | --- | --- |
| MetS | *ARNTL* rs10832020, *GSK3B* rs2199503 | 0.0390 | 0.2379 |
|  | *ARNTL* rs10832020, *PER3* rs10746473 | 0.0300 | 0.1212 |
|  | *ARNTL* rs10832020, *RORA* rs8034880 | 0.0025 | 0.0076 |
|  | *ARNTL* rs10832020, *RORB* rs972902 | 0.0356 | 0.1883 |
|  | *GSK3B* rs2199503, *PER3* rs10746473 | 0.0338 | 0.1564 |
|  | *GSK3B* rs2199503, *RORA* rs8034880 | 0.0025 | 0.0076 |
|  | *GSK3B* rs2199503, *RORB* rs972902 | 0.0040 | 0.0134 |
|  | *PER3* rs10746473, *RORA* rs8034880 | 0.0025 | 0.0076 |
|  | *PER3* rs10746473, *RORB* rs972902 | 0.0025 | 0.0076 |
|  | *RORA* rs8034880, *RORB* rs972902 | 0.0133 | 0.0492 |

FDR = false discovery rate, GMDR = generalized multifactor dimensionality reduction, MetS = metabolic syndrome.

**Table 5.** Q-values and FDRs for gene-environment interaction models identified by the GMDR method.

| Phenotype | Best interaction model | Q-value | FDR |
| --- | --- | --- | --- |
| (a) Two-way interaction models | | | |
| MetS | *RORB* rs972902, smoking | 0.0030 | 0.0112 |
| High waist circumference^a^ | *ARNTL* rs10832020, physical activity | 0.0948 | 0.5550 |
| High triglyceride^b^ | *RORB* rs972902, smoking | 0.0874 | 0.4842 |
| Low HDL^c^ | *RORB* rs972902, physical activity | 0.0030 | 0.0112 |
| High blood pressure^d^ | *RORA* rs8034880, physical activity | 0.0622 | 0.2879 |
| High fasting glucose^e^ | *PER3* rs10746473, alcohol consumption | 0.0068 | 0.0280 |
| (b) Three-way interaction models | | | |
| MetS | *PER3* rs10746473, *RORB* rs972902, smoking | 0.0030 | 0.0112 |
| High waist circumference^a^ | *ARNTL* rs10832020, *PER3* rs10746473, physical activity | 0.0872 | 0.4520 |
| High triglyceride^b^ | *ARNTL* rs10832020, *PER3* rs10746473, physical activity | 0.0288 | 0.1314 |
| Low HDL^c^ | *PER3* rs10746473, smoking, physical activity | 0.0030 | 0.0112 |
| High blood pressure^d^ | *PER3* rs10746473, *RORA* rs8034880, physical activity | 0.2800 | 1.0000 |
| High fasting glucose^e^ | *ARNTL* rs10832020, *GSK3B* rs2199503, physical activity | 0.0030 | 0.0112 |
| (c) Four-way interaction models | | | |
| MetS | *PER3* rs10746473, *RORA* rs8034880, *RORB* rs972902, smoking | 0.0160 | 0.0688 |
| High waist circumference^a^ | *ARNTL* rs10832020, *GSK3B* rs2199503, *PER3* rs10746473, physical activity | 0.0765 | 0.3741 |
| High triglyceride^b^ | *ARNTL* rs10832020, *PER3* rs10746473, *RORA* rs8034880, physical activity | 0.1890 | 1.0000 |
| Low HDL^c^ | *GSK3B* rs2199503, *PER3* rs10746473, *RORB* rs972902, physical activity | 0.0030 | 0.0112 |
| High blood pressure^d^ | *ARNTL* rs10832020, *PER3* rs10746473, *RORA* rs8034880, physical activity | 0.2340 | 1.0000 |
| High fasting glucose^e^ | *ARNTL* rs10832020, *GSK3B* rs2199503, *PER3* rs10746473, physical activity | 0.0051 | 0.0197 |

FDR = false discovery rate, GMDR = generalized multifactor dimensionality reduction, HDL = high-density lipoprotein cholesterol, MetS = metabolic syndrome.

^a^ Waist circumference ≥ 90 cm in male subjects, waist circumference ≥ 80 cm in female subjects.

^b^ Triglyceride ≥ 150 mg/dl.

^c^ HDL< 40 mg/dl in male subjects, HDL < 50 mg/dl in female subjects.

^d^ Systolic blood pressure ≥ 130 mmHg or diastolic blood pressure ≥ 85 mmHg.

^e^ Fasting glucose ≥ 100 mg/dl.
