# Supplementary material for: Effects of circadian clock genes and health-related behavior on metabolic syndrome in a Taiwanese population: Evidence from association and interaction analysis
Source: PLoS One. 2017 Mar 15;12(3):e0173861. doi: 10.1371/journal.pone.0173861 (PMC5352001; doi:10.1371/journal.pone.0173861)
Supplement: S6 Table — (DOC) [file pone.0173861.s006.doc]

**S6 Table.** Gene-gene interaction models identified by the GMDR method with adjustment for age and gender.

| Phenotype | Best interaction model | Testing accuracy (%) | P value |
| --- | --- | --- | --- |
| (1) Two-way interaction models | | | |
| MetS | *PER3* rs10746473, *RORB* rs972902 | 55.19 | **< 0.001** |
| High waist circumference^a^ | *ARNTL* rs10832020, *PER3* rs10746473 | 53.38 | 0.003 |
| High triglyceride^b^ | *ARNTL* rs10832020, *PER3* rs10746473 | 52.46 | 0.071 |
| Low HDL^c^ | *ARNTL* rs10832020, *GSK3B* rs2199503 | 51.00 | 0.270 |
| High blood pressure^d^ | *PER3* rs10746473, *RORA* rs8034880 | 52.33 | 0.073 |
| High fasting glucose^e^ | *GSK3B* rs2199503, *PER3* rs10746473 | 53.66 | 0.007 |
| (2) Three-way interaction models | | | |
| MetS | *ARNTL* rs10832020, *GSK3B* rs2199503, *RORA* rs8034880 | 54.68 | 0.003 |
| High waist circumference^a^ | *ARNTL* rs10832020, *GSK3B* rs2199503, *PER3* rs10746473 | 51.86 | 0.097 |
| High triglyceride^b^ | *ARNTL* rs10832020, *GSK3B* rs2199503, *PER3* rs10746473 | 53.13 | 0.040 |
| Low HDL^c^ | *GSK3B* rs2199503, *PER3* rs10746473, *RORB* rs972902 | 50.88 | 0.284 |
| High blood pressure^d^ | *ARNTL* rs10832020, *PER3* rs10746473, *RORA* rs8034880 | 52.33 | 0.093 |
| High fasting glucose^e^ | *GSK3B* rs2199503, *PER3* rs10746473, *RORB* rs972902 | 52.79 | 0.041 |
| (3) Four-way interaction models | | | |
| MetS | *ARNTL* rs10832020, *GSK3B* rs2199503, *PER3* rs10746473, *RORA* rs8034880 | 53.48 | 0.022 |
| High waist circumference^a^ | *ARNTL* rs10832020, *GSK3B* rs2199503, *PER3* rs10746473, *RORA* rs8034880 | 50.75 | 0.305 |
| High triglyceride^b^ | *ARNTL* rs10832020, *GSK3B* rs2199503, *PER3* rs10746473, *RORA* rs8034880 | 50.69 | 0.385 |
| Low HDL^c^ | *ARNTL* rs10832020, *PER3* rs10746473, *RORA* rs8034880, *RORB* rs972902 | 54.42 | 0.004 |
| High blood pressure^d^ | *ARNTL* rs10832020, *GSK3B* rs2199503, *PER3* rs10746473, *RORA* rs8034880 | 51.58 | 0.180 |
| High fasting glucose^e^ | *ARNTL* rs10832020, *GSK3B* rs2199503, *PER3* rs10746473, *RORB* rs972902 | 53.08 | 0.036 |

GMDR = generalized multifactor dimensionality reduction, HDL = high-density lipoprotein cholesterol, MetS = metabolic syndrome.

P value was based on 1,000 permutations. Analysis was obtained after adjustment for covariates including age and gender.

P values of < 0.0028 (Bonferroni correction: 0.05/18) are shown in bold.

^a^ Waist circumference ≥ 90 cm in male subjects, waist circumference ≥ 80 cm in female subjects.

^b^ Triglyceride ≥ 150 mg/dl.

^c^ HDL< 40 mg/dl in male subjects, HDL < 50 mg/dl in female subjects.

^d^ Systolic blood pressure ≥ 130 mmHg or diastolic blood pressure ≥ 85 mmHg.

^e^ Fasting glucose ≥ 100 mg/dl.
